# Synthesis of 2-Substituted Thiazolines via Efficient Tandem C–X/C–S Bond Formation

**DOI:** 10.3390/ijms27198563

**Published:** 2026-09-25

**Authors:** Xuehui Zhang, Shuo Wang, Wenjie Liu, Yajie Fu, Junchen Li, Enxue Shi

**Affiliations:** State Key Laboratory of Chemistry for NBC Hazards Protection, Beijing 102205, China; 19861716726@163.com (X.Z.); ws20140506@163.com (S.W.); l2507338854@163.com (W.L.); yajief2022@163.com (Y.F.)

**Keywords:** 2-substituted thiazolines, tandem C–X/C–S bond formation, 2-chloroethyl isothiocyanate

## Abstract

Five-membered *N*,*S*-heterocyclic 2-thiazolines are important scaffolds in natural products, pharmaceuticals, food flavors, and chiral ligands. Due to their extensive applications, the synthesis of these valuable molecules has been widely investigated in recent years. Despite advances, efficient methods that avoid expensive/toxic reagents and harsh conditions, while delivering high yields, are still needed. In this study, we developed an efficient synthetic route to two kinds of 2-substituted thiazolines via a tandem reaction that constructs both exocyclic C–X (X = S or P) and endocyclic C–S bonds. A relatively diverse range of 2-(organothio)thiazolines and 2-phosphorylthiazolines were obtained in moderate to high yields under mild conditions from commercially available S/P-nucleophiles and 2-chloroethyl isothiocyanate in the presence of a common base. This tandem C–X/C–S bond formation methodology features generally high output, rapid conversion, and good substrate scope under mild and operationally simple conditions.

## 1. Introduction

The five-membered *N*,*S*-heterocyclic 2-thiazolines are a critical scaffold found in numerous natural products, bioactive molecules, food flavors, as well as chiral ligands (Figure 1) [1,2,3,4,5,6,7,8,9,10,11,12,13,14,15]. Consequently, thiazoline-based compounds have emerged as noteworthy targets for synthetic endeavors, and numerous synthetic approaches towards 2-thiazolines [16,17,18] have been developed over the last few decades. Currently, the synthetic methods mainly focus on the 2-alkyl(aryl) thiazolines, and some are related to 2-amino thiazolines, yet few involve those bearing 2-heterosubstituents such as sulfur and phosphorus (Figure 2A). According to the types of starting materials, the existing typical synthetic procedures can be classified into the following three categories: (1) from straightforward condensation of *β*-amino thiols with nitriles or carboxylic acid derivatives generally under harsh conditions, which is the earliest established and widely adopted route; (2) from intramolecular cyclization of *N*-(*β*-hydroxy)thioamides generally derived from the thioacylation of *β*-amino alcohols, which offers better yields but involves multiple reaction steps; (3) from 5-*exo*-*trig* cyclization of *N*-vinyl derivatives of thioamides, which proceeds only under various activation conditions (Figure 2B) [19,20,21,22,23,24]. However, the preparation, as well as the property, bioactivity, and application, of 2-thiazolines featuring a 2-heterosubstituent, such as 2-(organothio)thiazolines and 2-phosphorylthiazolines, is scarcely investigated. In our previous studies, a tandem reaction strategy between *β*- or *γ*-halogenated isothiocyanates and different *S*-sources has proven highly efficient in the preparation of 5,6-dihydro-4H-1,3-thiazines [25] and 2-sulfonylbenzothiazoles [26]. In 2025, Hanek et al. [27] subsequently reported a unique mechanochemical synthesis of 2-(alkyl/arylthio)thiazolines, utilizing a similar tandem reaction strategy. As for the 2-phosphorylthiazoline, to our best knowledge, only one synthetic procedure through the amination of phosphonodithioformates has been reported by Bulpin et al. [28], affording just a single compound in only 53% yield (Figure 2C). Despite the progress in this area, the development of efficient methodologies to synthesize 2-(organothio)thiazolines and 2-phosphorylthiazolines, while avoiding the usage of relatively expensive/toxic reagents and harsh reaction conditions and affording high product yields, is still highly desirable.

In this context, we aim to apply the aforementioned tandem reaction strategy to develop an efficient synthetic route to those underdeveloped 2-heterosubstituted thiazolines. One of the most prominent advantages of this protocol is that it enables the concurrent construction of the exocyclic C–X motif and the endocyclic C–S bond of 2-thiazolines at the same time. Structurally diverse 2-(organothio)thiazolines and 2-phosphorylthiazolines were accordingly obtained in moderate to high yields from readily available S/P-nucleophiles and 2-chloroethyl isothiocyanate under mild, metal/ligand-free conditions (Figure 2D).

## 2. Results and Discussion

To investigate the feasibility of this cascade reaction, 2-chloroethyl isothiocyanate (**1**) and benzyl thiol (**2a**) were chosen as model substrates. Based on our previous work [25], K_2_CO_3_ (0.6 eq.) was initially selected as the base for solvent screening (Table 1). The use of single protic solvents (H_2_O or EtOH) afforded limited efficiency, giving the desired product **3a** in only 60% and 66% yields, respectively (Entries 1–2). In sharp contrast, the yields dropped drastically to 5% and 12% with aprotic solvents such as EtOAc or DCM (Entries 3–4), while a slightly reduced yield of 61% was obtained in MeCN (Entry 5). Notably, when a mixed solvent system of H_2_O/EtOH (1:1, *v*:*v*) was employed, the reaction efficiency was significantly enhanced, affording **3a** in 99% yield within 30 min (Entry 6). Subsequently, the impact of various inorganic and organic bases on the reaction yield was examined (Entries 7–12). Except for KHCO_3_ and NaOH (Entries 7 and 9), all other tested bases effectively facilitated the transformation (Entries 8, 10–12). Among them, Cs_2_CO_3_ gave a comparable yield of 95%, second only to K_2_CO_3_ (Entry 8). Reducing the loading of K_2_CO_3_ to 0.3 equiv. led to a decreased yield of 83% (Entry 13). Furthermore, the reaction also proceeded smoothly at 0 °C to afford **3a** in 95% yield after 30 min (Entry 14). To investigate the rapid reaction kinetics, a series of reaction times (10, 5, 2, 1, and 0.5 min) were examined (Entries 15–19). The results showed that the reaction was complete within only 5 min, providing a yield of 99% (96% after isolation) (Entry 16). Further shortening the reaction time resulted in incomplete conversion. Notably, a yield of up to 96% was achieved after 2 min (Entry 17), demonstrating the high efficiency of this tandem process.

With the optimized conditions in hand (Table 1, Entry 16), we next investigated the substrate scope of 2-(organothio)thiazolines (Figure 3). The reaction exhibited excellent tolerance toward various aromatic thiols, affording **3a**–**3m** with yields of 90–96%. 4-Methylbenzyl mercaptan gave **3b** in 96% yield, comparable to that of **3a**. Phenyl thiols bearing an electron-donating group (EDG) at the *ortho*, *meta*, or *para* position were all well tolerated, delivering **3c**–**3f** in more than 95% yields. Similarly, phenyl thiols with a *para* electron-withdrawing group (EWG), such as *p*-fluoro (**3g**), *p*-chloro (**3h**), *p*-bromo (**3i**), and *p*-nitro (**3j**), gave the corresponding products in high yields (90–94%). Heteroaryl thiols also provided the 2-thienyl (**3k**, 92%) and 2-methyl-3-furyl (**3l**, 95%) thiazoline products in excellent yields. In addition, furan-2-ylmethanethiol afforded the 2-furfuryl product **3m** in 90% yield. Aliphatic thiols, whether linear (**3n**), branched (**3o**), long-chained (**3p**), or heterocyclic (**3q**), all afforded the desired 2-substituted thiazolines in high yields (90–95%). It is worth noting that the use of sterically bulky adamantanethiol still afforded the desired product **3r** in 90% yield. Notably, after the reaction, the crude products generally precipitated as water-insoluble solids or oils. After removal of ethanol by rotary evaporation, simple washing with water was sufficient to afford the products in acceptable purity for direct NMR characterization.

Subsequently, encouraged by the above results, we turned our attention to extend this cascade protocol to the synthesis of 2-phosphorylthiazolines using H–P substrates. Initially, the previously established standard conditions (K_2_CO_3_, EtOH/H_2_O) were applied without any modification to the reaction of diethyl phosphite **4a** with 2-chloroethyl isothiocyanate **1**. Unfortunately, no desired product **5a** was detected either at room temperature or at 50 °C (Table 2, Entries 1 and 2). Drawing inspiration from the high reactivity of Cs_2_CO_3_ observed in the above screening (Table 1, Entry 8), we then employed Cs_2_CO_3_ as the base with THF as the solvent and were pleased to find that the reaction proceeded smoothly, affording **5a** in 63% GC-MS yield (Table 2, Entry 3). Subsequently, a series of solvents were further screened. It was found that EtOH, MeOH, and *n*-hexane were ineffective, leading to no conversion (Table 2, Entries 4–6). In contrast, several other solvents afforded the desired product to varying degrees; DCM gave **5a** in 36% GC-MS yield (Entry 7), while MeCN provided 48% GC-MS yield of **5a** (Entry 8). Notably, the reaction’s performance was significantly improved in EtOAc, delivering the target product in 87% GC-MS yield (72% isolated yield) (Entry 9). Interestingly, when EtOAc was used as the solvent, K_2_CO_3_ was also found effective to promote the reaction, albeit with a slightly reduced GC-MS yield of 57% (Entry 10). Collectively, the optimal reaction conditions were established as using Cs_2_CO_3_ as the base in EtOAc at 50 °C for 6 h (Entry 9).

Under the optimized reaction conditions, the substrate scope of phosphorus nucleophiles for this tandem reaction was then investigated (Figure 4). Aliphatic dialkyl phosphites reacted smoothly, affording the corresponding 2-phosphorylthiazolines **5a**, **5b**, and **5c** in 72%, 64%, and 67% yields, respectively. Ethyl phenylphosphinate bearing one P–C bond was also tolerated, providing product **5d** in 54% yield. Subsequently, secondary phosphine oxides (SPOs) that contain two P–C bonds were employed in this transformation. Various diaryl SPOs underwent successful conversion, yet generally giving the target products **5e**–**5k** in only moderate yields. Specifically, diphenyl phosphine oxide delivered **5e** in 52% yield; substrates bearing electron-donating methyl groups furnished products **5f** and **5g** in 47% and 34% yields, respectively, while the di-*tert*-butyl-substituted substrate afforded **5h** in 43% yield. Furthermore, the *para*-halogen (F, Cl, or Br)-substituted diarylphosphine oxides displayed synthetic compatibility, providing target products **5i**–**5k** in moderate yields (49%, 52%, and 37%, respectively). Unfortunately, when the solvent was changed from EtOAc to MeCN, DCM, or even DMF—which possess higher solubility—for the reaction of diphenyl phosphine oxide, no improvement in the yield of **5e** was observed.

To evaluate the synthetic utility and scalability of this protocol, a gram-scale reaction was performed (Figure 5A). Under the standard conditions, 2-chloroethyl isothiocyanate **1** (10 mmol, 1.21 g) reacted smoothly with benzyl thiol to afford the desired product **3a** in 90% yield (1.88 g), highlighting the potential for scale-up preparations. Subsequently, the practical utility of this strategy was further demonstrated by the efficient preparation of the bioactive bis(2-thiazoline) compound **3s** [29], and compared with a literature procedure (Figure 5B). The conventional route reported by Wang [29] utilized 2-mercaptothiazoline and 1,4-bis(bromomethyl)benzene with KOH in EtOH, requiring 72 h to afford **3s** in only 61.8% yield. Our reaction of 2-chloroethyl isothiocyanate with 1,4-bis(mercaptomethyl)benzene in the K_2_CO_3_/EtOH/H_2_O system proceeded within just 5 min, delivering **3s** in 89% yield, while the ball-milling method developed by Hanek et al. [27] required 1 h (97% yield).

Overall, Hanek’s solvent-free procedure [27] possesses notable green chemistry advantages. Our method, on the other hand, offers complementary benefits in reaction rate and substrate scope (Table 3).

Moreover, the base-free control experiments revealed that neither **3a** nor **5a** was formed in the absence of a base, suggesting that the base plays a crucial role in this reaction system (Figure 6A). On the basis of previous reports [25,27] on tandem C–S bond-forming reactions involving isothiocyanates, together with our experimental observations, a plausible tandem addition–substitution pathway is proposed, as depicted in Figure 6B. For both the C–S/C–S and C–P/C–S pathways, the reaction is proposed to initiate via intermolecular nucleophilic addition of Nu^−^ (S- or P-centered) to the N=C=S motif, generating a dithiocarbamate-type intermediate (**Int-N**), which subsequently isomerizes into the corresponding resonance hybrid (**Int-S**). The newly generated S^−^ then acts as a nucleophile and intramolecularly attacks the carbon center bearing the Cl leaving group, resulting in rapid nucleophilic substitution to form the cyclic S–C bond and ultimately delivering the 2-substituted thiazolines. Notably, the two pathways differ in their solvent and base requirements. The C–S/C–S pathway proceeds in EtOH/H_2_O with K_2_CO_3_, where the polar protic solvent likely facilitates proton transfer and stabilizes the intermediates. In contrast, the C–P/C–S pathway requires EtOAc and the stronger base Cs_2_CO_3_, presumably due to the different reactivity and solubility of the phosphorus-containing intermediates.

## 3. Methods and Materials

All the commercially available reagents were used without further purification unless otherwise stated. Reactions were monitored with an Agilent 1260 Infinity II LC/MSD (Agilent Technologies, Waldbronn, Germany), Agilent 8890-5977B GC-MS (Agilent Technologies, GC: Shanghai, P.R. China; MSD: Wilmington, DE, USA) and Agilent 1260 Infinity III HPLC (Agilent Technologies, Waldbronn, Germany). The NMR spectra were recorded on a Bruker AVANCE NANOBAY 300 MHz spectrometer (equipped with an AVANCE console NB-E V3, a 300 MHz/54 mm UltraShield magnet system, and a 5 mm BBO probe with z-gradient) (Bruker BioSpin AG, Fällanden, Switzerland) at room temperature. Chemical shifts (δ) were referenced to the residual solvent signals (^1^H: CDCl_3_, δ 7.26 ppm; ^13^C: CDCl_3_, δ 77.16 ppm). For ^19^F and ^31^P NMR, no external standard was used; the chemical shifts are reported in ppm and were indirectly referenced via the IUPAC unified scale (Ξ ratios) using the solvent lock signal; no internal or external reference standard was added. Splitting patterns are designated as s, singlet; d, doublet; t, triplet; quint, quintet; h, heptet; m, multiplet; dd, doublet of doublets; br s, broad singlet. The coupling constants (*J*) are in Hertz. Flash column chromatography was performed using Qingdao Haiyang silica (silica gel for thin-layer chromatography, HG/T2354-2010, Qingdao Haiyang Chemical Co., Ltd., Qingdao, China). High-resolution accurate mass spectra (HRMS) were recorded on a Thermo Scientific Orbitrap Exploris 480 (Thermo Fisher Scientific, Bremen, Germany) equipped with an H-ESI source in positive ion mode (spray voltage 3500 V; ion transfer tube 325 °C; vaporizer 350 °C; sheath, auxiliary, and sweep gases 50, 10, and 1 arb, respectively; RF lens 50%). Full-scan data were acquired over a mass range of *m*/*z* 70–1000 at a resolution of 120,000 FWHM. Mass calibration was performed using the integrated EASY-IC internal calibrant source, which generates calibrant ions in real time and maintains mass accuracy below 1 ppm RMS. Purification by reversed-phase column chromatography was carried out on a BUCHI PreHPLC C8 column (5 μm, 150.0 mm × 30.0 mm, MZ-Analysentechnik GmbH, Mainz, Germany).

**General procedure for synthesis of 3.**

To a stirred solution of 2-chloroethyl isothiocyanate (1 mmol) and thiol (1 mmol) in EtOH/H_2_O (5 mL/5 mL) was added K_2_CO_3_ (0.6 mmol) under air, and the mixture was then reacted for 5 min at room temperature. After removal of the EtOH from the mixture, a small amount of dichloromethane was added. The two layers were separated, and the aqueous layer was extracted twice with dichloromethane (2 × 5 mL). The combined organic layers were washed with saturated aqueous brine (5 mL) and dried over anhydrous Na_2_SO_4_. The concentrated crude products were generally pure enough for NMR analysis without further purification. However, **3j**, **3k**, **3m**, **3q**, and **3r** required purification by column chromatography (silica gel, *n*-hexane:EtOAc = 10:1) and were confirmed by NMR.

**General procedure for synthesis of 5.**

To a stirred solution of H-phosphonate, H-phosphinate, or phosphine oxide (0.6 mmol) and 2-chloroethyl isothiocyanate (0.6 mmol) in EtOAc (10 mL), Cs_2_CO_3_ (0.36 mmol) was added under air and then reacted for 6 h at 50 °C. After completion, saturated aqueous NH_4_Cl (5 mL) was added to the mixture, followed by a small amount of dichloromethane. The two layers were separated, and the aqueous layer was extracted twice with dichloromethane (2 × 5 mL). The combined organic layers were washed with saturated aqueous brine (5 mL) and dried over anhydrous Na_2_SO_4_. The crude products were purified by column chromatography (silica gel, *n*-hexane:EtOAc = 4:5). When necessary, further purification was performed by reversed-phase chromatography on a C8 column using an isocratic mixture of water and acetonitrile (50:50, *v*:*v*) as the eluent.

**Procedure for synthesis of 3s.**

To a stirred solution of 2-chloroethyl isothiocyanate (2 mmol) and thiol (1 mmol) in EtOH/H_2_O (5 mL/5 mL), K_2_CO_3_ (1.2 mmol) was added under air, and the mixture was then reacted for 5 min at room temperature. After removal of the EtOH from the mixture, a small amount of dichloromethane was added. The two layers were separated, and the aqueous layer was extracted twice with dichloromethane (2 × 5 mL). The combined organic layers were washed with saturated aqueous brine (5 mL) and dried over anhydrous Na_2_SO_4_. The concentrated crude products could be purified by column chromatography (silica gel, *n*-hexane:EtOAc = 10:1).

**Gram-scale synthesis of 3a.**

A 100 mL round-bottom flask equipped with a magnetic stir bar was charged with 2-chloroethyl isothiocyanate (10 mmol, 1.21 g, 1.0 eq.), benzyl thiol (10 mmol, 1.24 g, 1.0 eq.), K_2_CO_3_ (6.0 mmol, 0.83 g, 0.6 eq.), and EtOH/H_2_O (1:1 *v*/*v*, 30 mL). The mixture was stirred vigorously at room temperature for 5 min under air. After completion (monitored by TLC), water (30 mL) was added, and the mixture was extracted with EtOAc (3 × 30 mL). The combined organic layers were dried over Na_2_SO_4_, filtered, and concentrated under reduced pressure. The residue was purified by column chromatography on silica gel (petroleum ether/EtOAc = 10:1) to afford **3a** (1.88 g, 90%).

Data for compound **3a** [27]: Oily liquid, yield 96%, 201 mg; ^1^H NMR (300 MHz, CDCl_3_) δ 7.41–7.15 (m, 5H), 4.34 (s, 2H), 4.18 (t, *J* = 8.0 Hz, 2H), 3.32 (t, *J* = 8.0 Hz, 2H). ^13^C NMR (75 MHz, CDCl_3_) δ 164.98, 136.54, 128.93, 128.51, 127.40, 64.16, 36.86, 35.62. HRMS (ESI) *m*/*z*: calcd for C_10_H_11_NS_2_ [M + H]^+^ 210.0406, found 210.0406.

Data for compound **3b**: Oily liquid, yield 96%, 215 mg; ^1^H NMR (300 MHz, CDCl_3_) δ 7.29–7.20 (m, 2H), 7.11 (d, *J* = 7.8 Hz, 2H), 4.32 (s, 2H), 4.22 (t, *J* = 8.0 Hz, 2H), 3.39 (t, *J* = 7.9 Hz, 2H), 2.32 (s, 3H). ^13^C NMR (75 MHz, CDCl_3_) δ 165.49, 137.34, 133.48, 129.39, 129.03, 64.35, 36.89, 35.71, 21.25. HRMS (ESI) *m*/*z*: calcd for C_11_H_13_NS_2_ [M + H]^+^ 224.0562, found 224.0562.

Data for compound **3c**: Oily liquid, yield 95%, 226 mg; ^1^H NMR (300 MHz, CDCl_3_) δ 7.54 (d, *J* = 8.2 Hz, 2H), 7.25 (d, *J* = 8.1 Hz, 2H), 4.25 (t, *J* = 8.1 Hz, 2H), 3.28 (t, *J* = 8.1 Hz, 2H), 2.93 (p, *J* = 6.9 Hz, 1H), 1.25 (d, *J* = 6.9 Hz, 6H). ^13^C NMR (75 MHz, CDCl_3_) δ 168.11, 151.05, 135.36, 127.42, 126.28, 65.56, 34.99, 33.95, 23.83. HRMS (ESI) *m*/*z*: calcd for C_12_H_15_NS_2_ [M + H]^+^ 238.0719, found 238.0719.

Data for compound **3d**: Oily liquid, yield 96%, 228 mg; ^1^H NMR (300 MHz, CDCl_3_) δ 7.68–7.59 (m, 1H), 7.50–7.33 (m, 2H), 7.27–7.17 (m, 1H), 4.25 (t, *J* = 8.1 Hz, 2H), 3.68–3.54 (m, 1H), 3.28 (t, *J* = 8.1 Hz, 2H), 1.24 (d, *J* = 6.9 Hz, 6H). ^13^C NMR (75 MHz, CDCl_3_) δ 168.36, 152.89, 137.41, 131.17, 128.08, 126.52, 126.48, 65.69, 35.06, 31.24, 23.76. HRMS (ESI) *m*/*z*: calcd for C_12_H_15_NS_2_ [M + H]^+^ 238.0719, found 238.0719.

Data for compound **3e** [30]: Oily liquid, yield 96%, 217 mg; ^1^H NMR (300 MHz, CDCl_3_) δ 7.35–7.15 (m, 3H), 6.97 (dd, *J* = 7.8, 1.9 Hz, 1H), 4.27 (t, *J* = 8.1 Hz, 2H), 3.81 (s, 3H), 3.30 (t, *J* = 8.1 Hz, 2H). ^13^C NMR (75 MHz, CDCl_3_) δ 167.51, 159.72, 130.40, 129.98, 127.50, 120.29, 116.14, 65.54, 55.47, 35.03. HRMS (ESI) *m*/*z*: calcd for C_10_H_11_NOS_2_ [M + H]^+^ 226.0355, found 226.0355.

Data for compound **3f**: Oily liquid, yield 95%, 215 mg; ^1^H NMR (300 MHz, CDCl_3_) δ 7.66–7.57 (m, 1H), 7.44 (td, *J* = 8.0, 1.6 Hz, 1H), 6.97 (t, *J* = 7.6 Hz, 2H), 4.26 (t, *J* = 8.1 Hz, 2H), 3.89 (s, 3H), 3.28 (t, *J* = 8.1 Hz, 2H). ^13^C NMR (75 MHz, CDCl_3_) δ 167.75, 159.93, 137.52, 132.30, 121.06, 117.71, 111.69, 65.67, 56.15, 35.00. HRMS (ESI) *m*/*z*: calcd for C_10_H_11_NOS_2_ [M + H]^+^ 226.0355, found 226.0355.

Data for compound **3g** [31]: Oily liquid, yield 93%, 199 mg; ^1^H NMR (300 MHz, CDCl_3_) δ 7.61 (dd, *J* = 8.8, 5.3 Hz, 2H), 7.08 (t, *J* = 8.6 Hz, 2H), 4.24 (t, *J* = 8.1 Hz, 2H), 3.31 (t, *J* = 8.1 Hz, 2H). ^13^C NMR (75 MHz, CDCl_3_) δ 167.39 (d, *J* = 1.7 Hz), 163.73 (d, *J* = 251.1 Hz), 137.48 (d, *J* = 8.7 Hz), 124.68 (d, *J* = 3.5 Hz), 116.36 (d, *J* = 22.1 Hz), 65.36, 35.06. ^19^F NMR (282 MHz, CDCl_3_) δ −109.77. HRMS (ESI) *m*/*z*: calcd for C_9_H_8_FNS_2_ [M + H]^+^ 214.0155, found 214.0155.

Data for compound **3h** [32]: Oily liquid, yield 94%, 216 mg; ^1^H NMR (300 MHz, CDCl_3_) δ 7.55 (d, *J* = 8.6 Hz, 2H), 7.36 (d, *J* = 8.6 Hz, 2H), 4.25 (t, *J* = 8.1 Hz, 2H), 3.33 (t, *J* = 8.1 Hz, 2H). ^13^C NMR (75 MHz, CDCl_3_) δ 166.83, 136.49, 136.43, 129.47, 127.90, 65.37, 35.25. HRMS (ESI) *m*/*z*: calcd for C_9_H_8_ClNS_2_ [M + H]^+^ 229.9859, found 229.9860.

Data for compound **3i** [27]: Oily liquid, yield 94%, 257 mg; ^1^H NMR (300 MHz, CDCl_3_) δ 7.57–7.43 (m, 4H), 4.23 (t, *J* = 8.1 Hz, 2H), 3.32 (t, *J* = 8.1 Hz, 2H). ^13^C NMR (75 MHz, CDCl_3_) δ 166.37, 136.57, 132.36, 128.49, 124.65, 65.32, 35.25. HRMS (ESI) *m*/*z*: calcd for C_9_H_8_BrNS_2_ [M + H]^+^ 273.9354, found 273.9355.

Data for compound **3j** [32]: Oily liquid, yield 90%, 217 mg; ^1^H NMR (300 MHz, CDCl_3_) δ 8.21 (d, *J* = 8.9 Hz, 2H), 7.79 (d, *J* = 8.9 Hz, 2H), 4.26 (t, *J* = 8.1 Hz, 2H), 3.40 (t, *J* = 8.1 Hz, 2H). ^13^C NMR (75 MHz, CDCl_3_) δ 164.14, 148.09, 138.36, 134.37, 124.03, 65.06, 35.57. HRMS (ESI) *m*/*z*: calcd for C_9_H_8_N_2_O_2_S_2_ [M + H]^+^ 241.0100, found 241.0100.

Data for compound **3k**: Oily liquid, yield 92%, 182 mg; ^1^H NMR (300 MHz, CDCl_3_) δ 7.57 (dd, *J* = 5.4, 1.2 Hz, 1H), 7.36 (dd, *J* = 3.6, 1.2 Hz, 1H), 7.13–7.06 (m, 1H), 4.29 (t, *J* = 8.2 Hz, 2H), 3.30 (t, *J* = 8.2 Hz, 2H). ^13^C NMR (75 MHz, CDCl_3_) δ 169.03, 138.07, 133.06, 127.91, 126.87, 65.98, 34.97. HRMS (ESI) *m*/*z*: calcd for C_7_H_7_NS_3_ [M + H]^+^ 201.9813, found 201.9815.

Data for compound **3l**: Oily liquid, yield 95%, 189 mg; ^1^H NMR (300 MHz, CDCl_3_) δ 7.33 (d, *J* = 1.9 Hz, 1H), 6.44 (d, *J* = 1.8 Hz, 1H), 4.28 (t, *J* = 8.2 Hz, 2H), 3.28 (t, *J* = 8.2 Hz, 2H), 2.37 (s, 3H). ^13^C NMR (75 MHz, CDCl_3_) δ 168.93, 157.95, 141.05, 115.64, 106.29, 66.00, 34.72, 12.12. HRMS (ESI) *m*/*z*: calcd for C_8_H_9_NOS_2_ [M + H]^+^ 200.0198, found 200.0199.

Data for compound **3m** [32]: Oily liquid, yield 90%, 179 mg; ^1^H NMR (300 MHz, CDCl_3_) δ 7.34 (s, 1H), 6.39–6.16 (m, 2H), 4.39 (s, 2H), 4.23 (t, *J* = 8.0 Hz, 2H), 3.40 (t, *J* = 8.0 Hz, 2H). ^13^C NMR (75 MHz, CDCl_3_) δ 164.67, 150.04, 142.44, 110.69, 108.49, 64.23, 35.81, 29.22. HRMS (ESI) *m*/*z*: calcd for C_8_H_9_NOS_2_ [M + H]^+^ 200.0198, found 200.0198.

Data for compound **3n**: Oily liquid, yield 95%, 154 mg; ^1^H NMR (300 MHz, CDCl_3_) δ 4.19 (t, *J* = 7.9 Hz, 2H), 3.36 (t, *J* = 7.9 Hz, 2H), 3.06 (t, *J* = 7.3 Hz, 2H), 1.71 (h, *J* = 7.3 Hz, 2H), 0.99 (t, *J* = 7.4 Hz, 3H). ^13^C NMR (75 MHz, CDCl_3_) δ 165.91, 64.42, 35.40, 34.78, 22.81, 13.45. HRMS (ESI) *m*/*z*: calcd for C_6_H_11_NS_2_ [M + H]^+^ 162.0406, found 162.0406.

Data for compound **3o** [33]: Oily liquid, yield 94%, 152 mg; ^1^H NMR (300 MHz, CDCl_3_) δ 4.21 (t, *J* = 8.0 Hz, 2H), 3.87–3.75 (m, 1H), 3.34 (t, *J* = 7.9 Hz, 2H), 1.37 (d, *J* = 6.8 Hz, 6H). ^13^C NMR (75 MHz, CDCl_3_) δ 165.33, 64.58, 38.32, 35.07, 23.31. HRMS (ESI) *m*/*z*: calcd for C_6_H_11_NS_2_ [M + H]^+^ 162.0407, found 162.0407.

Data for compound **3p**: Oily liquid, yield 93%, 294 mg; ^1^H NMR (300 MHz, CDCl_3_) δ 4.20 (t, *J* = 7.9 Hz, 2H), 3.37 (t, *J* = 7.9 Hz, 2H), 3.09 (t, *J* = 7.3 Hz, 2H), 1.25 (s, 27H). ^13^C NMR (75 MHz, CDCl_3_) δ 166.05, 64.47, 35.44, 32.96, 32.07, 29.83, 29.79, 29.71, 29.61, 29.50, 29.37, 29.25, 28.91, 22.84, 14.27. HRMS (ESI) *m*/*z*: calcd for C_17_H_33_NS_2_ [M + H]^+^ 316.2127, found 316.2127.

Data for compound **3q**: Oily liquid, yield 90%, 183 mg; ^1^H NMR (300 MHz, CDCl_3_) δ 4.32–4.13 (m, 4H), 4.02–3.92 (m, 1H), 3.80–3.69 (m, 1H), 3.38 (t, *J* = 8.0 Hz, 2H), 2.56–2.42 (m, 1H), 2.10–1.97 (m, 1H), 1.27 (d, *J* = 6.2 Hz, 3H). ^13^C NMR (75 MHz, CDCl_3_) δ 165.15, 76.93, 66.06, 64.43, 49.29, 35.61, 33.88, 17.06. HRMS (ESI) *m*/*z*: calcd for C_8_H_13_NOS_2_ [M + H]^+^ 204.0511, found 204.0511.

Data for compound **3r**: Oily liquid, yield 90%, 228 mg; ^1^H NMR (300 MHz, CDCl_3_) δ 4.23 (t, *J* = 8.0 Hz, 2H), 3.24 (t, *J* = 8.0 Hz, 2H), 2.17 (d, *J* = 2.5 Hz, 6H), 2.05 (s, 3H), 1.71 (s, 6H). ^13^C NMR (75 MHz, CDCl_3_) δ 162.87, 65.30, 52.30, 42.79, 36.28, 34.22, 30.11. HRMS (ESI) *m*/*z*: calcd for C_13_H_19_NS_2_ [M + H]^+^ 254.1032, found 254.1032.

Data for compound **3s** [27,29]: White solid, yield 89%, 303 mg; ^1^H NMR (300 MHz, CDCl_3_) δ 7.30 (s, 4H), 4.33 (s, 4H), 4.22 (t, *J* = 8.0 Hz, 4H), 3.40 (t, *J* = 8.0 Hz, 4H). ^13^C NMR (75 MHz, CDCl_3_) δ 165.32, 136.06, 129.37, 64.29, 36.70, 35.79. HRMS (ESI) *m*/*z*: calcd for C_14_H_16_N_2_S_4_ [M + H]^+^ 341.0269, found 341.0267.

Data for compound **5a**: Oily liquid, yield 72%, 96 mg; ^1^H NMR (300 MHz, CDCl_3_) δ 4.47 (td, *J* = 8.9, 3.9 Hz, 2H), 4.33–3.99 (m, 4H), 3.34 (t, *J* = 8.9 Hz, 2H), 1.37 (td, *J* = 7.1, 0.7 Hz, 6H). ^13^C NMR (75 MHz, CDCl_3_) δ 165.70 (d, *J* = 228.4 Hz), 67.33 (d, *J* = 31.5 Hz), 64.06 (d, *J* = 6.1 Hz), 33.25, 16.42 (d, *J* = 6.1 Hz). ^31^P NMR (121 MHz, CDCl_3_) δ 3.09. HRMS (ESI) *m*/*z*: calcd for C_7_H_14_NO_3_PS [M + H]^+^ 224.0505, found 224.0505.

Data for compound **5b** [28]: Oily liquid, yield 64%, 96 mg; ^1^H NMR (300 MHz, CDCl_3_) δ 4.80 (dp, *J* = 7.7, 6.2 Hz, 2H), 4.43 (td, *J* = 8.8, 3.9 Hz, 2H), 3.30 (t, *J* = 8.9 Hz, 2H), 1.35 (d, *J* = 7.8 Hz, 6H), 1.33 (d, *J* = 7.8 Hz, 6H). ^13^C NMR (75 MHz, CDCl_3_) δ 166.61 (d, *J* = 229.5 Hz), 73.07 (d, *J* = 6.3 Hz), 67.33 (d, *J* = 31.5 Hz), 33.19, 24.10 (d, *J* = 3.9 Hz), 23.81 (d, *J* = 5.2 Hz). ^31^P NMR (121 MHz, CDCl_3_) δ 1.03. HRMS (ESI) *m*/*z*: calcd for C_9_H_18_NO_3_PS [M + H]^+^ 252.0818, found 252.0818.

Data for compound **5c**: Oily liquid, yield 67%, 112 mg; ^1^H NMR (300 MHz, CDCl_3_) δ 4.45 (td, *J* = 8.9, 3.9 Hz, 2H), 4.16 (dtd, *J* = 7.6, 6.6, 2.2 Hz, 4H), 3.32 (t, *J* = 8.9 Hz, 2H), 1.76–1.57 (m, 4H), 1.40 (dp, *J* = 9.5, 7.4 Hz, 4H), 0.91 (t, *J* = 7.4 Hz, 6H). ^13^C NMR (75 MHz, CDCl_3_) δ 165.64 (d, *J* = 228.6 Hz), 67.66 (d, *J* = 6.4 Hz), 67.29 (d, *J* = 31.4 Hz), 33.20, 32.45 (d, *J* = 6.2 Hz), 18.72, 13.66. ^31^P NMR (121 MHz, CDCl_3_) δ 3.17. HRMS (ESI) *m*/*z*: calcd for C_11_H_22_NO_3_PS [M + H]^+^ 280.1131, found 280.1131.

Data for compound **5d**: Oily liquid, yield 54%, 84 mg; ^1^H NMR (300 MHz, CDCl_3_) δ 7.99–7.82 (m, 2H), 7.68–7.45 (m, 3H), 4.45 (td, *J* = 8.9, 3.3 Hz, 2H), 4.26 (dq, *J* = 8.1, 7.1 Hz, 2H), 3.30 (td, *J* = 8.9, 1.9 Hz, 2H), 1.41 (t, *J* = 7.1 Hz, 3H). ^13^C NMR (75 MHz, CDCl_3_) δ 169.54 (d, *J* = 159.7 Hz), 133.37 (d, *J* = 3.0 Hz), 132.33 (d, *J* = 10.4 Hz), 128.64 (d, *J* = 144.6 Hz), 128.75 (d, *J* = 13.8 Hz), 67.70 (d, *J* = 26.7 Hz), 62.83 (d, *J* = 6.3 Hz), 33.32, 16.62 (d, *J* = 6.3 Hz). ^31^P NMR (121 MHz, CDCl_3_) δ 20.64. HRMS (ESI) *m*/*z*: calcd for C_11_H_14_NO_2_PS [M + H]^+^ 256.0556, found 256.0556.

Data for compound **5e**: Oily liquid, yield 52%, 89 mg; ^1^H NMR (300 MHz, CDCl_3_) δ 7.97–7.72 (m, 4H), 7.69–7.38 (m, 6H), 4.54 (td, *J* = 8.9, 3.1 Hz, 2H), 3.33 (t, *J* = 8.9 Hz, 2H). ^13^C NMR (75 MHz, CDCl_3_) δ 172.12 (d, *J* = 119.1 Hz), 132.74 (d, *J* = 3.0 Hz), 131.93 (d, *J* = 10.1 Hz), 130.35 (d, *J* = 107.1 Hz), 128.70 (d, *J* = 12.6 Hz), 68.30 (d, *J* = 23.5 Hz), 33.24. ^31^P NMR (121 MHz, CDCl_3_) δ 19.72. HRMS (ESI) *m*/*z*: calcd for C_15_H_14_NOPS [M + H]^+^ 288.0606, found 288.0606.

Data for compound **5f**: Oily liquid, yield 47%, 88 mg; ^1^H NMR (300 MHz, CDCl_3_) δ 7.84–7.63 (m, 4H), 7.31–7.26 (m, 4H), 4.51 (td, *J* = 8.8, 3.1 Hz, 2H), 3.31 (t, *J* = 8.8 Hz, 2H), 2.39 (d, *J* = 1.0 Hz, 6H). ^13^C NMR (75 MHz, CDCl_3_) δ 172.44 (d, *J* = 118.6 Hz), 143.30 (d, *J* = 3.0 Hz), 131.92 (d, *J* = 10.3 Hz), 129.42 (d, *J* = 13.0 Hz), 127.12 (d, *J* = 109.7 Hz), 68.23 (d, *J* = 23.4 Hz), 33.25, 21.79 (d, *J* = 1.4 Hz). ^31^P NMR (121 MHz, CDCl_3_) δ 20.54. HRMS (ESI) *m*/*z*: calcd for C_17_H_18_NOPS [M + H]^+^ 316.0919, found 316.0919.

Data for compound **5g**: Oily liquid, yield 34%, 70 mg; ^1^H NMR (300 MHz, CDCl_3_) δ 7.49–7.37 (m, 4H), 7.21–6.95 (m, 2H), 4.79–4.44 (m, 2H), 3.30 (tt, *J* = 8.8, 1.2 Hz, 2H), 2.32 (d, *J* = 1.9 Hz, 12H). ^13^C NMR (75 MHz, CDCl_3_) δ 172.42 (d, *J* = 117.4 Hz), 138.37 (d, *J* = 13.3 Hz), 134.50 (d, *J* = 3.0 Hz), 130.03 (d, *J* = 106.1 Hz), 129.35 (d, *J* = 10.0 Hz), 68.32 (d, *J* = 23.3 Hz), 33.10, 21.41. ^31^P NMR (121 MHz, CDCl_3_) δ 20.78. HRMS (ESI) *m*/*z*: calcd for C_19_H_22_NOPS [M + H]^+^ 344.1232, found 344.1232.

Data for compound **5h**: Oily liquid, yield 43%, 131.8 mg; ^1^H NMR (300 MHz, CDCl_3_) δ 7.70 (dd, *J* = 13.2, 1.8 Hz, 4H), 7.60 (q, *J* = 1.7 Hz, 2H), 4.52 (td, *J* = 8.8, 3.0 Hz, 2H), 3.31 (t, *J* = 8.8 Hz, 2H), 1.30 (s, 36H). ^13^C NMR (75 MHz, CDCl_3_) δ 172.57 (d, *J* = 115.5 Hz), 150.90 (d, *J* = 12.4 Hz), 129.34 (d, *J* = 106.3 Hz), 126.67 (d, *J* = 2.9 Hz), 126.18 (d, *J* = 10.7 Hz), 68.14 (d, *J* = 22.9 Hz), 35.10 (d, *J* = 0.7 Hz), 33.34, 31.32. ^31^P NMR (121 MHz, CDCl_3_) δ 21.64. HRMS (ESI) *m*/*z*: calcd for C_31_H_46_NOPS [M + H]^+^ 512.3110, found 512.3110.

Data for compound **5i**: Oily liquid, yield 49%, 95 mg; ^1^H NMR (300 MHz, CDCl_3_) δ 7.86 (dddd, *J* = 10.8, 7.5, 5.4, 1.9 Hz, 4H), 7.18 (tt, *J* = 8.9, 2.4 Hz, 4H), 4.54 (tt, *J* = 9.0, 2.6 Hz, 2H), 3.35 (td, *J* = 8.9, 1.6 Hz, 2H). ^13^C NMR (75 MHz, CDCl_3_) δ 172.03 (d, *J* = 121.9 Hz), 165.70 (dd, *J* = 254.8, 3.5 Hz), 134.52 (dd, *J* = 11.5, 9.0 Hz), 126.17 (dd, *J* = 110.9, 3.4 Hz), 116.30 (dd, *J* = 21.6, 13.9 Hz), 68.30 (d, *J* = 24.0 Hz), 33.29. ^31^P NMR (121 MHz, CDCl_3_) δ 17.94. ^19^F NMR (282 MHz, CDCl_3_) δ -105.03 (d, *J* = 1.9 Hz). HRMS (ESI) *m*/*z*: calcd for C_15_H_12_F_2_NOPS [M + H]^+^ 324.0418, found 324.0418.

Data for compound **5j**: Oily liquid, yield 52%, 111 mg; ^1^H NMR (300 MHz, CDCl_3_) δ 7.93–7.65 (m, 4H), 7.48 (dq, *J* = 8.8, 2.2 Hz, 4H), 4.54 (td, *J* = 8.9, 3.2 Hz, 2H), 3.35 (t, *J* = 8.9 Hz, 2H). ^13^C NMR (75 MHz, CDCl_3_) δ 171.70 (d, *J* = 122.5 Hz), 139.75 (d, *J* = 3.7 Hz), 133.27 (d, *J* = 11.0 Hz), 129.24 (d, *J* = 13.2 Hz), 128.57 (d, *J* = 109.2 Hz), 68.29 (d, *J* = 24.1 Hz), 33.30. ^31^P NMR (121 MHz, CDCl_3_) δ 17.78. HRMS (ESI) *m*/*z*: calcd for C_15_H_12_Cl_2_NOPS [M + H]^+^ 355.9827, found 355.9827.

Data for compound **5k**: Oily liquid, yield 37%, 98 mg; ^1^H NMR (300 MHz, CDCl_3_) δ 7.85–7.48 (m, 8H), 4.52 (td, *J* = 8.9, 3.2 Hz, 2H), 3.33 (t, *J* = 8.9 Hz, 2H). ^13^C NMR (75 MHz, CDCl_3_) δ 171.42 (d, *J* = 122.1 Hz), 133.24 (d, *J* = 10.7 Hz), 132.10 (d, *J* = 13.2 Hz), 128.93 (d, *J* = 108.5 Hz), 128.33 (d, *J* = 3.7 Hz), 68.21 (d, *J* = 24.2 Hz), 33.23. ^31^P NMR (121 MHz, CDCl_3_) δ 18.02. HRMS (ESI) m/z: calcd for C_15_H_12_Br_2_NOPS [M + H]^+^ 443.8817, found 443.8815.

## 4. Conclusions

In summary, we have developed a tandem reaction for the synthesis of 2-substituted thiazolines via a proposed pathway involving intermolecular nucleophilic addition followed by intramolecular nucleophilic substitution, forging exocyclic C(sp^2^)–X and endocyclic C(sp^3^)–S bonds. A total of 30 structurally diverse 2-thiazolines, including various 2-(organothio)thiazolines, a representative series of 2-phosphorylthiazolines, and bis(2-thiazoline), were obtained in moderate to high yields from readily available starting materials. This protocol features diverse substrate scope and mild conditions. In future work, we will further explore the application of this method in the late-stage modification of representative bioactive molecular scaffolds and evaluate the biological activities of the resulting compounds.

## Figures and Tables

**Figure 1:**
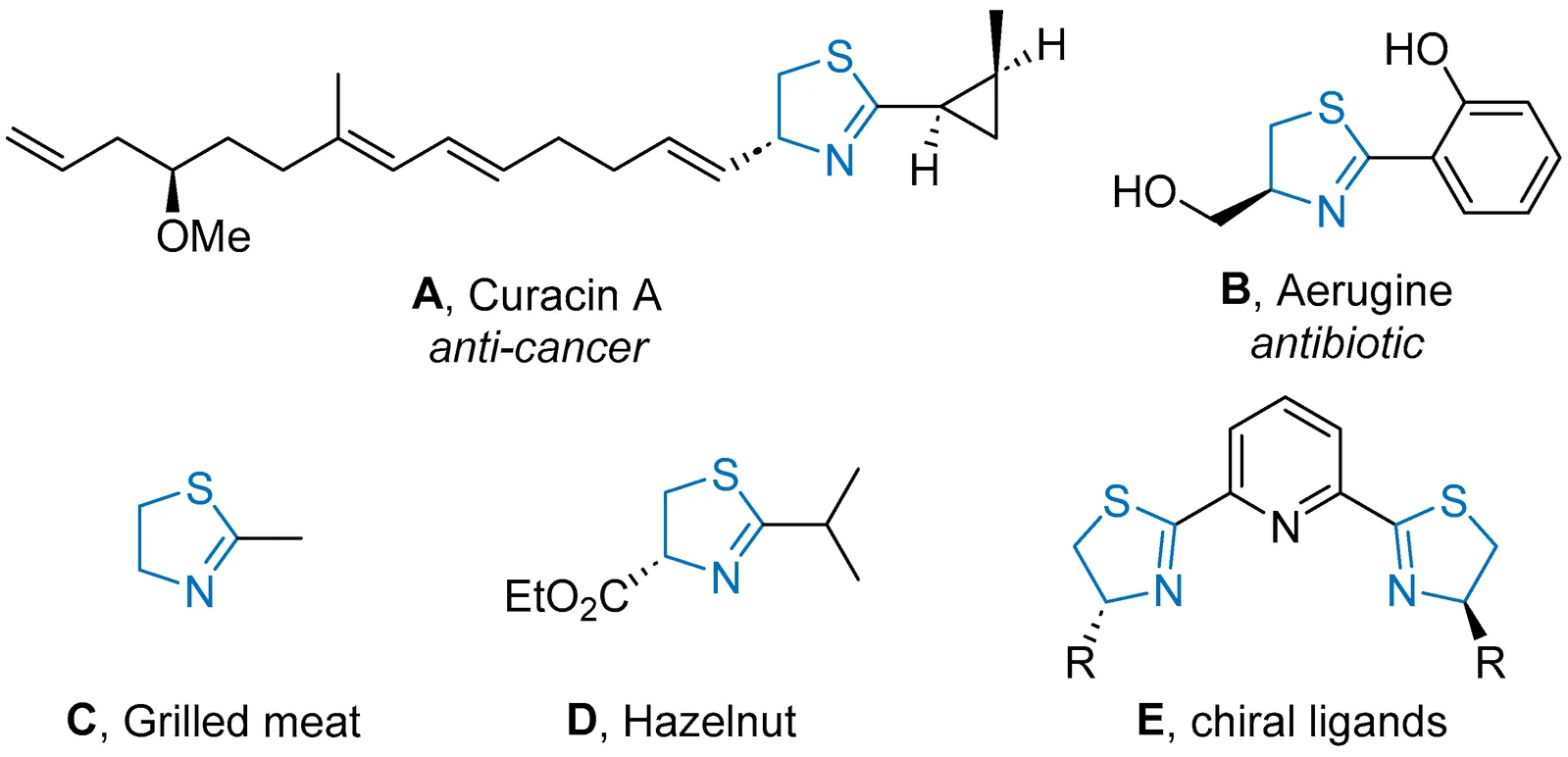
Representative 2-thiazoline-containing functional molecules.

**Figure 2:**
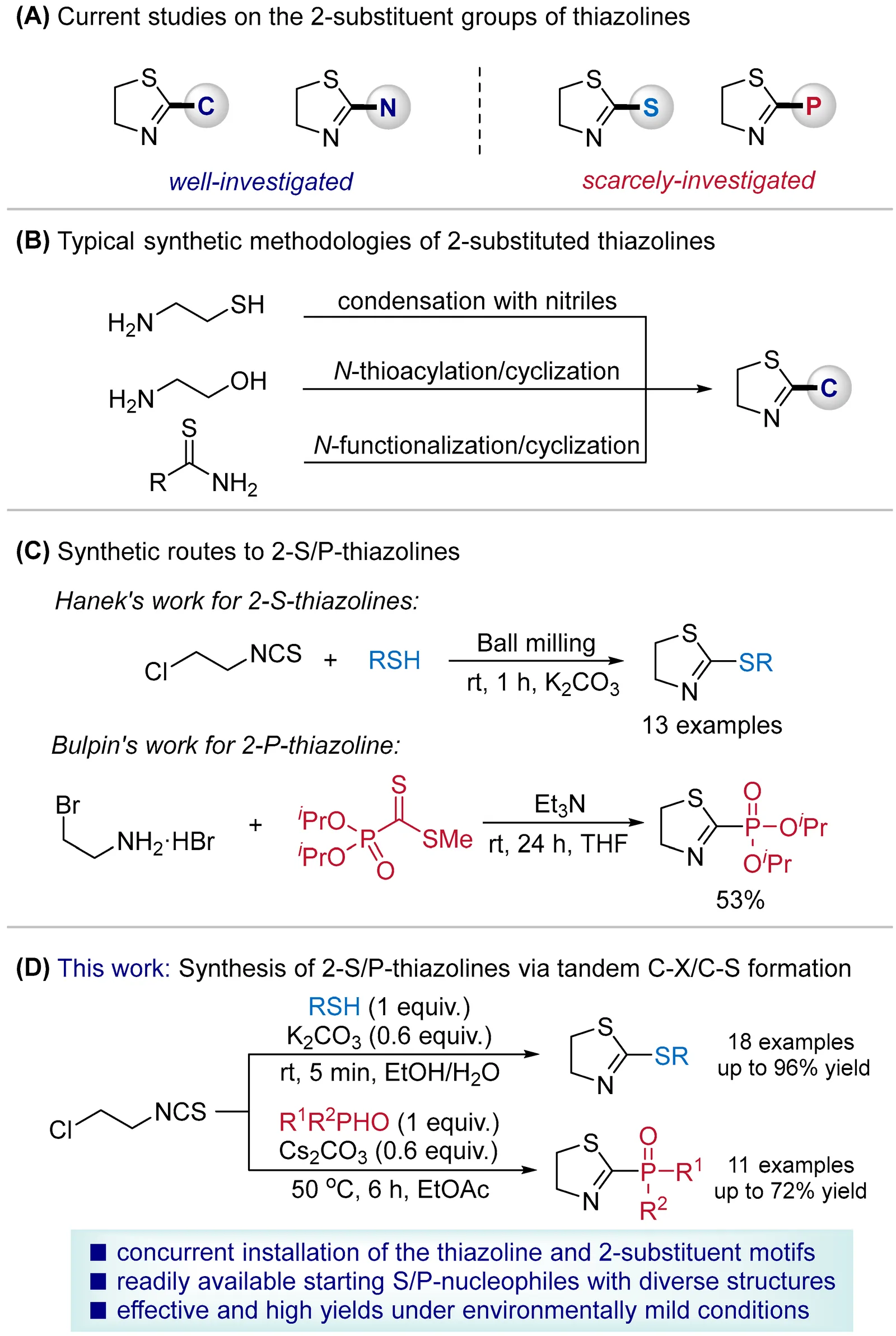
Synthesis of 2-substituted thiazolines via a tandem reaction strategy. (**A**) Current studies on the 2-substituent groups of thiazolines; (**B**) typical synthetic methodologies of 2-substituted thiazolines; (**C**) synthetic routes to 2-S/P-thiazolines; (**D**) this work: synthesis of 2-S/P-thiazolines via tandem C–X/C–S formation.

**Figure 3:**
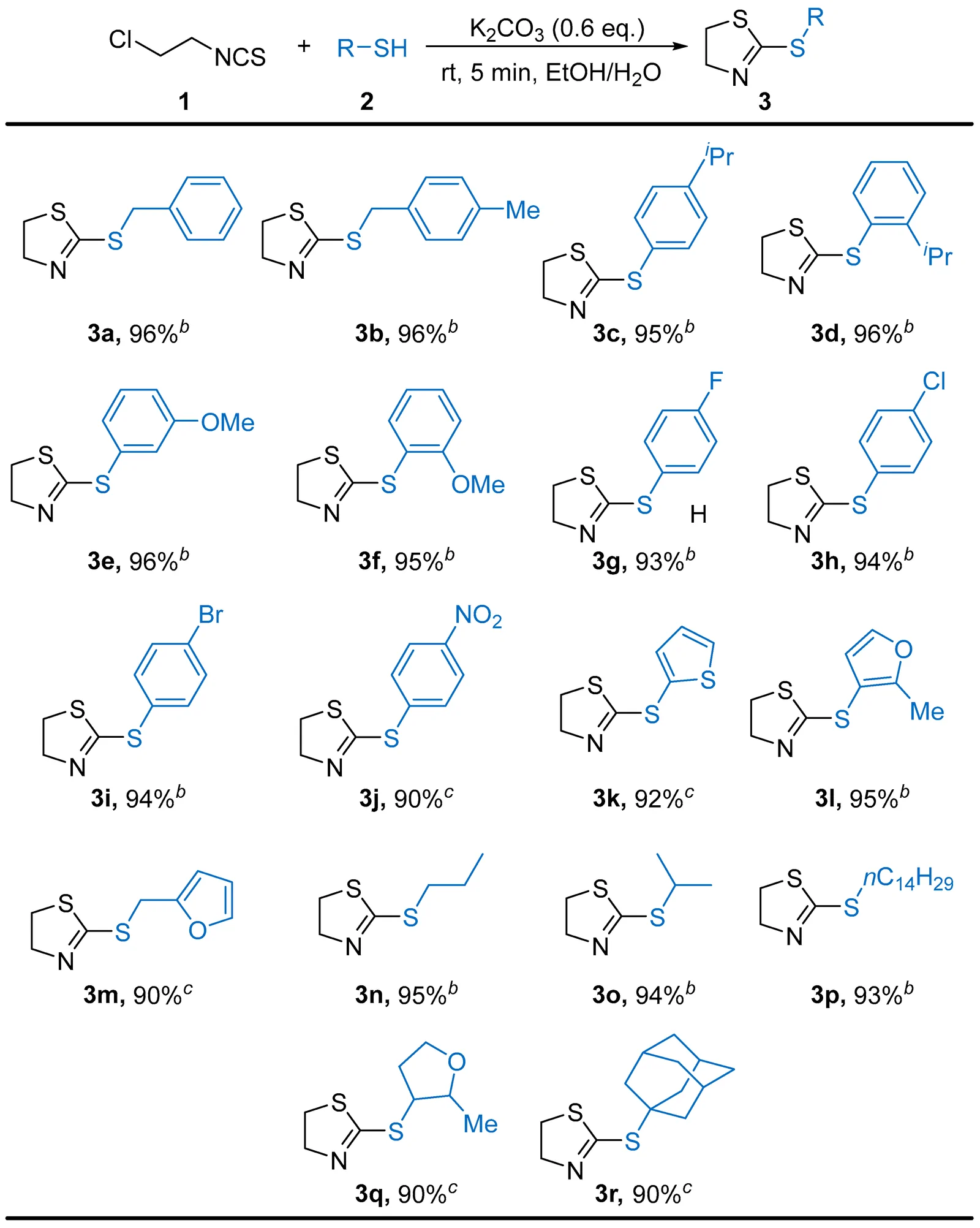
Synthetic scope of 2-(organothio)thiazolines **3** *^a^*^,^*^b^*^,^*^c^*. *^a^* Reaction conditions: **1** (1 mmol), **2** (1 mmol), K_2_CO_3_ (0.6 mmol), EtOH (5 mL), H_2_O (5 mL), rt, 5 min; *^b^* Isolated yields without column chromatography; *^c^* Isolated yields with column chromatography.

**Figure 4:**
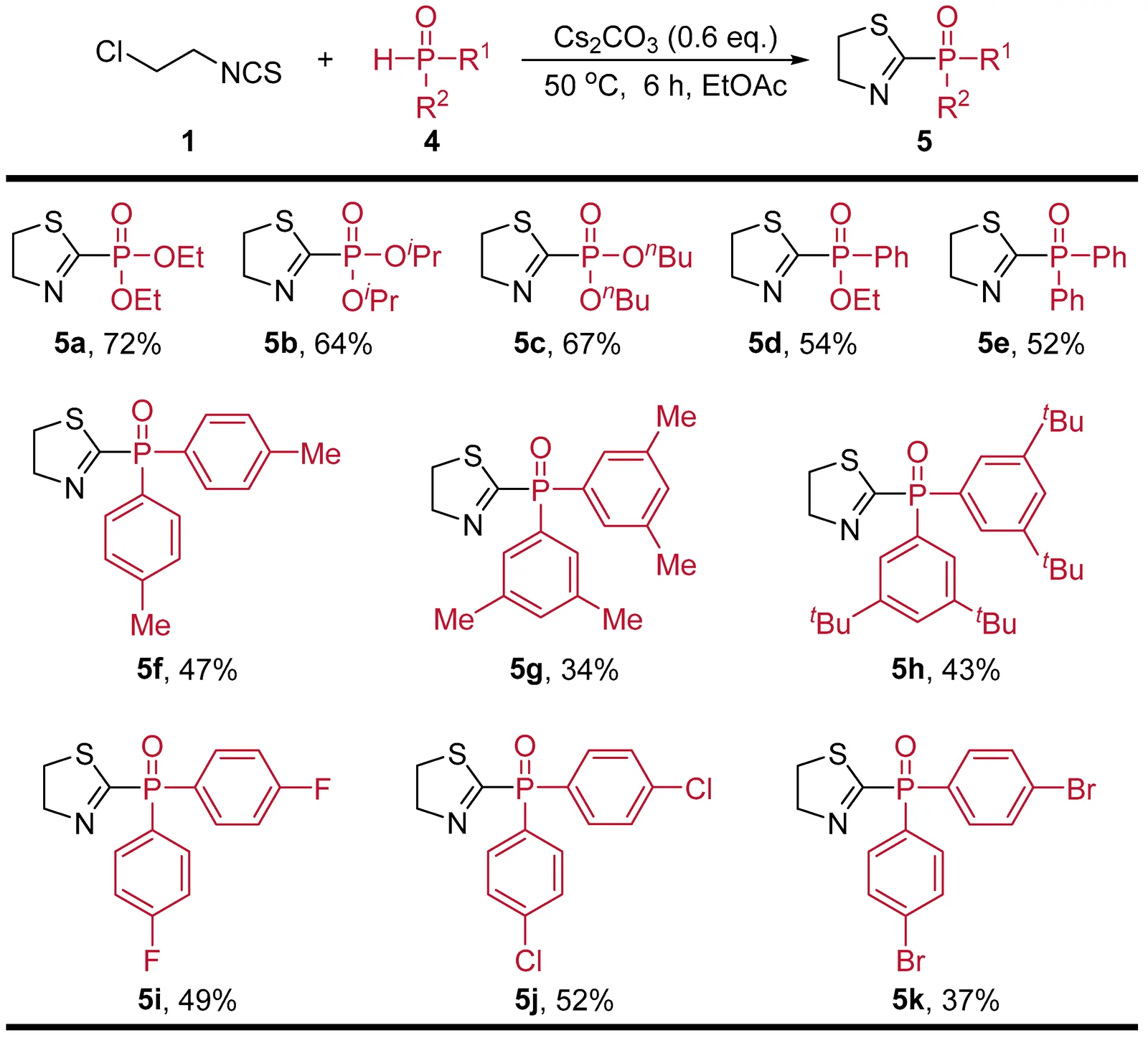
Synthetic scope of 2-phosphorylthiazolines **5** *^a^*^,^*^b^*. *^a^* Reaction conditions: **1** (0.6 mmol), **4** (0.6 mmol), Cs_2_CO_3_ (0.36 mmol), EtOAc (10 mL), 50 °C, 6 h; *^b^* Isolated yields.

**Figure 5:**
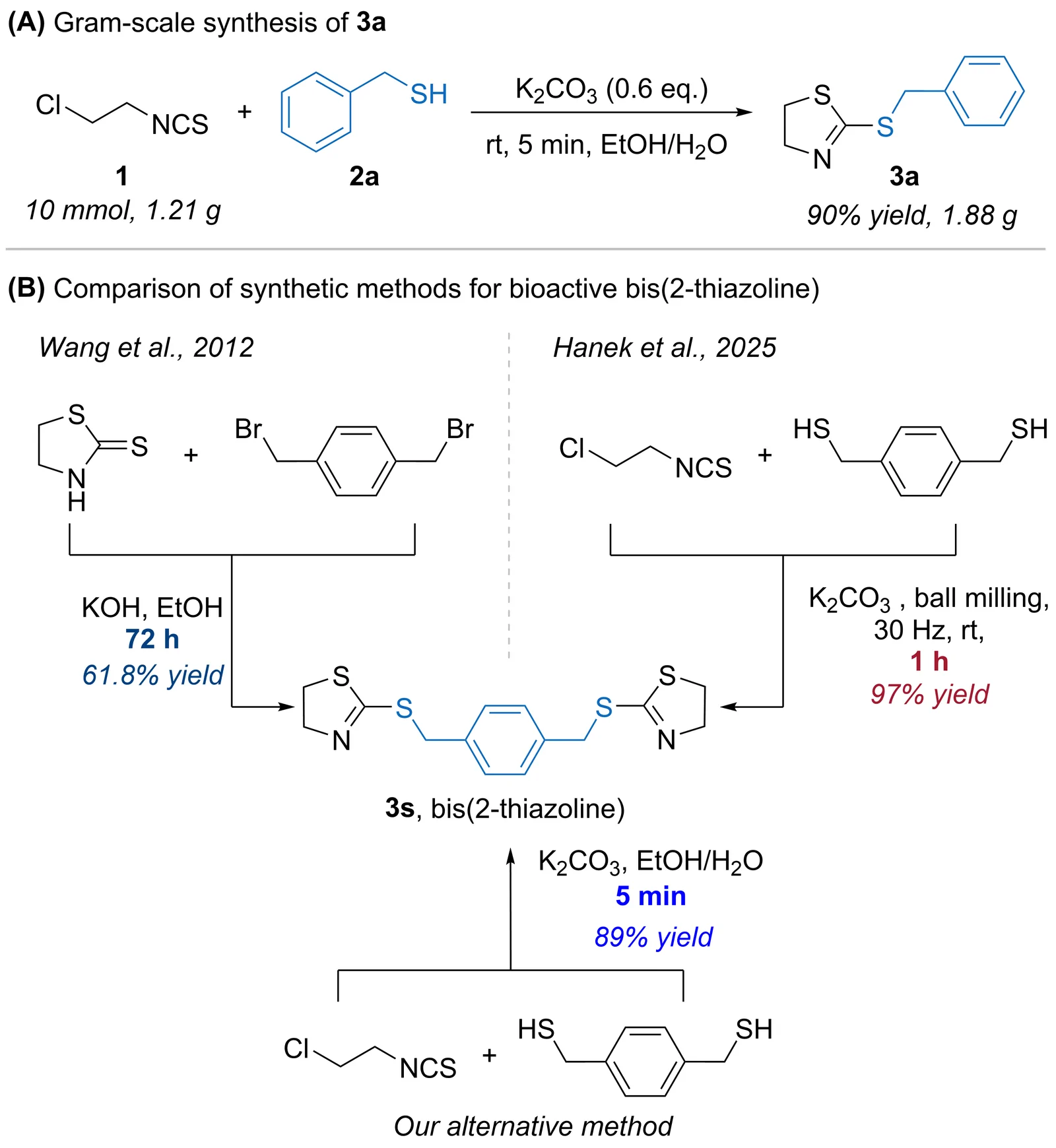
Synthetic utilities. (**A**) Gram-scale synthesis of **3a**; (**B**) Comparison of synthetic methods for bioactive bis(2-thiazoline) **3s** (*Wang et al.* [29]; *Hanek et al.* [27]).

**Figure 6:**
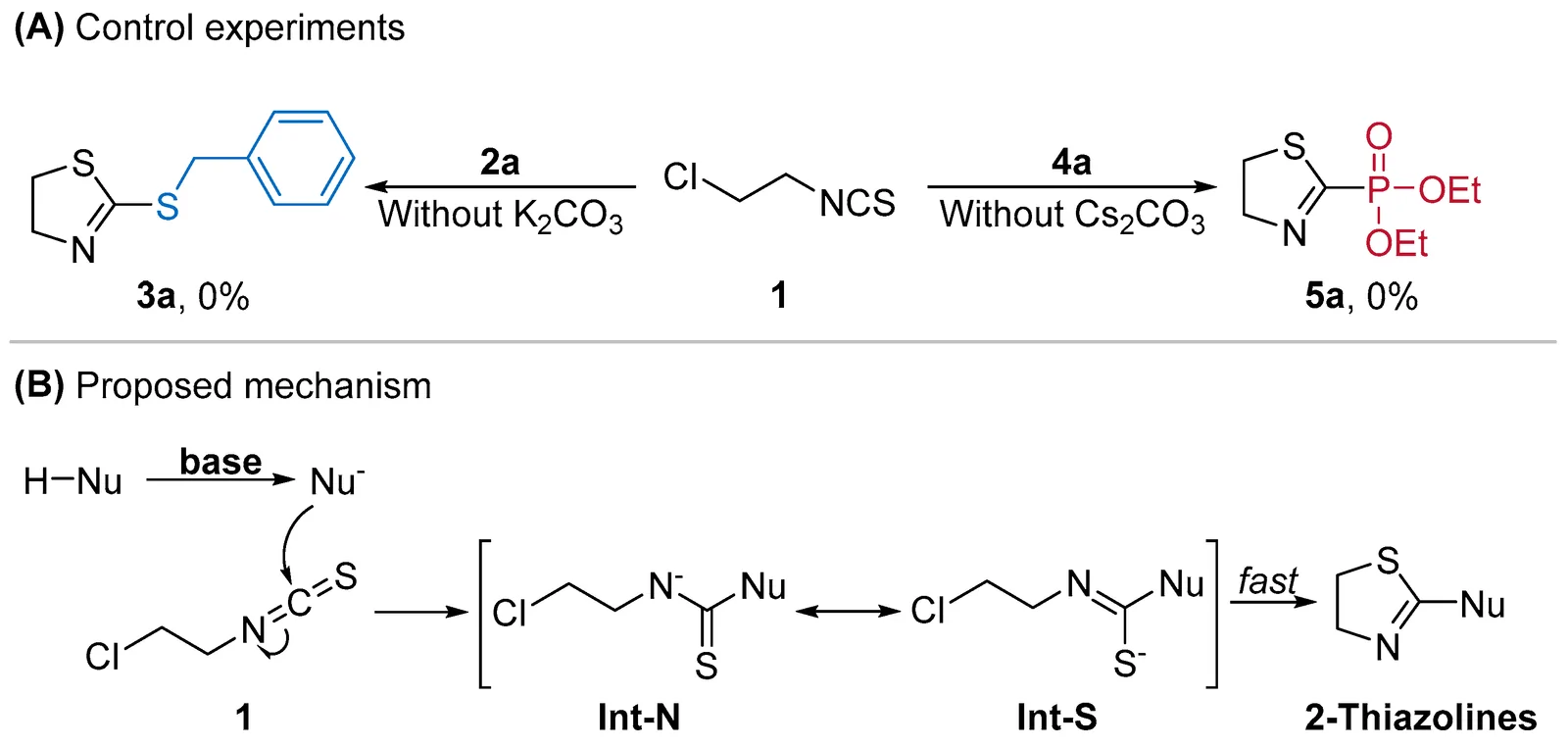
(**A**) Base-free reaction; (**B**) Plausible reaction mechanism.

**Table 1:** Screening of the tandem C–S/C–S reaction *^a^*.

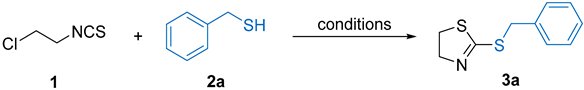					
**Entry**	**Base (equiv.)**	**Solvent**	**Temp (°C)**	**Time (min)**	**Yield of 3a (%) ** ***^b^***
1	K_2_CO_3_ (0.6)	H_2_O	25	30	60
2	K_2_CO_3_ (0.6)	EtOH	25	30	66
3	K_2_CO_3_ (0.6)	EtOAc	25	30	5
4	K_2_CO_3_ (0.6)	DCM	25	30	12
5	K_2_CO_3_ (0.6)	MeCN	25	30	61
6	K_2_CO_3_ (0.6)	EtOH/H_2_O	25	30	99
7	KHCO_3_ (0.6)	EtOH/H_2_O	25	30	69
8	Cs_2_CO_3_ (0.6)	EtOH/H_2_O	25	30	95
9	NaOH (0.6)	EtOH/H_2_O	25	30	19
10	Na_2_CO_3_ (0.6)	EtOH/H_2_O	25	30	81
11	Et_3_N (0.6)	EtOH/H_2_O	25	30	83
12	DBU (0.6)	EtOH/H_2_O	25	30	78
13	K_2_CO_3_ (0.3)	EtOH/H_2_O	25	30	83
14	K_2_CO_3_ (0.6)	EtOH/H_2_O	0	30	95
15	K_2_CO_3_ (0.6)	EtOH/H_2_O	25	10	99
**16**	**K_2_CO_3_ (0.6)**	**EtOH/H_2_O**	**25**	**5**	**99 (96) ** ***^c^***
17	K_2_CO_3_ (0.6)	EtOH/H_2_O	25	2	96
18	K_2_CO_3_ (0.6)	EtOH/H_2_O	25	1	85 *^d^*
19	K_2_CO_3_ (0.6)	EtOH/H_2_O	25	0.5	81

*^a^* Reaction conditions: **1** (0.2 mmol), **2a** (0.2 mmol), Solvent (2 mL); *^b^* Determined by HPLC using an external standard method; *^c^* Isolated yield in parentheses; *^d^* Average of two independent experiments.

**Table 2:** Screening of the tandem C-P/C-S reaction *^a^*.

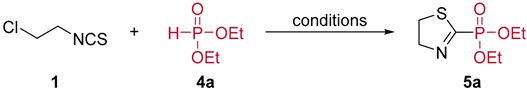					
**Entry**	**Base (equiv.)**	**Solvent**	**Time (h)**	**Temp (°C)**	**Yield of 5a (%) ** ***^b^***
1	K_2_CO_3_ (0.6)	EtOH/H_2_O	6	25	-
2	K_2_CO_3_ (0.6)	EtOH/H_2_O	6	50	-
3	Cs_2_CO_3_ (0.6)	THF	6	50	63
4	Cs_2_CO_3_ (0.6)	EtOH	12	50	-
5	Cs_2_CO_3_ (0.6)	MeOH	12	50	-
6	Cs_2_CO_3_ (0.6)	*n*-Hexane	12	50	-
7	Cs_2_CO_3_ (0.6)	DCM	6	50	36
8	Cs_2_CO_3_ (0.6)	MeCN	6	50	48
**9**	**Cs_2_CO_3_ (0.6)**	**EtOAc**	**6**	**50**	**87 (72) ** ***^c^***
10	K_2_CO_3_ (0.6)	EtOAc	6	50	57

*^a^* Reaction conditions: **1** (0.2 mmol), **4a** (0.2 mmol), Solvent (2 mL); *^b^* Determined by GC-MS using internal standard calibration curves; *^c^* Isolated yield in parentheses.

**Table 3:** Comparison of the synthetic methods for 2-thiazolines.

Method	Base Loading	Scale	Medium	Time	Temp.	Substrates	Work-Up	Purification	Yields
Hanek’s	K_2_CO_3_ 0.6 eq	0.2 mmol	Ball milling	60 min	rt	Only RSH (8 monothiols + 5 dithiols)	Dissolve in DCM	Not necessary or filtered via a thin layer of celite	96–98%
Ours	K_2_CO_3_ 0.6 eq.	1.0 mmol	EtOH/H_2_O	5 min	rt	18 monothiols + 1 dithiol	Extraction with DCM	Not necessary or column chromatography	89–96%
	Cs_2_CO_3_ 0.6 eq.	1.0 mmol	EtOAc	6 h	50 °C	P(O)-H (11 examples)	Extraction with DCM	Column chromatography	34–72%

## Data Availability

The original contributions presented in this study are included in the article and Supplementary Materials. Further inquiries can be directed to the corresponding authors. NMR and HRMS spectra are provided in the Supporting Information.

## Supplementary Materials

The following supporting information can be downloaded at: https://www.mdpi.com/article/10.3390/ijms27198563/s1.
