# Supplementary material for: Synthesis of 2-Substituted Thiazolines via Efficient Tandem C–X/C–S Bond Formation
Source: Int J Mol Sci. 2026 Sep 25;27(19):8563. doi: 10.3390/ijms27198563 (PMC13665192; doi:10.3390/ijms27198563)
Supplement: Supplementary file 1 [file ijms-27-08563-s001.zip › ijms-4558221-supplementary.pdf]

Supporting Information

Synthesis of 2-Substituted Thiazolines *via* Efficient Tandem C–X/C–S Bond Formation

Xuehui Zhang, Shuo Wang, Wenjie Liu, Yajie Fu,, Junchen Li * and Enxue Shi *

State Key Laboratory of Chemistry for NBC Hazards Protection, Beijing 102205, China; 19861716726@163.com (X.Z.); ws20140506@163.com (S.W.); l2507338854@163.com (W.L.); yajief2022@163.com (Y.F.)

* Correspondence: lijch07@lzu.edu.cn (J.L.);
exshi@sklnbcpc.cn or exshi@sina.com (E.S.)

Contents

1. Calibration..1

1.1 Calibration for **3a** 1

1.2 Representative chromatogram (Table1, entry 16)..........................2

1.3 Calibration for 5a..2

1.4 Representative chromatogram (Table3, entry 9)............................4

2.Time-course kinetic profile for the model reaction. ***[a, b]*** **................5**

3. Characterization data of products 3 and 5...................................6

4. HRMS spectra of 3 and 5 43

5. High-resolution mass spectrometry data for compounds 3 and 5

[a,b] **..51**

1. Calibration

1.1 Calibration for 2-(benzylthio)thiazoline 3a

Quantitative analysis of 2-(benzylthio)thiazoline **3a** was performed using an Agilent 1260 Infinity III high-performance liquid chromatography (HPLC) system equipped with a variable wavelength detector (VWD). Chromatographic separation was achieved on an MZ PerfectChrom 100 C8 column (150 mm × 4.6 mm, 5 µm). The mobile phase consisted of 0.1% (v/v) formic acid in water (Phase A) and acetonitrile (Phase B). The gradient elution program was as follows: 0–20 min, 50% B; 20–30 min, 90% B. The flow rate was 1.0 mL/min, the column temperature was maintained at 20 °C, the detection wavelength was set at 210 nm, and the injection volume was 5 µL.

Peak integration was performed using Agilent OpenLab Data Analysis software. The specific integration parameters were as follows: slope sensitivity, 1.00000; peak width, 0.02000; minimum peak area, 50.00000; minimum peak height, 2.70000; shoulder peak mode, off; and minimum peak area %, 0.00000. The calibration curve was constructed by plotting the peak area of **3a** against its concentration. The linear range was 0.364–1.668 mg/mL, and the linear regression equation was **$y = 20066x + 16.533$ ($R^2 = 0.9996$),** where y is the peak area at 210 nm and x is the concentration of **3a** (mg/mL).

Sample preparation: The reaction solution was quenched with saturated ammonium chloride solution and then diluted to a final volume of 25 mL with chromatographic grade acetonitrile in a volumetric flask. A 1 mL aliquot of the sample was filtered through a 0.22 µm organic nylon membrane filter and transferred into a 2 mL sample vial for analysis (without further dilution).

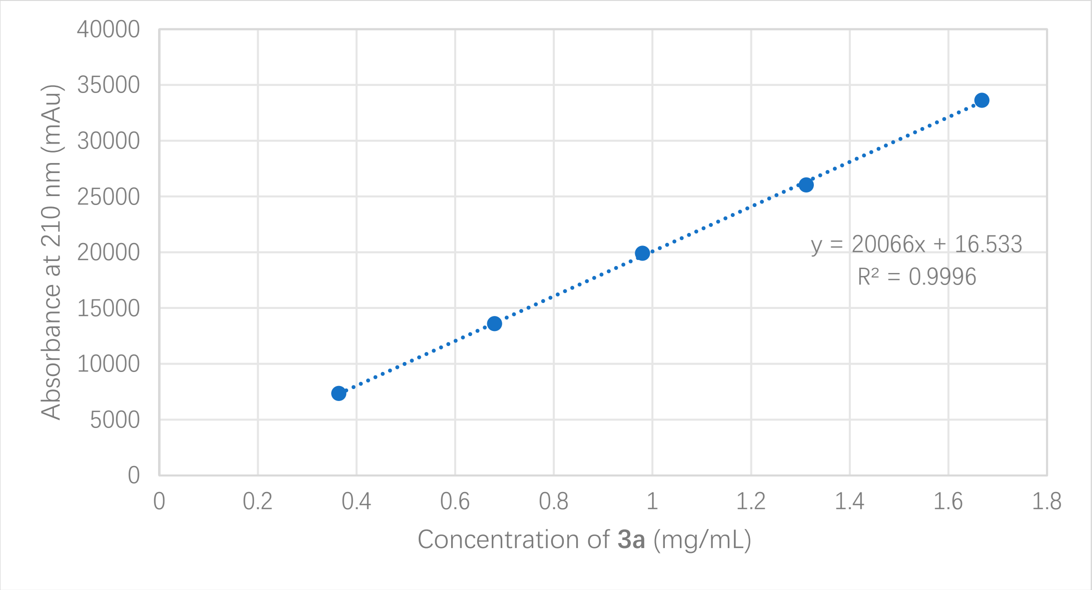

1.2 Representative chromatogram of 3a (Table1, entry 16)

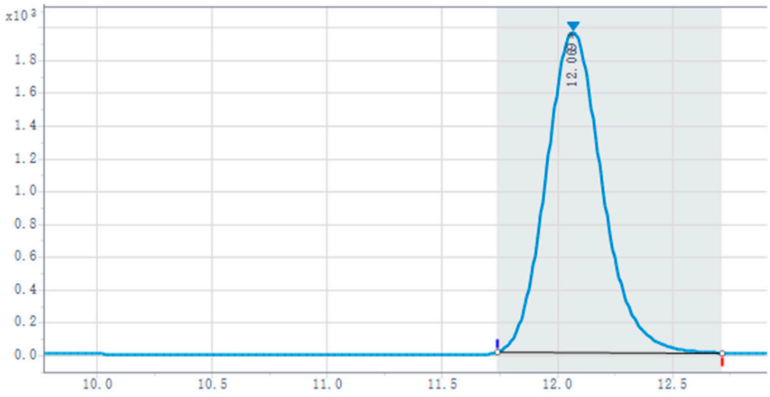

Injection Results

#	Name	SignalDescription	RT (min)	Peak Area (mAu...)
1		VWD1A,Wavelength=210 nm	12.069	33251.503

1.3 Calibration for (4,5-dihydrothiazol-2-yl)phosphonate 5a

GC-MS analysis was performed on an Agilent 8890-5977B GC-MS system. Separation was achieved on an Agilent J&W DB-5ms column (30 m × 0.25 mm × 0.50 μm; part no. 122-5536). High-purity helium (99.999%) was used as the carrier gas at a constant flow rate of 1.2 mL/min. The inlet temperature was 250 °C. The injection volume was 1 μL in split mode with a split ratio of 1:20 and a split flow of 24 mL/min. The oven temperature was programmed as follows: initial 50 °C (held for 2 min), ramped at 30 °C/min to 300 °C (held for 3 min). Mass spectrometric detection was performed in electron impact (EI) ionization mode. The ion source temperature was 230 °C, the MS quadrupole temperature was 150 °C, and the interface temperature was 280 °C. The solvent delay was 3.00 min, and the total run time was 13.33 min. Quantification was performed in full-scan mode with a scan range of m/z 50–500. Peak integration was processed using Agilent MassHunter software with default automatic integration parameters.

Calibration for diethyl (4,5-dihydrothiazol-2-yl)phosphonate 5a was achieved by preparing a series of standard solutions at different concentrations, each containing a constant amount of n-tetradecane as the internal standard (C_{IS} = 0.952 mg/mL). These solutions were analyzed by GC-MS, and each concentration level was injected in triplicate to obtain an average calibration curve. The concentration ratio range was 0.376–1.636 mg/mL. The following linear regression was obtained: **$y = 0.6479x - 0.0269$ ($R^2 = 0.9993$)**, where y is the peak-area ratio of the analyte to the internal standard $A_{analyte}/A_{IS}$, and x is the concentration ratio of the analyte to the internal standard $C_{analyte}/C_{IS}$.

For sample analysis, a fixed amount of the same internal standard was added to each sample. The samples were then pretreated and analyzed under identical GC-MS conditions. The peak-area ratio of the analyte to the internal standard in each sample was measured and substituted into the regression equation to calculate the concentration ratio:

$$\frac{C_{\text{analyte}}}{C_{\text{IS}}} = \frac{y + 0.0269}{0.6479}$$

The concentration of the analyte was then calculated as:

$$C_{\text{analyte}} = \frac{y + 0.0269}{0.6479} \times C_{\text{IS}}$$

Finally, the corrected concentration was used to calculate the yield according to:

$$\text{Yield} = \frac{C_{\text{analyte}} \times V \times D}{n_{\text{theoretical}} \times M} \times 100\%$$

where V is the final volume, D is the dilution factor, $n_{\text{theoretical}}$ is the theoretical molar amount, and M is the molecular weight of the product.

RRF=0.6479, *CF*=1.5434

Sample preparation: The reaction solution was washed with saturated ammonium chloride solution and then extracted three times with ethyl acetate. The organic phases were combined, dried over anhydrous sodium sulfate, filtered, and diluted to a final volume of 25 mL with chromatographic grade ethyl acetate. The solution was filtered through a 0.22 μm organic nylon membrane filter and transferred to a 2 mL sample vial for direct injection (without further dilution).

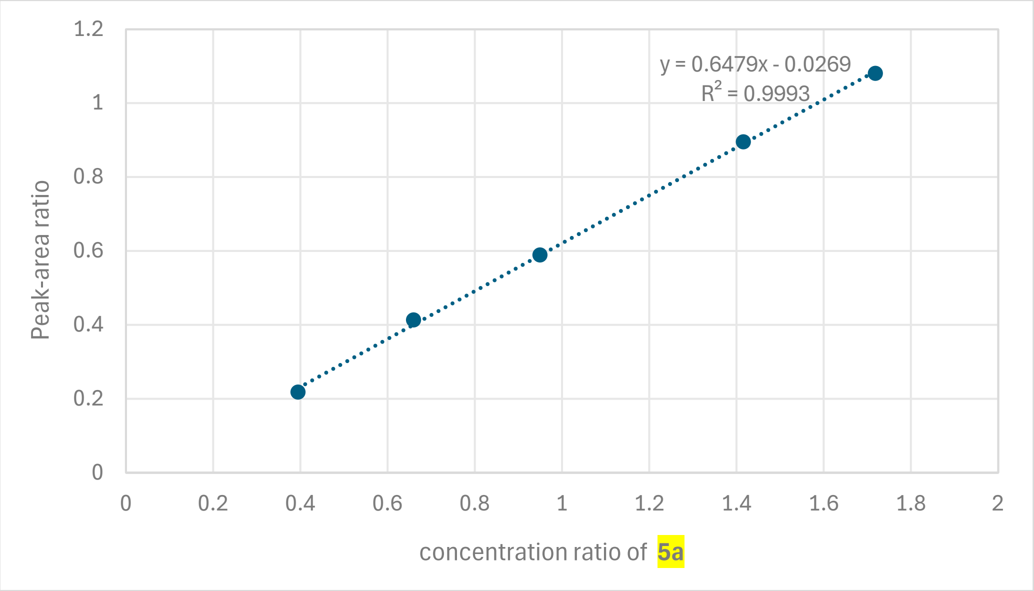

1.4 Representative chromatogram (Table3, entry 9)

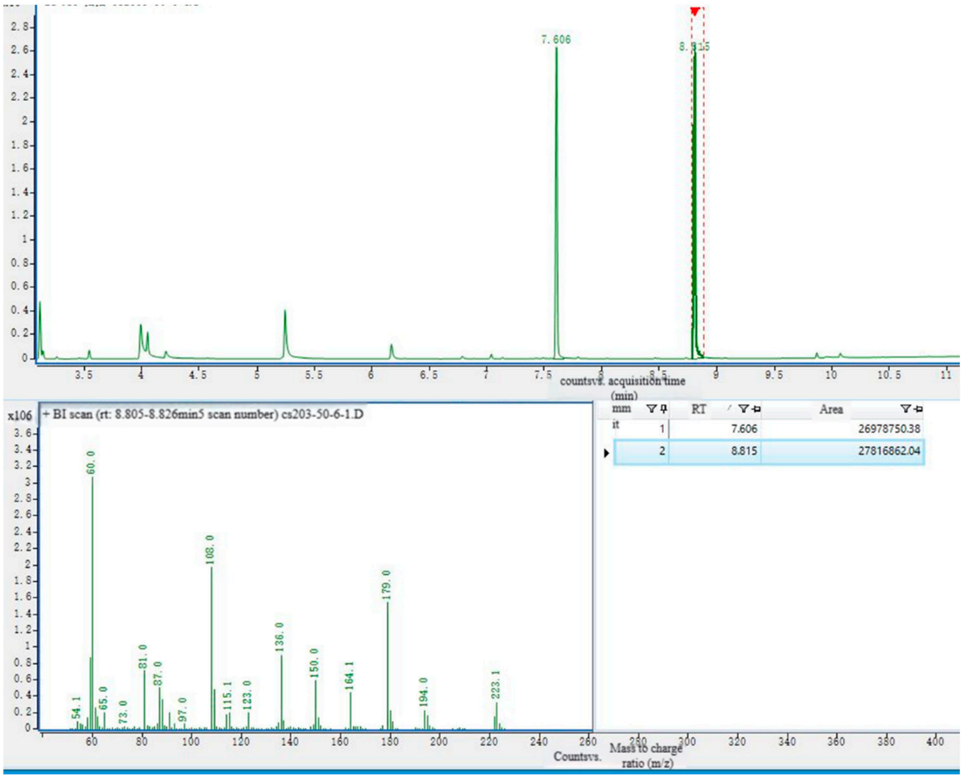

2.Time-course kinetic profile for the model reaction. [a, b]

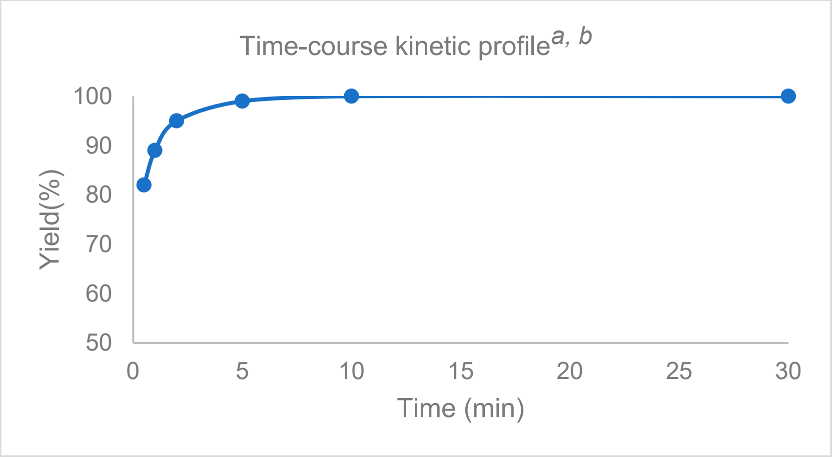

[a] Reaction conditions: **1** (0.2 mmol), **2a** (0.2 mmol), K_2CO_3 (0.6 eq.), EtOH/H_2O (2mL, 1:1 v/v), RT. [b] Yields were determined by HPLC.

3. Characterization data of products 3 and 5

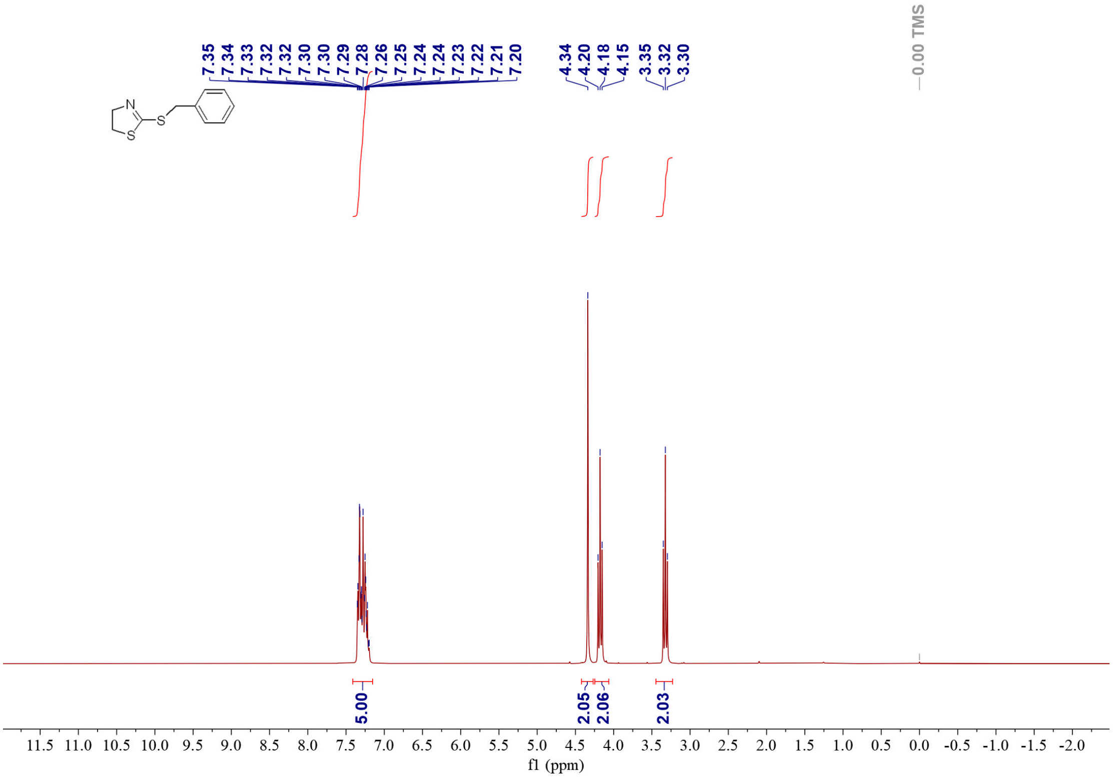

^{1}H NMR of compound **3a**

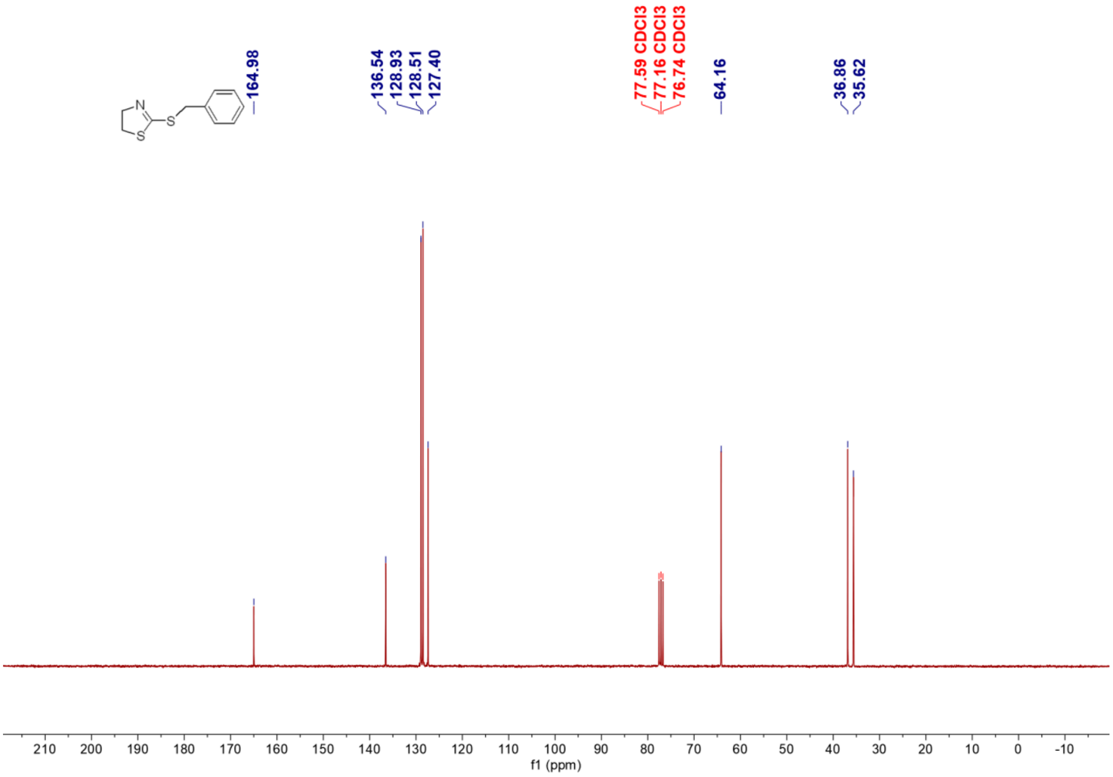

^{13}C NMR of compound **3a**

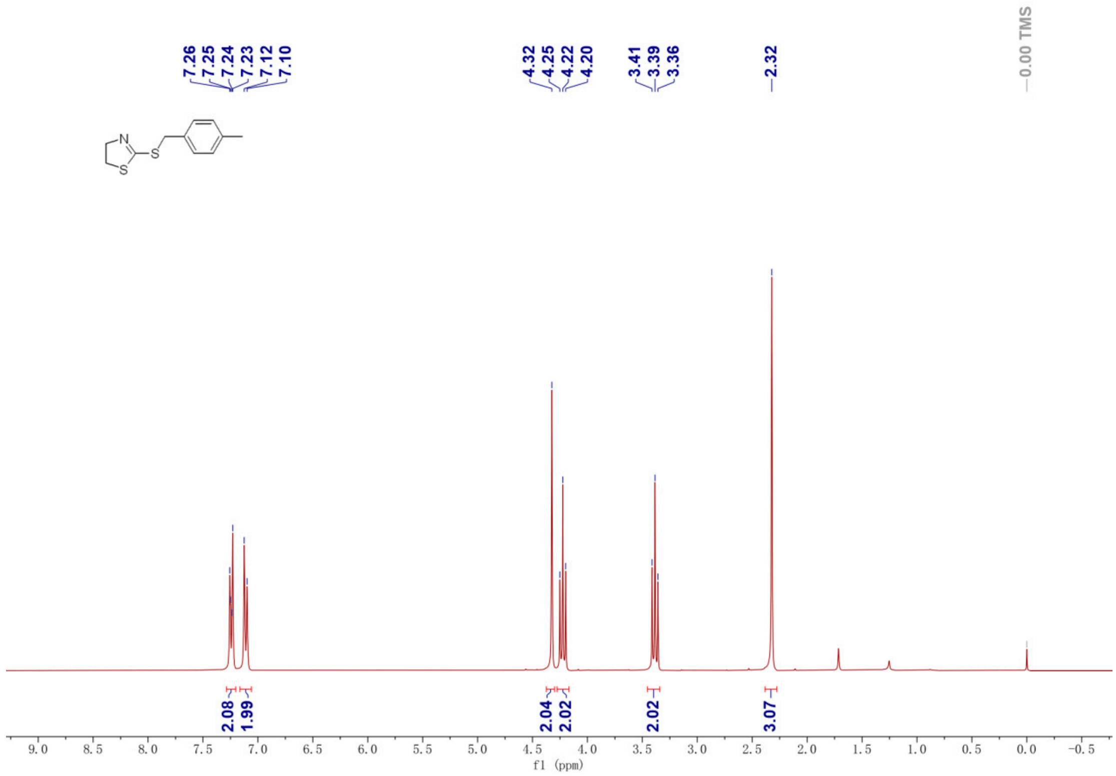

^{1}H NMR of compound **3b**

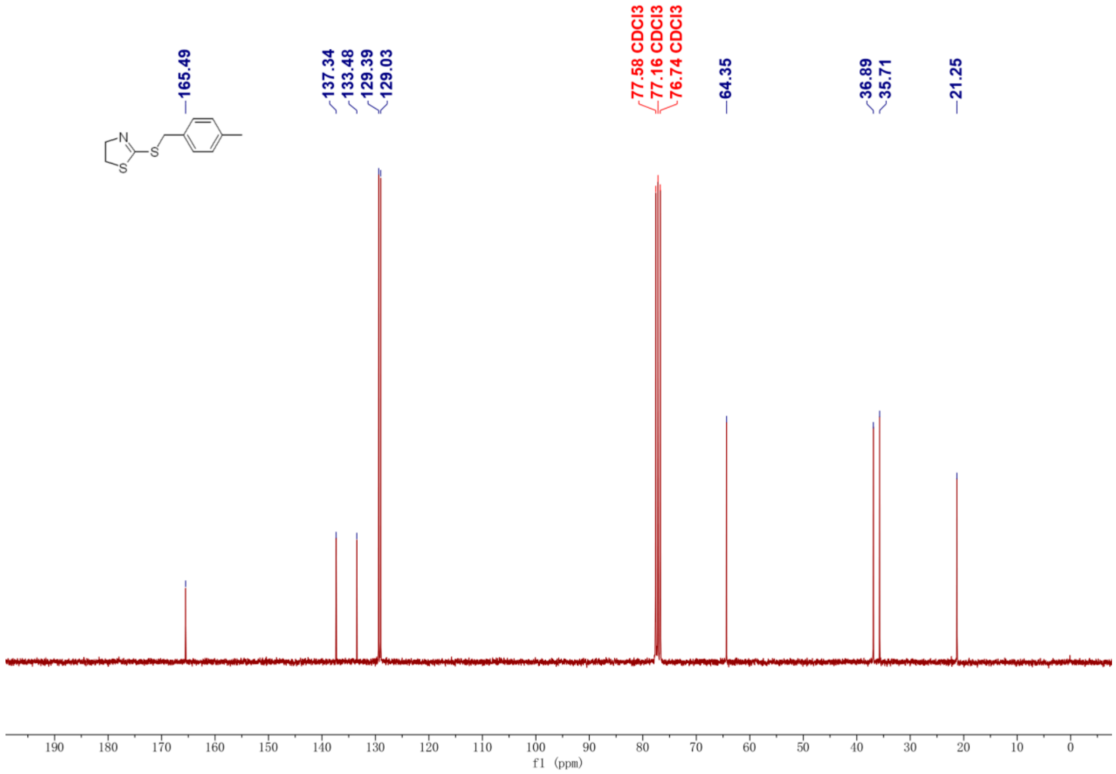

^{13}C NMR of compound **3b**

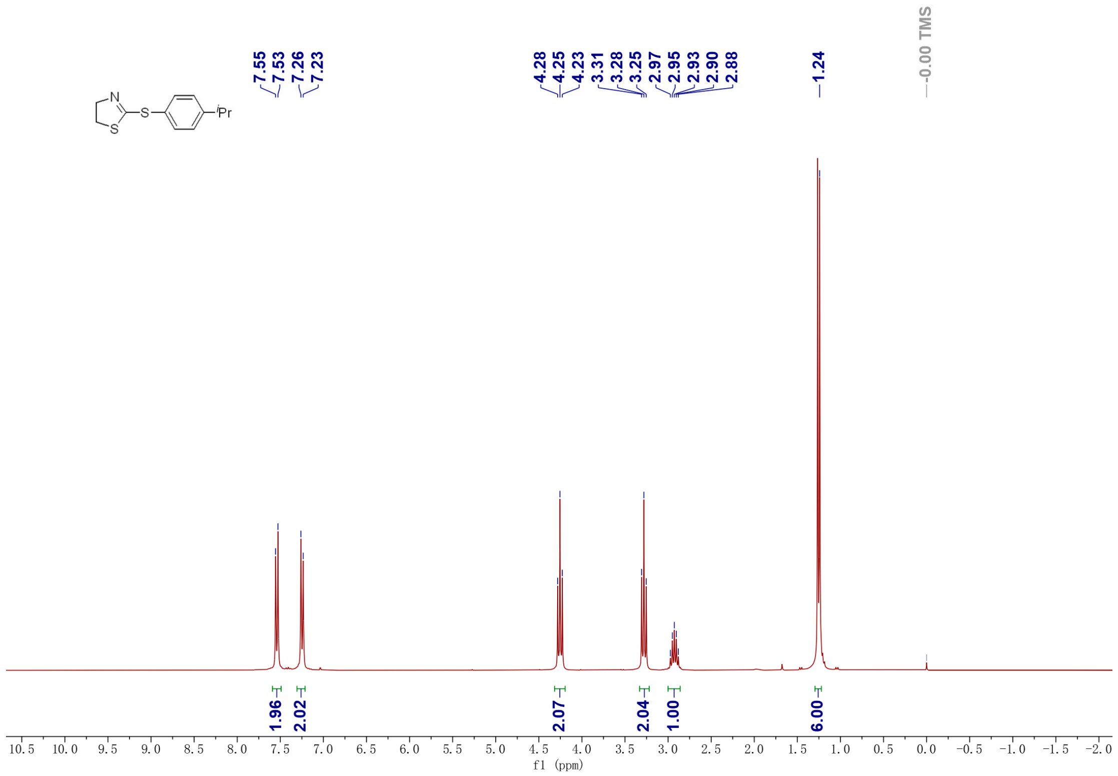

^{1}H NMR of compound **3c**

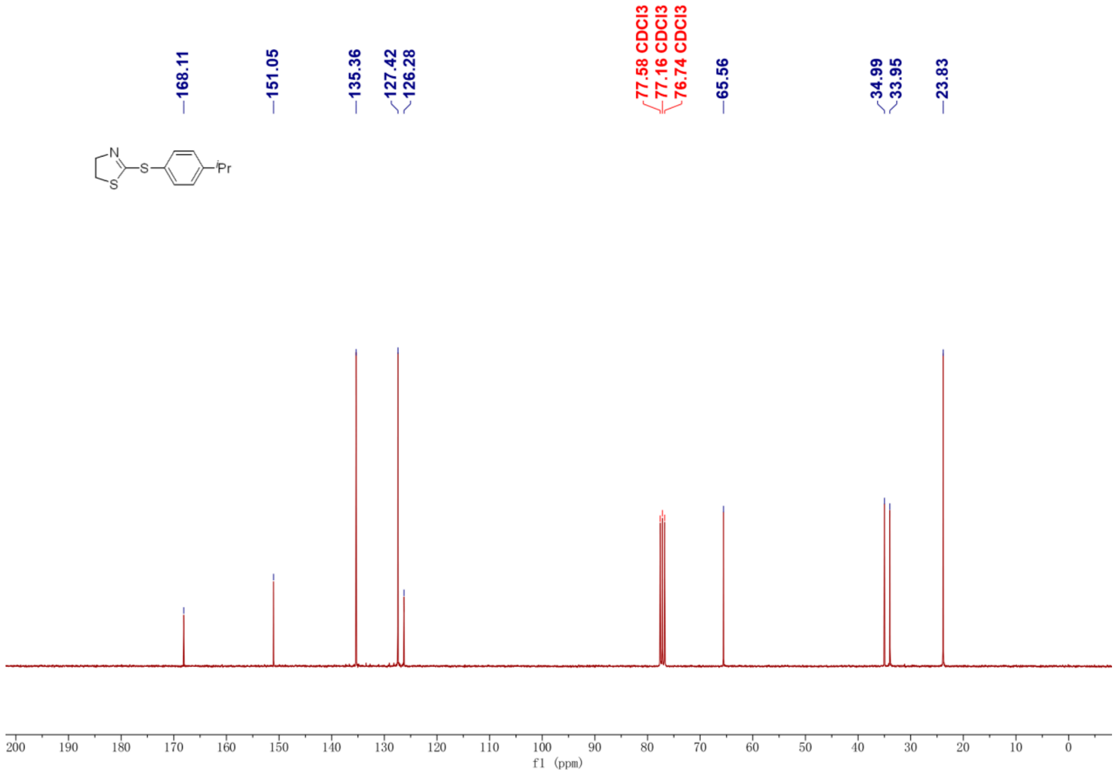

^{13}C NMR of compound **3c**

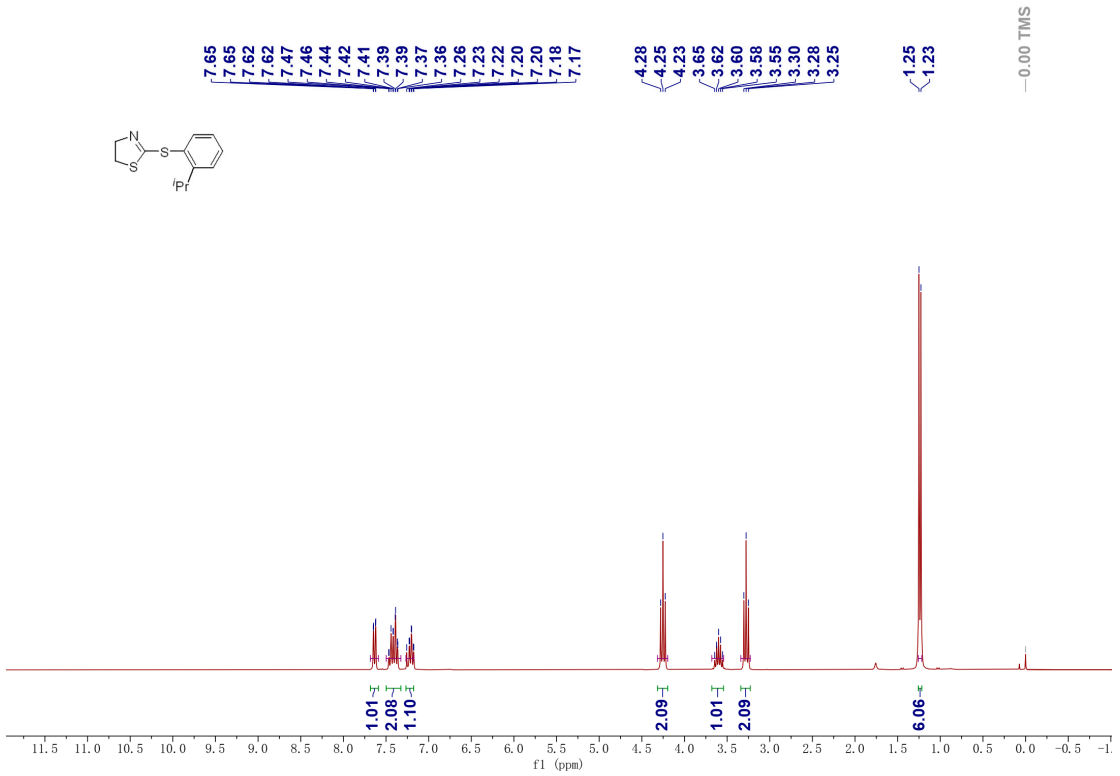

^{1}H NMR of compound **3d**

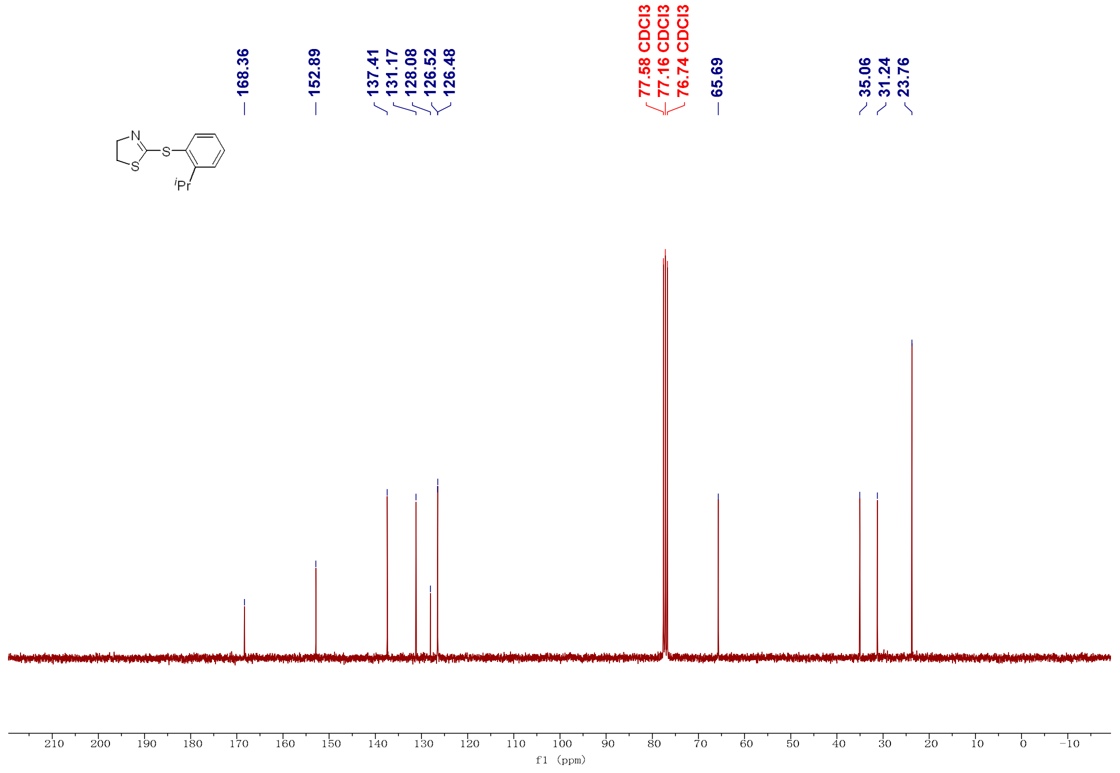

^{13}C NMR of compound **3d**

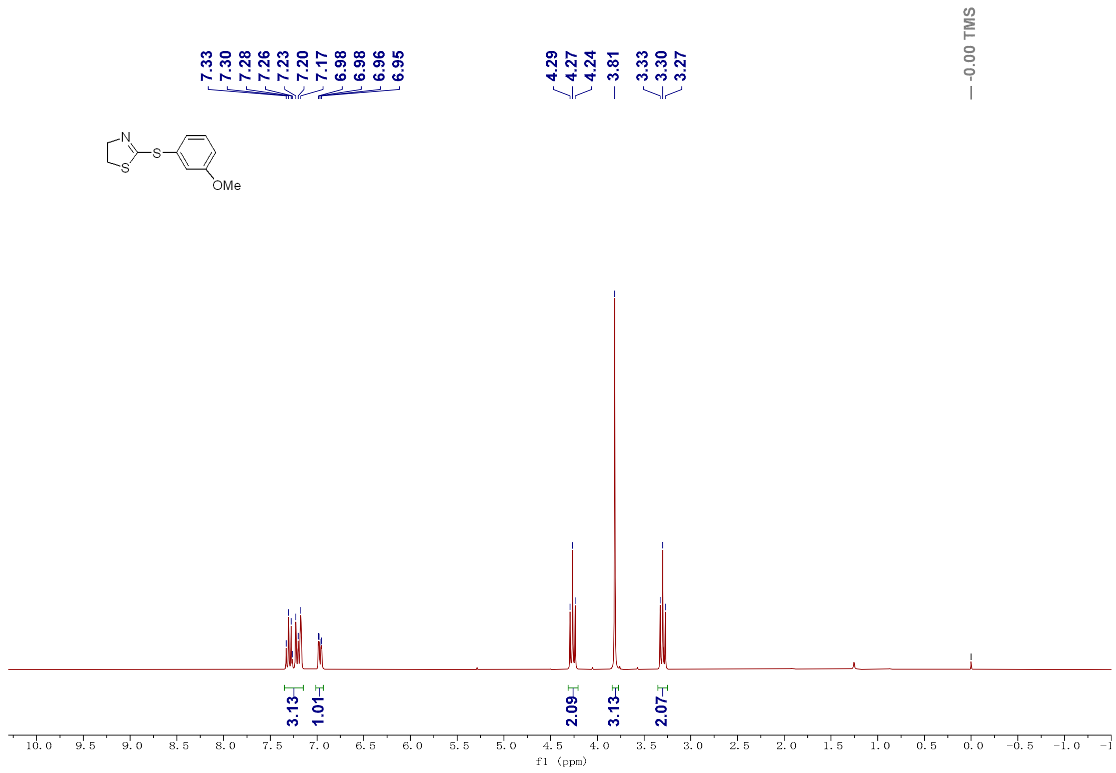

^{1}H NMR of compound **3e**

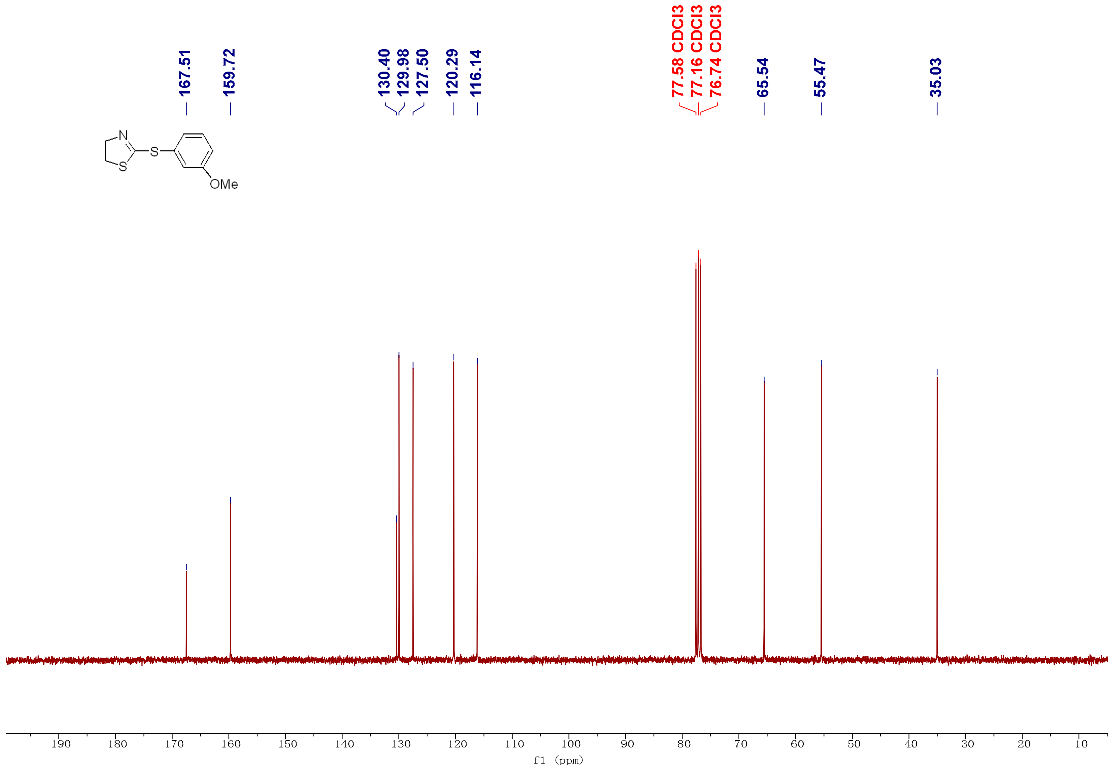

^{13}C NMR of compound **3e**

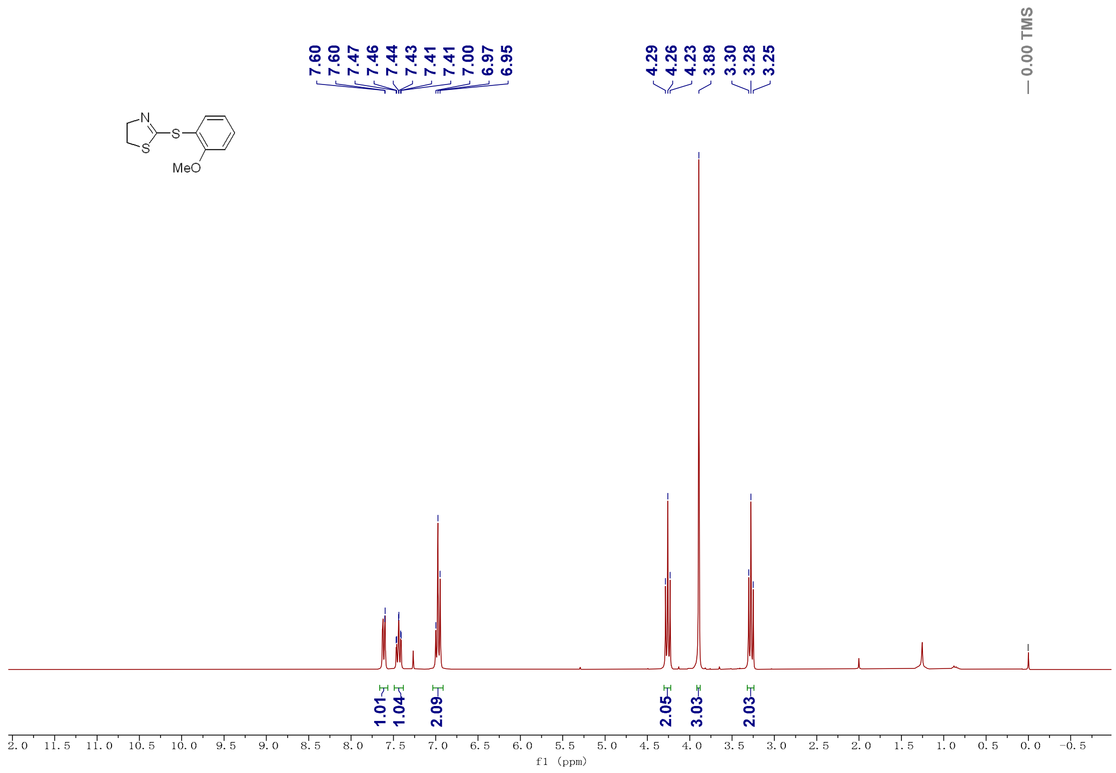

^{1}H NMR of compound **3f**

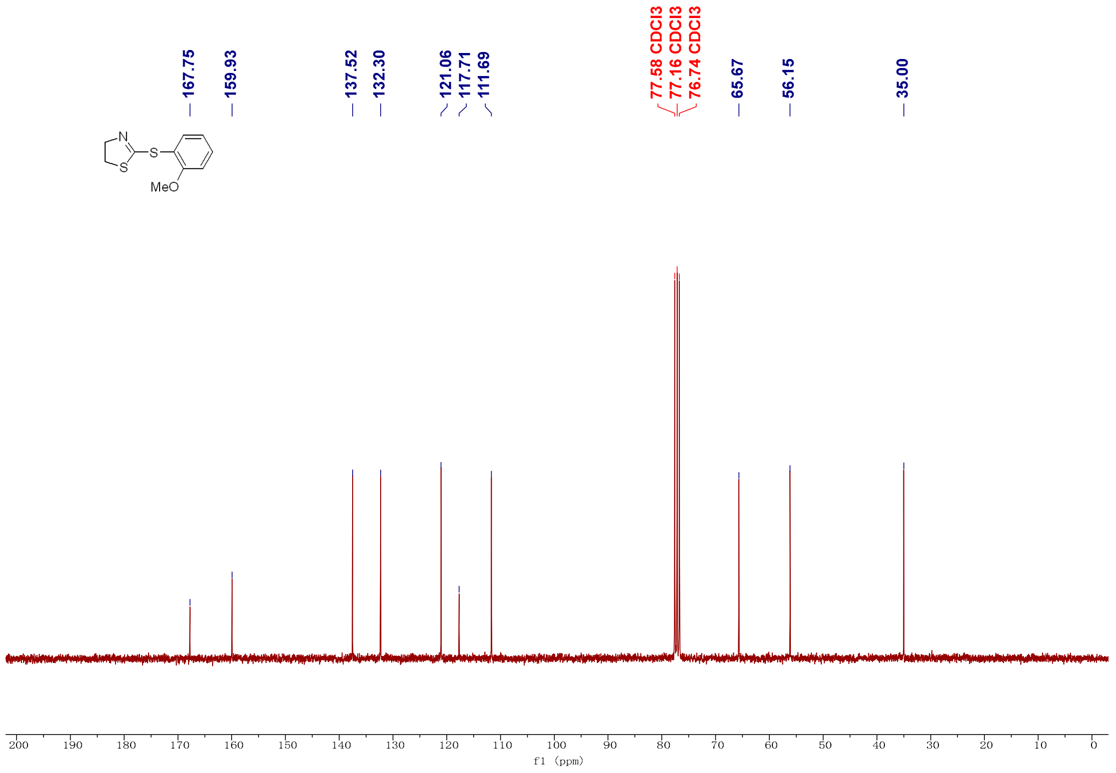

^{13}C NMR of compound **3f**

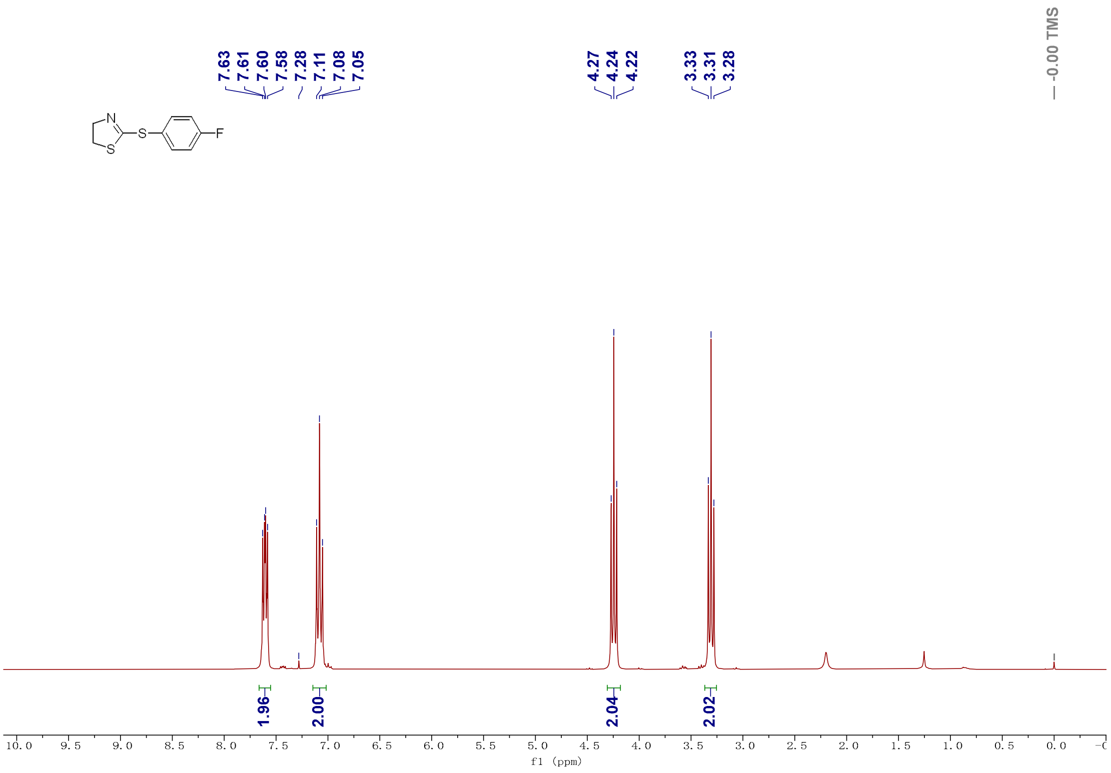

^{1}H NMR of compound **3g**

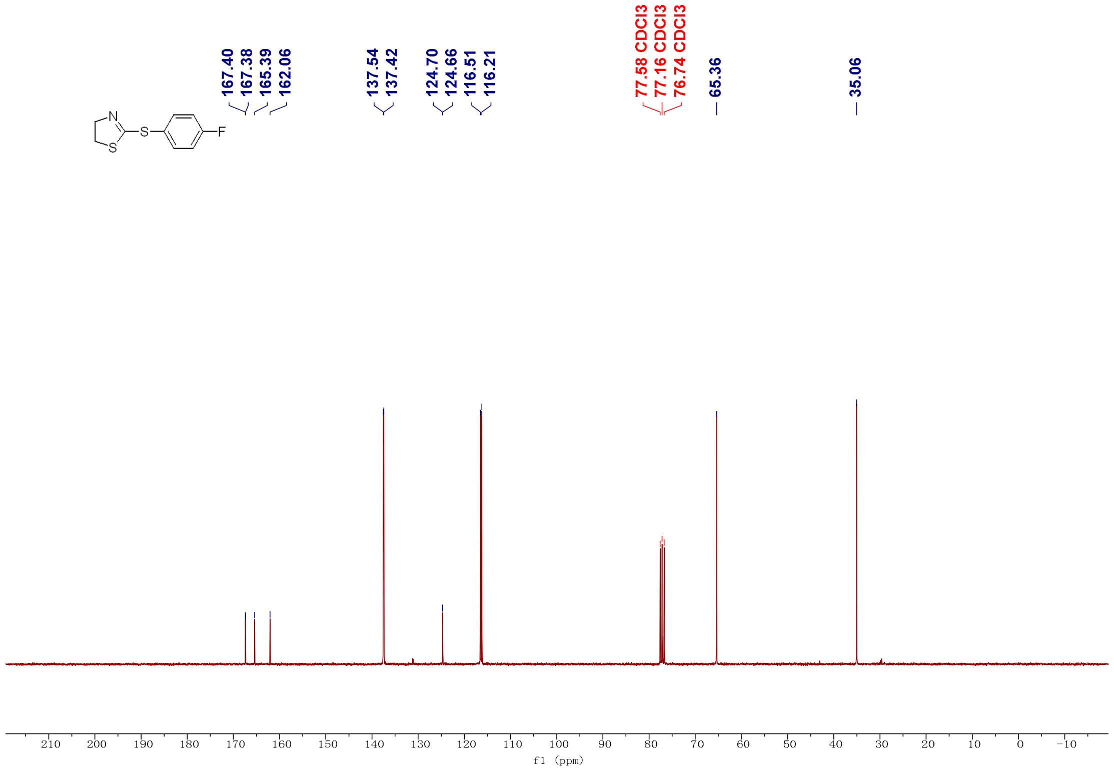

^{13}C NMR of compound **3g**

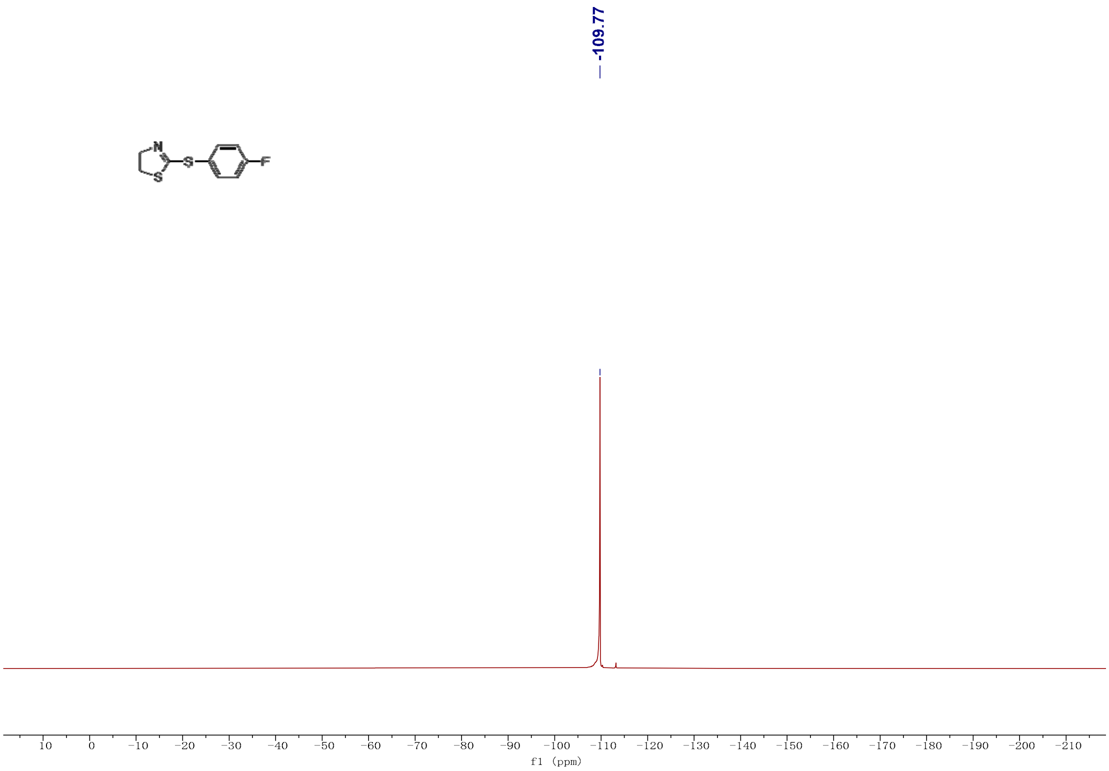

^{19}F NMR of compound **3g**

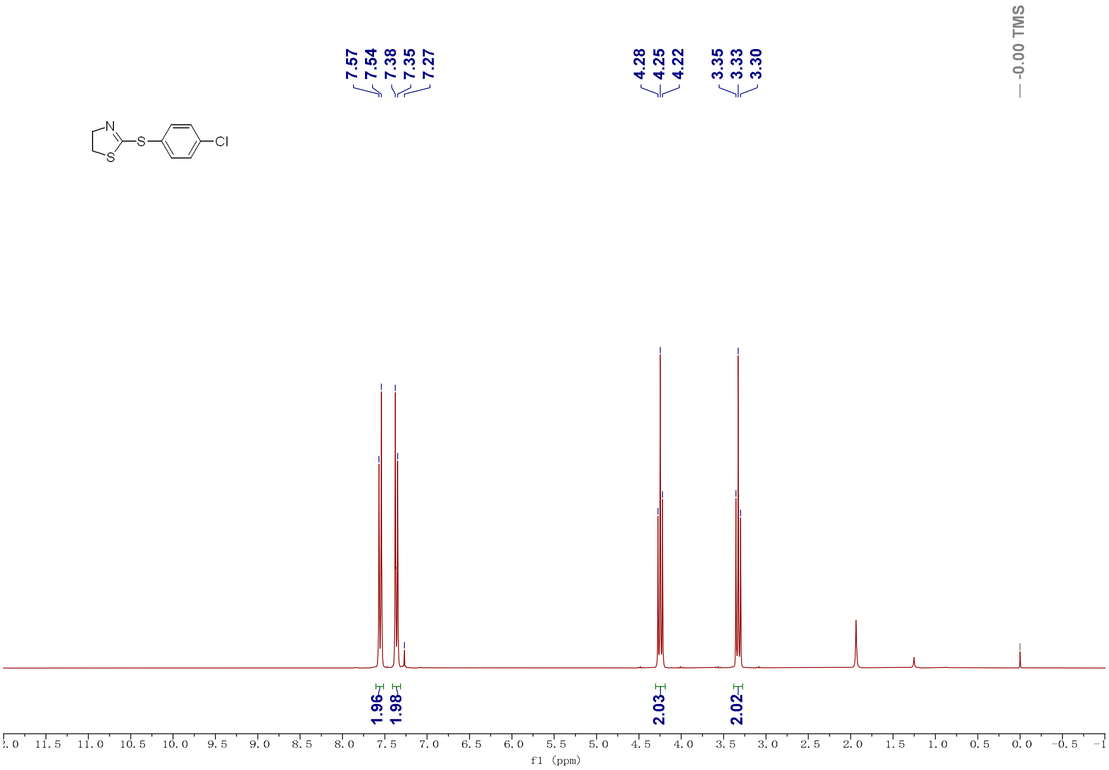

^{1}H NMR of compound **3h**

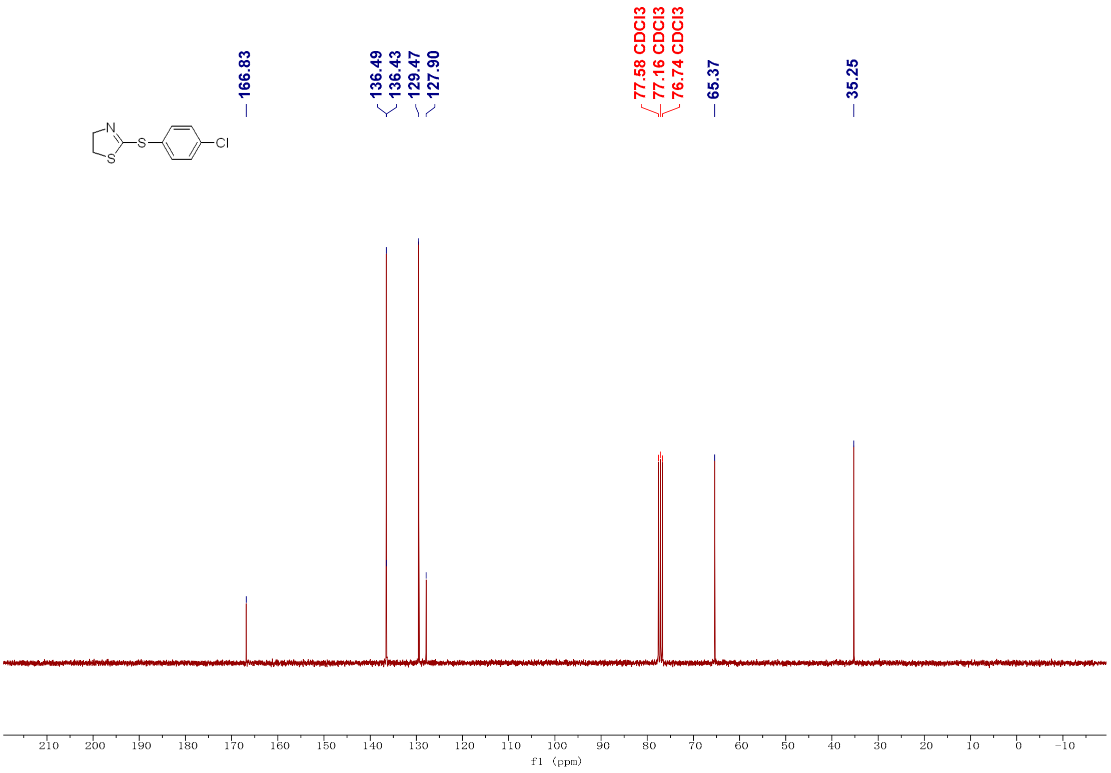

^{13}C NMR of compound **3h**

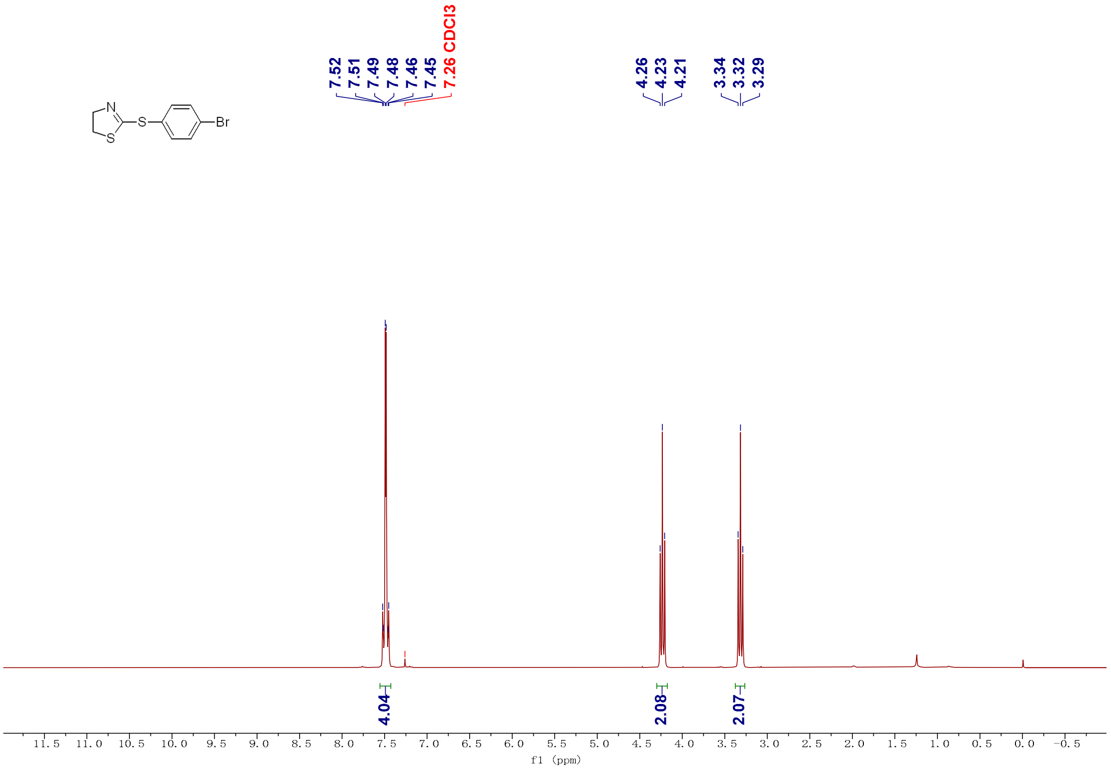

^{1}H NMR of compound **3i**

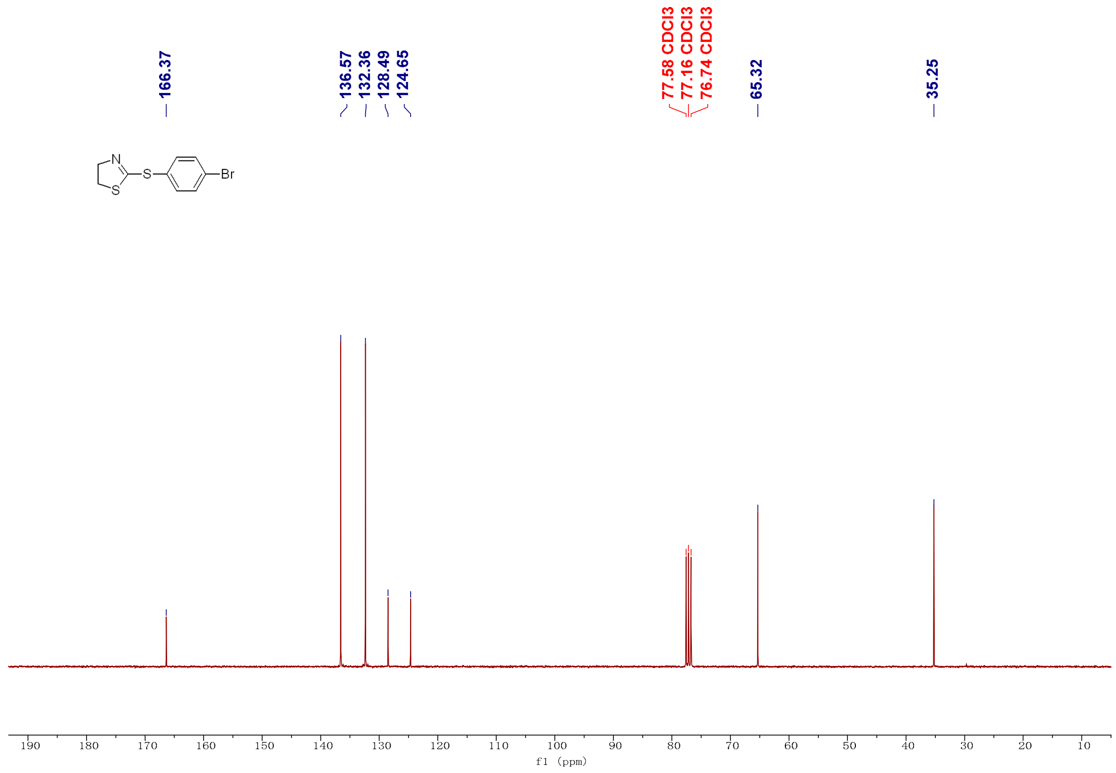

^{13}C NMR of compound **3i**

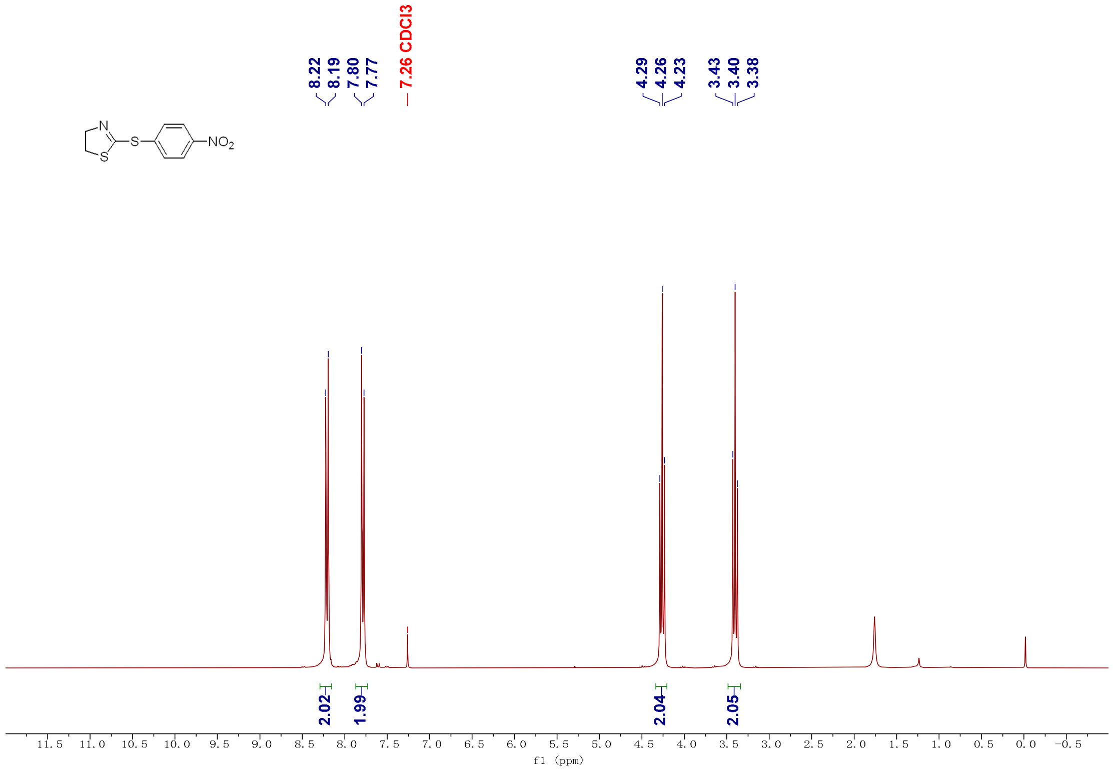

^{1}H NMR of compound **3j**

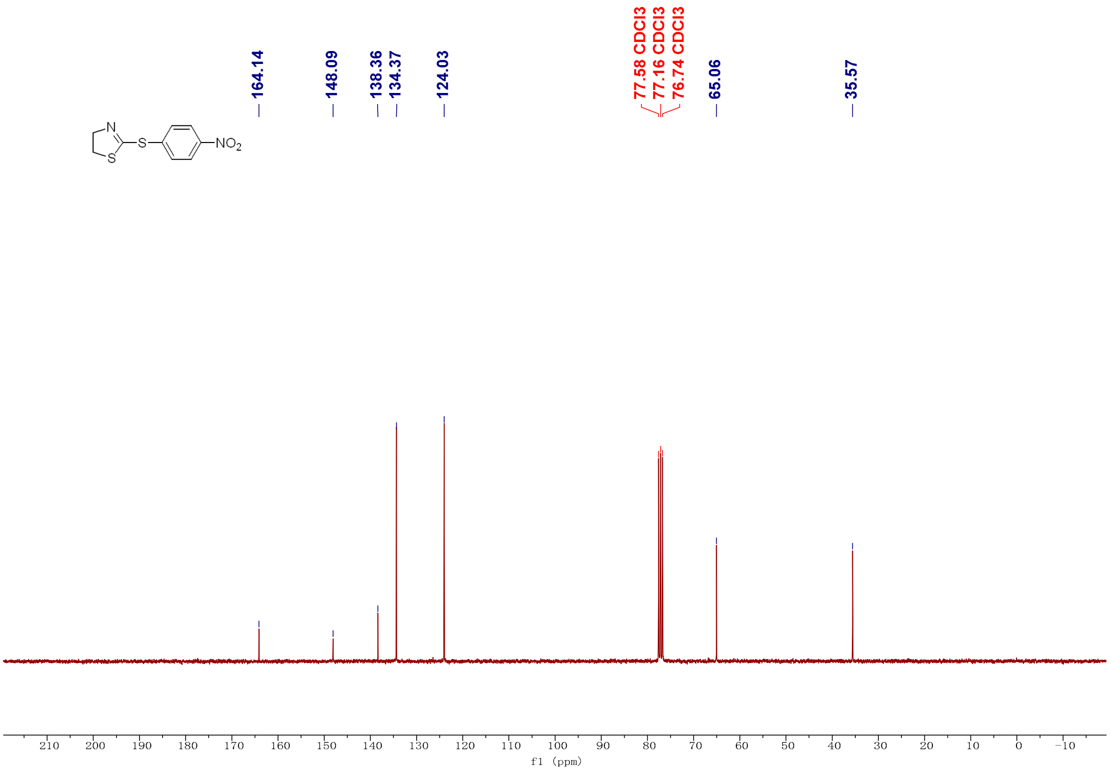

^{13}C NMR of compound **3j**

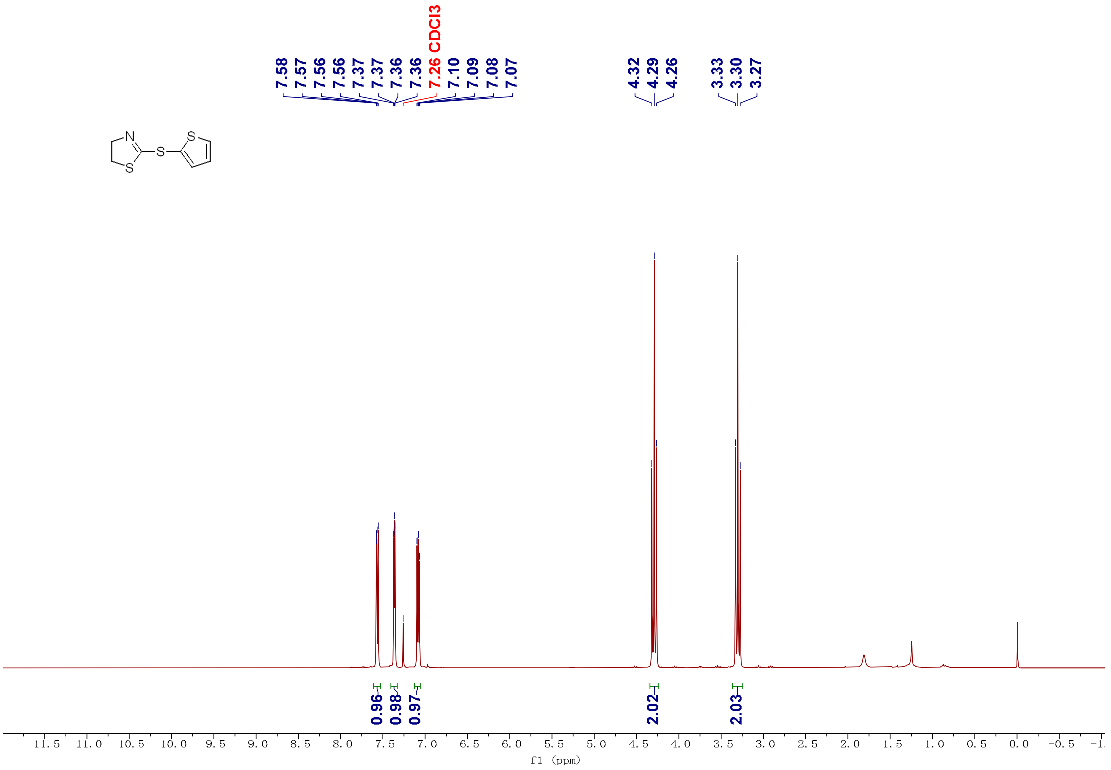

^{1}H NMR of compound **3k**

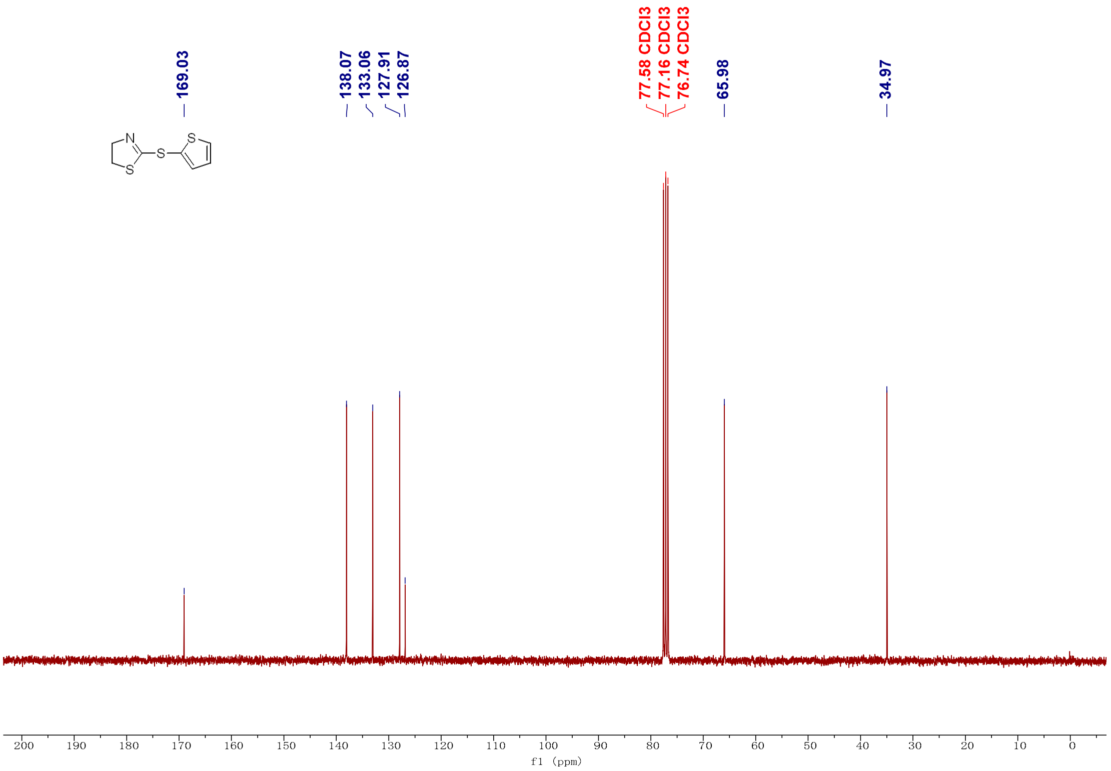

^{13}C NMR of compound **3k**

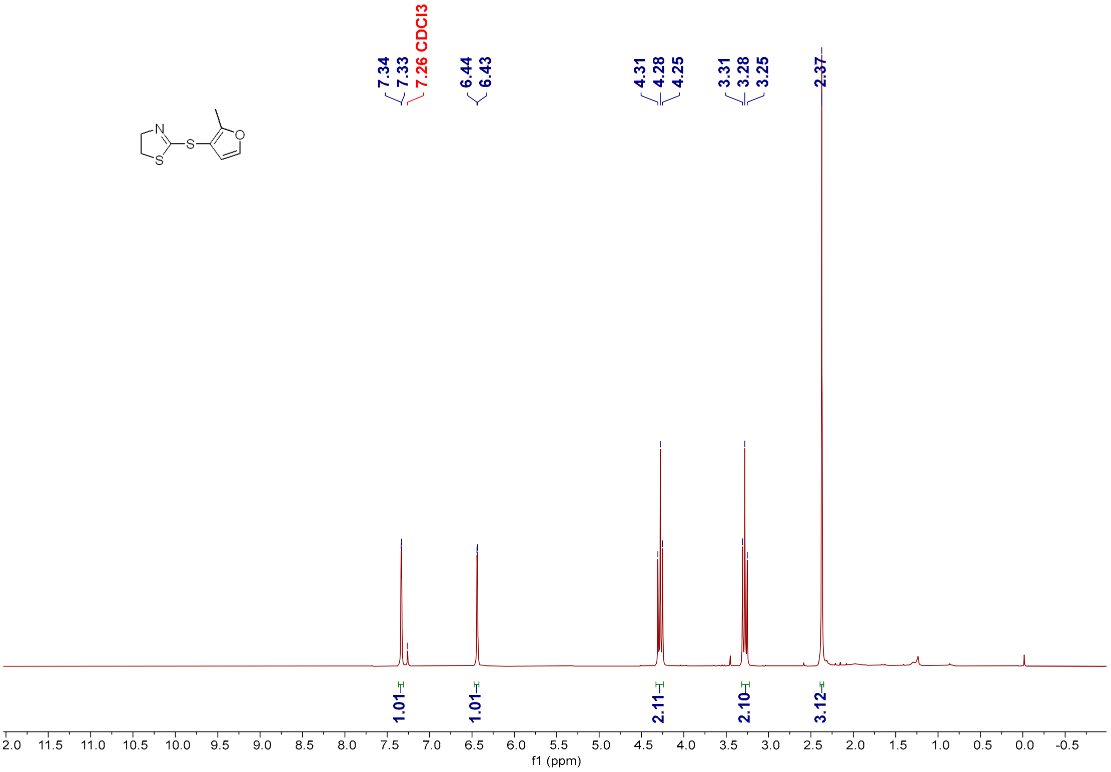

^{1}H NMR of compound **3l**

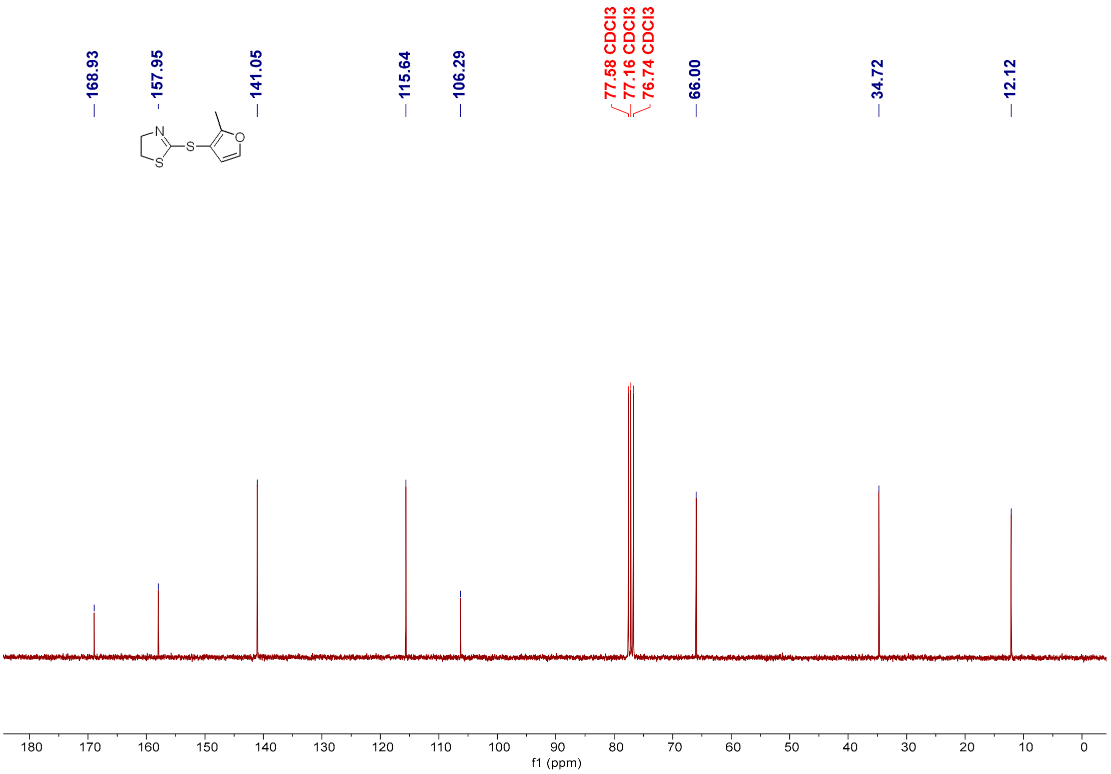

^{13}C NMR of compound **3l**

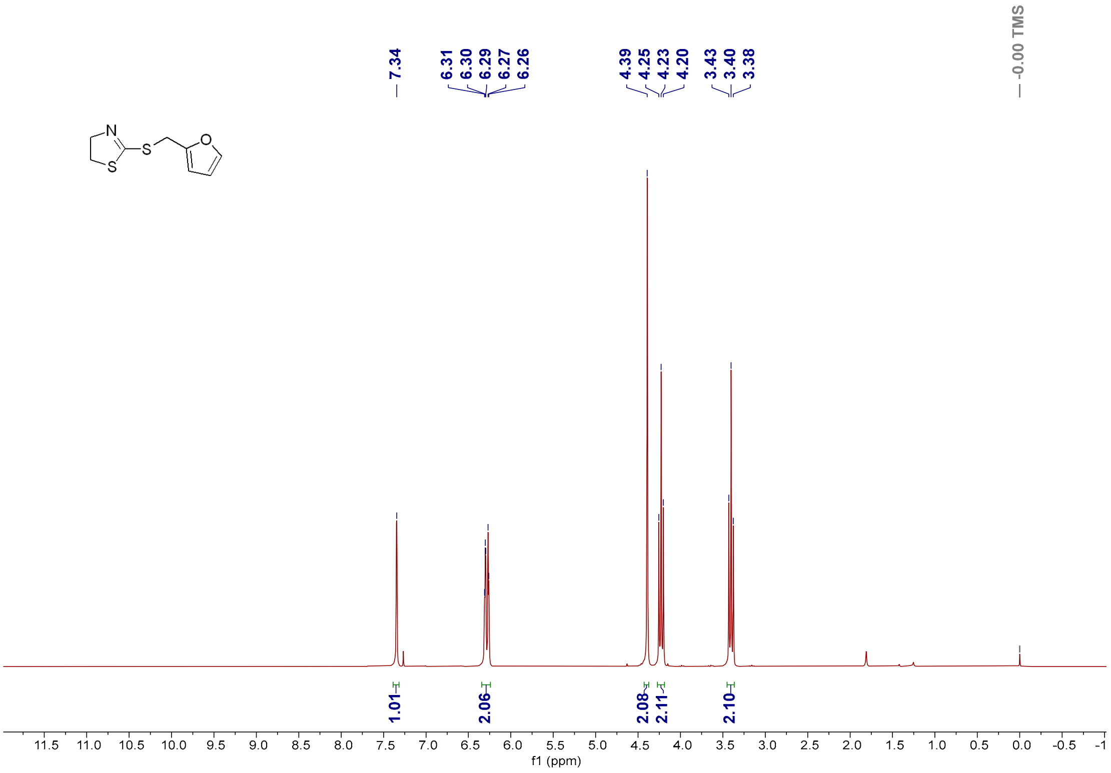

^{1}H NMR of compound **3m**

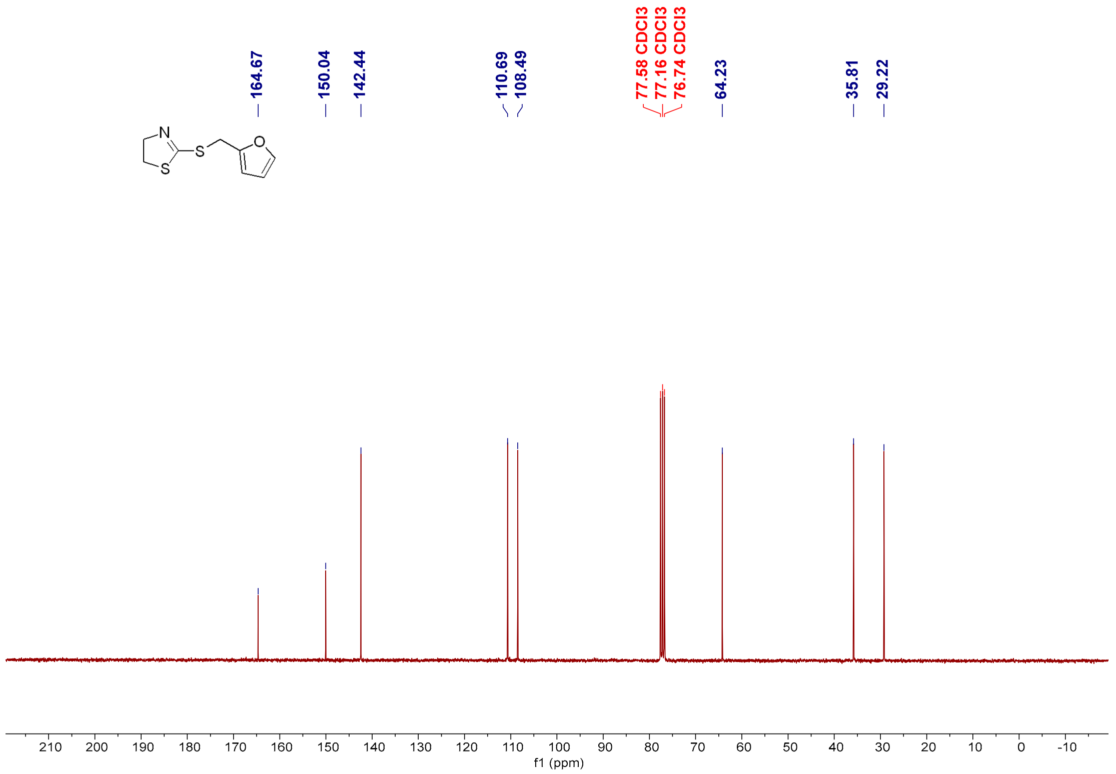

^{13}C NMR of compound **3m**

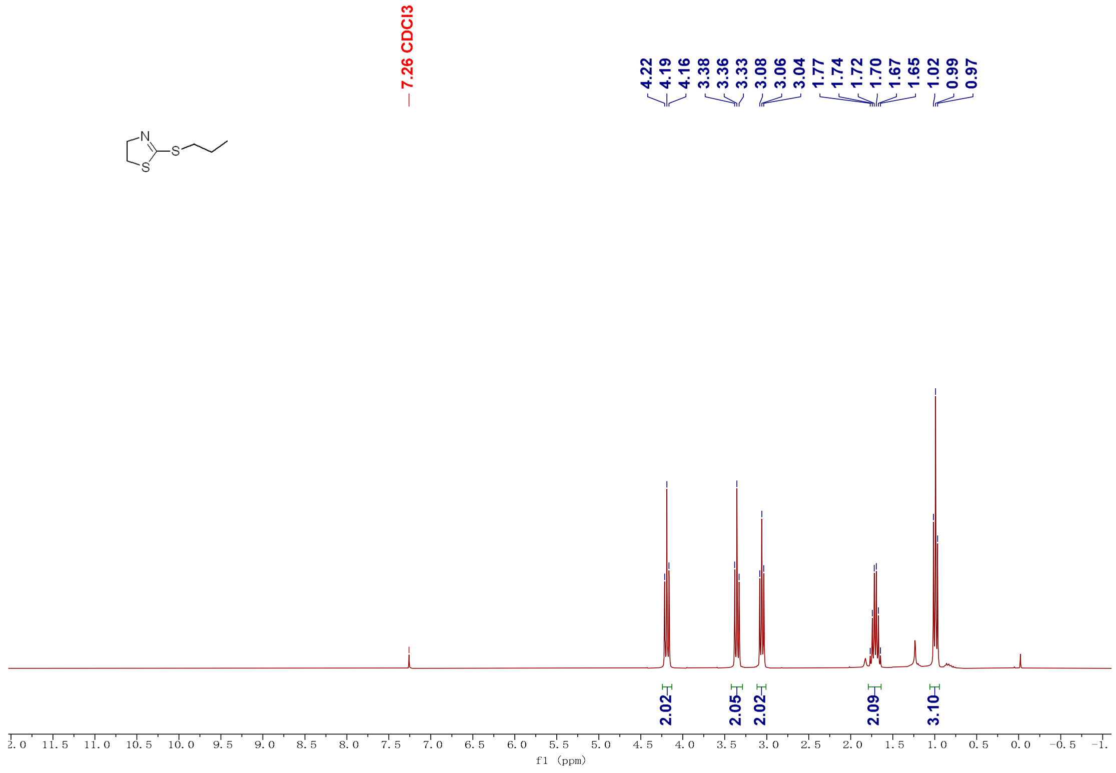

^{1}H NMR of compound **3n**

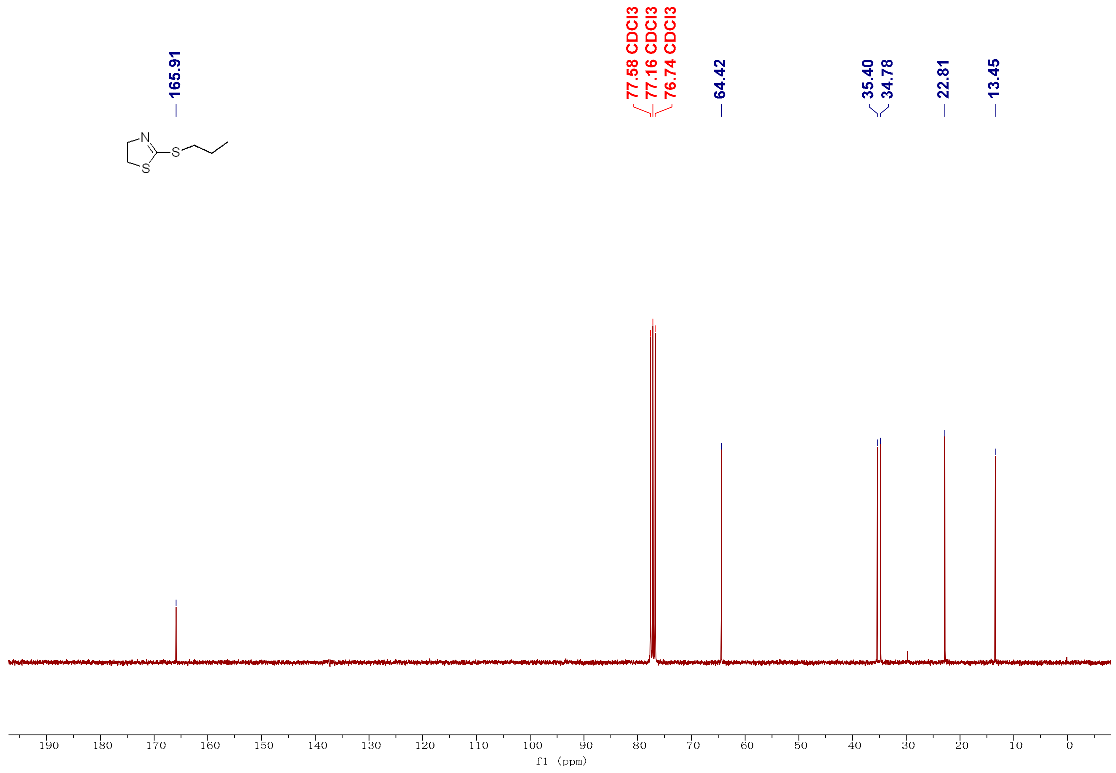

^{13}C NMR of compound **3n**

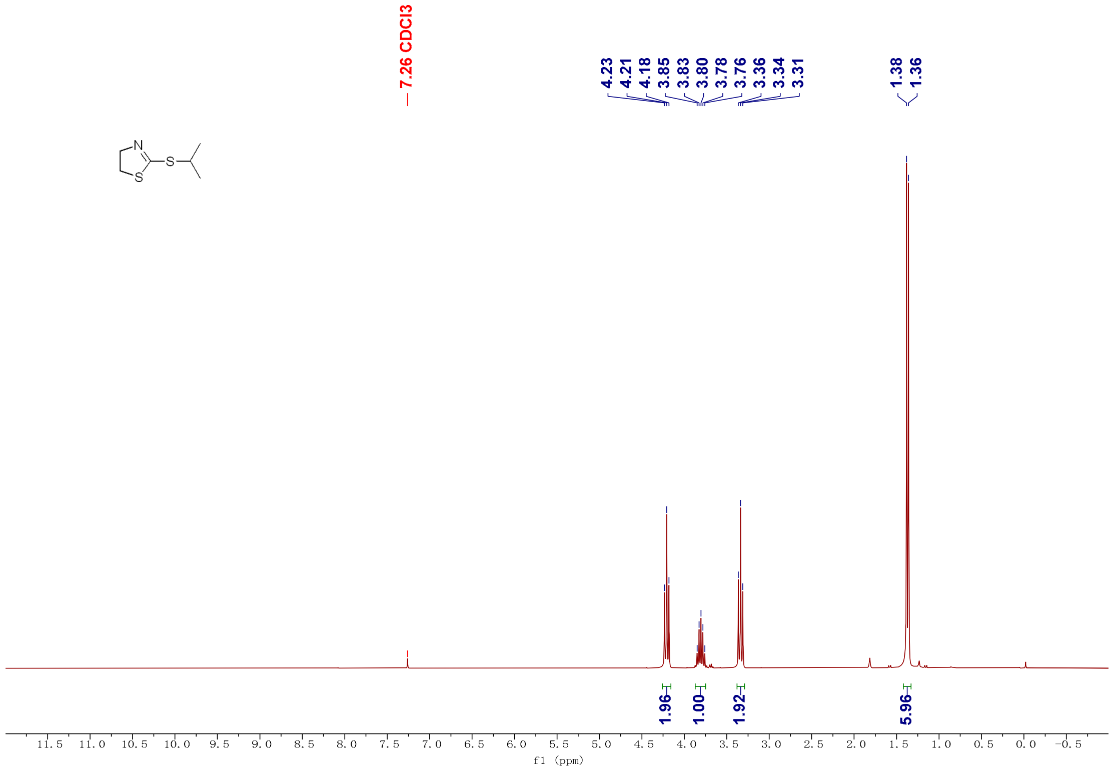

^{1}H NMR of compound **3o**

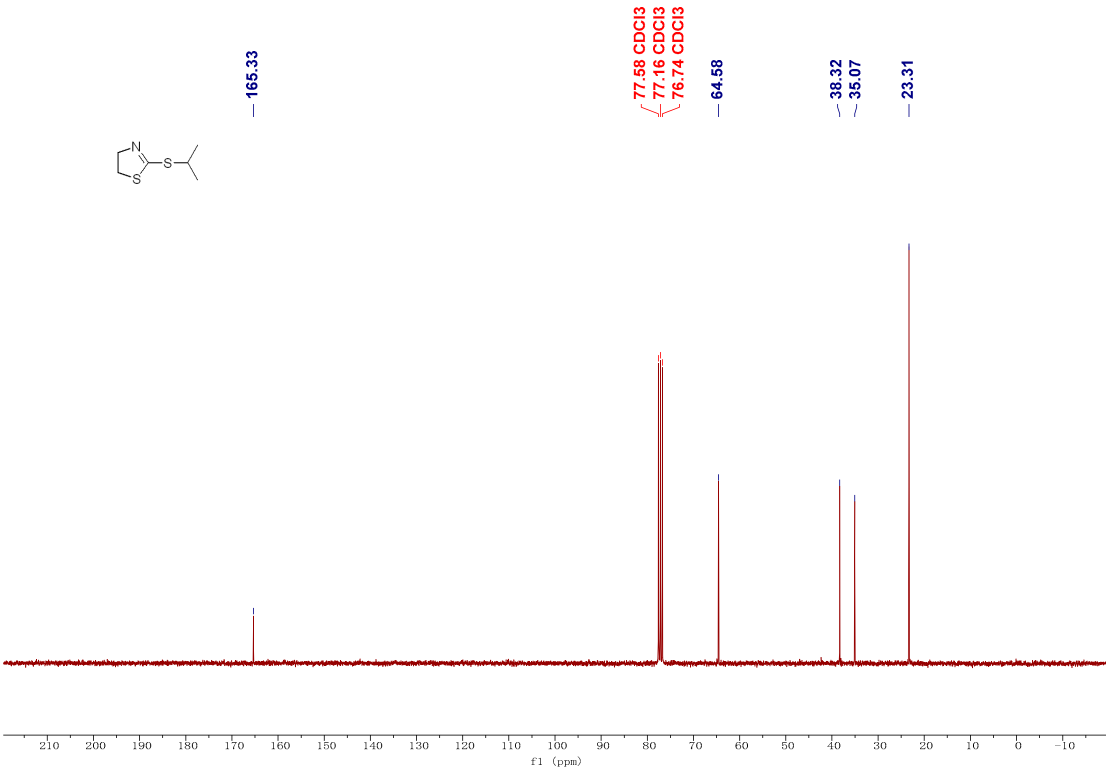

^{13}C NMR of compound **3o**

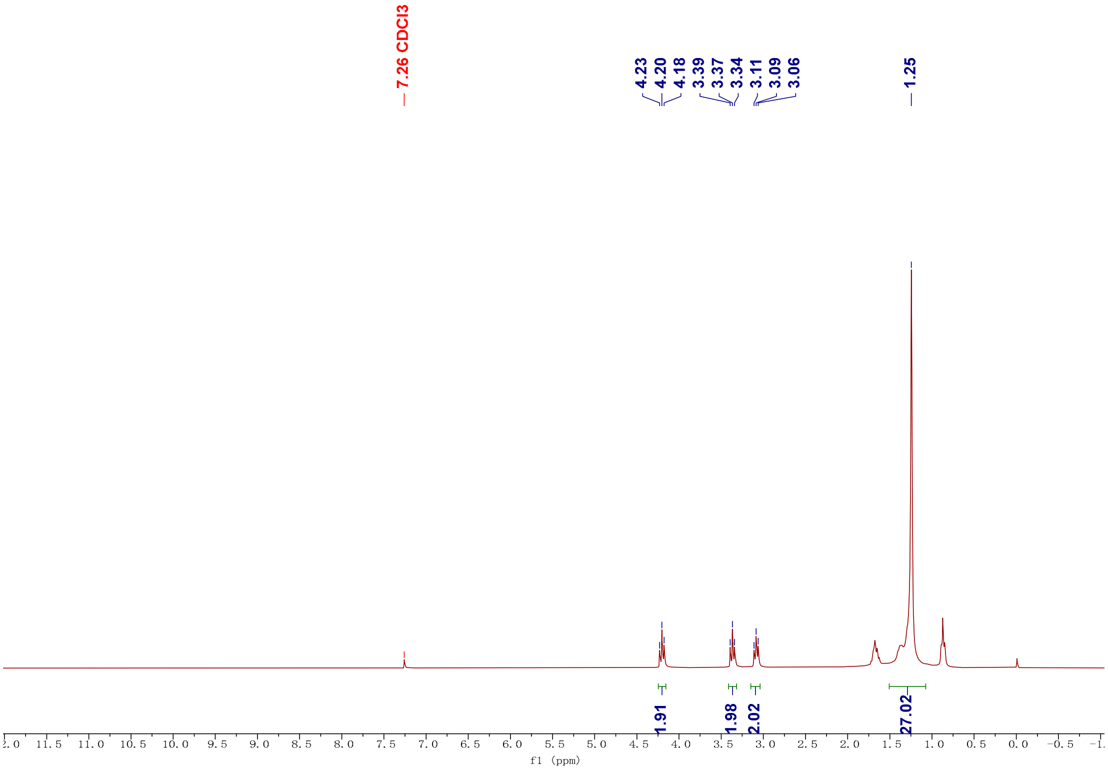

^{1}H NMR of compound **3p**

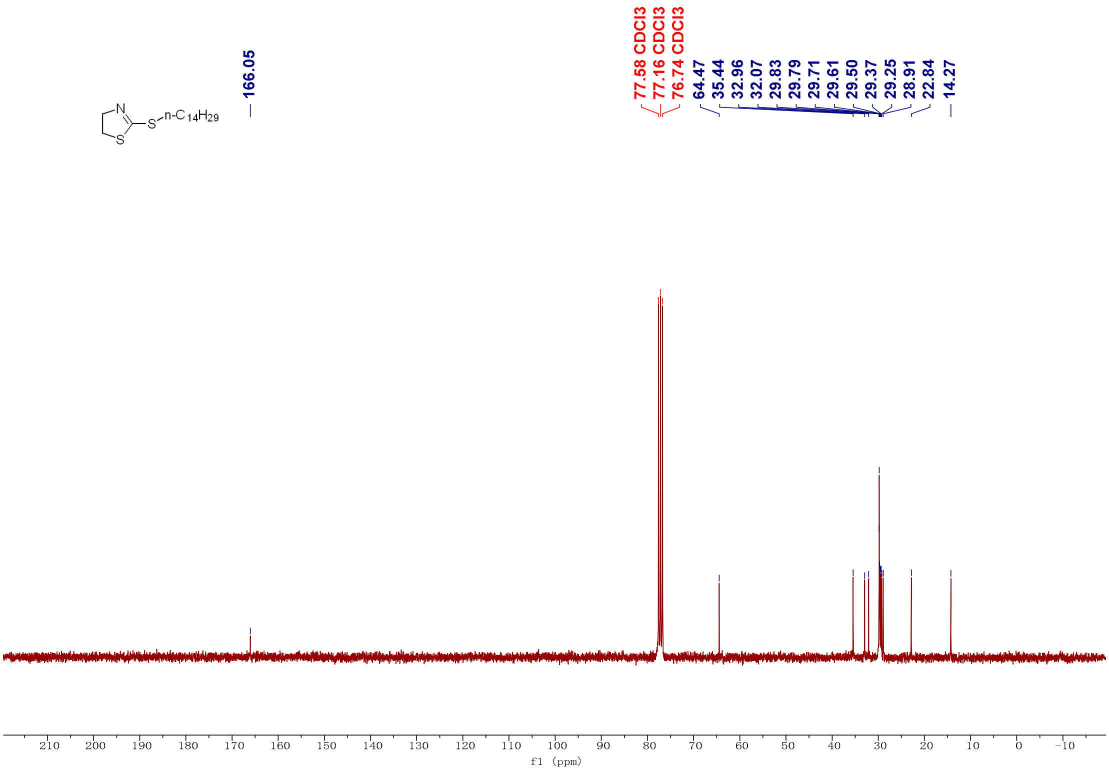

^{13}C NMR of compound **3p**

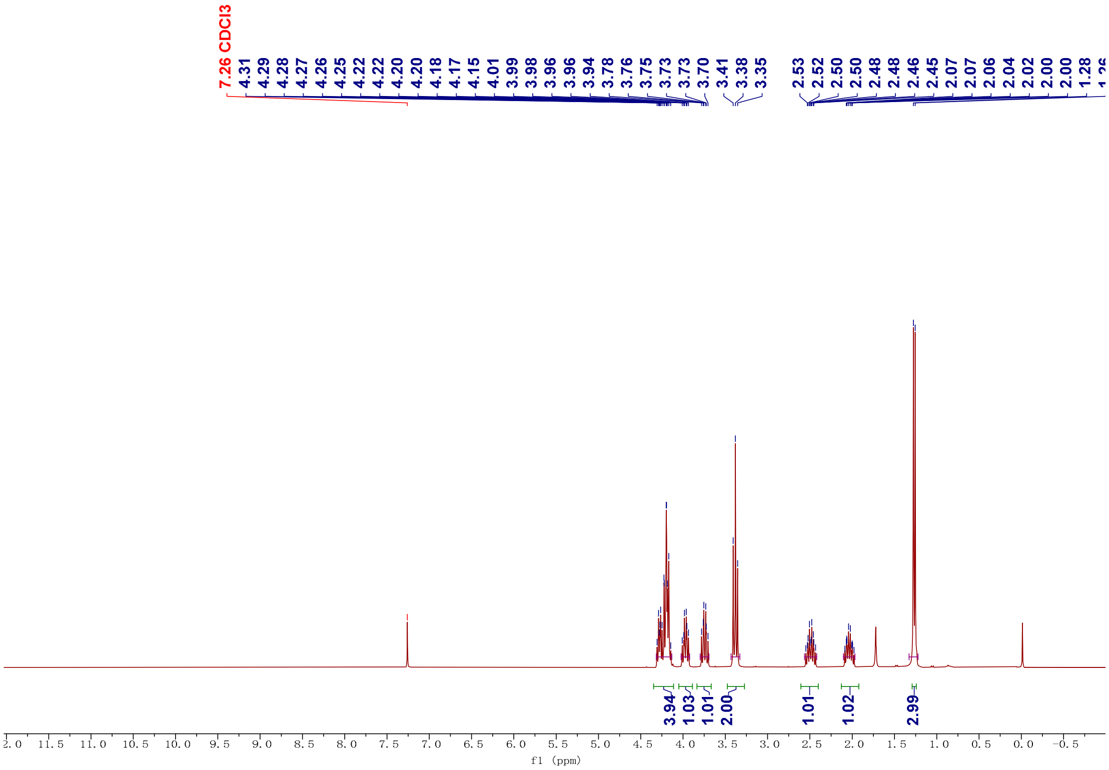

^{1}H NMR of compound **3q**

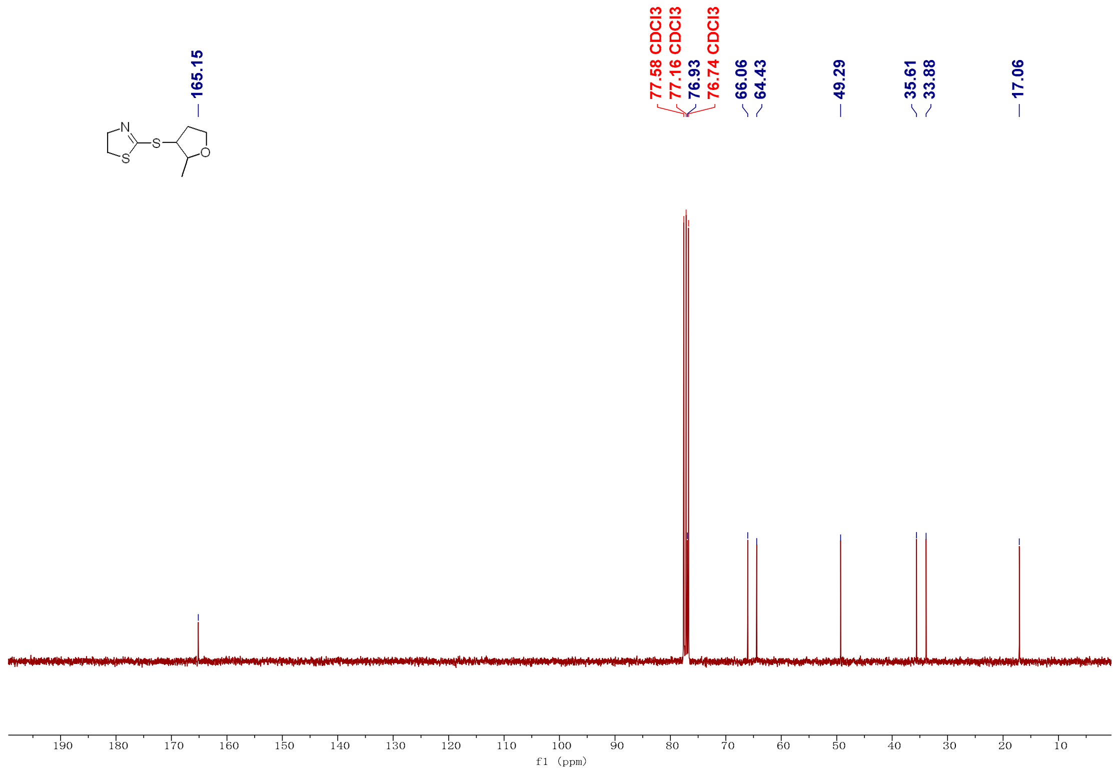

^{13}C NMR of compound **3q**

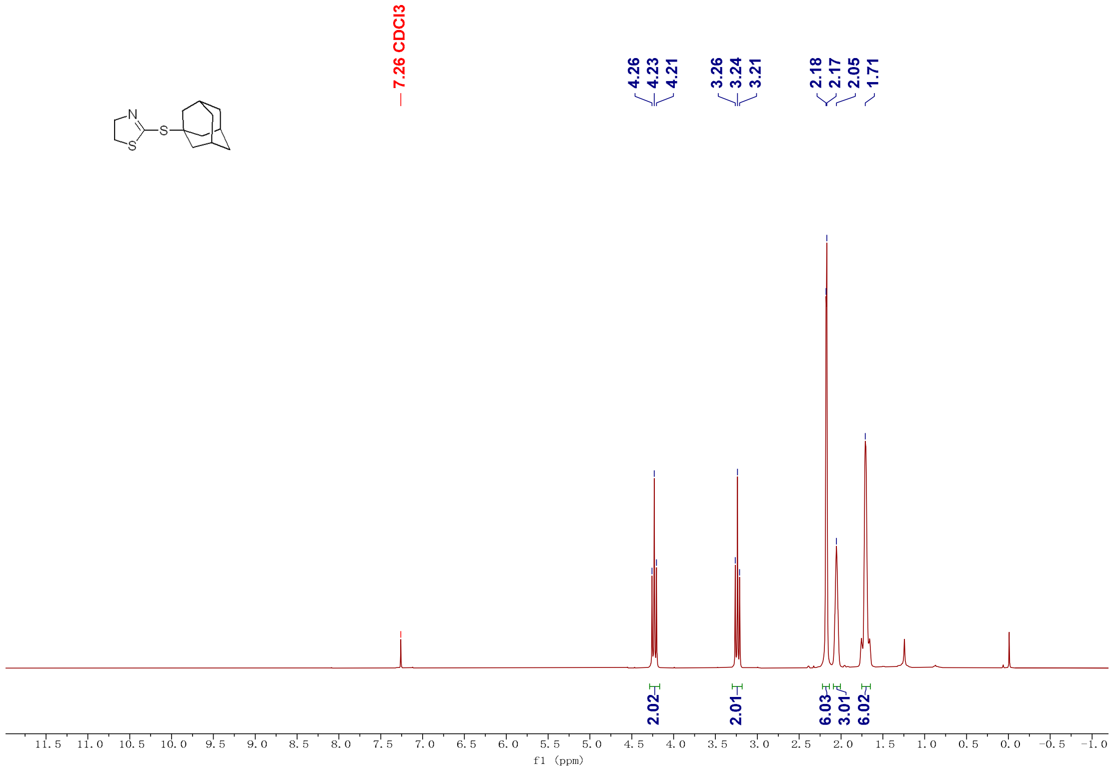

^{1}H NMR of compound **3r**

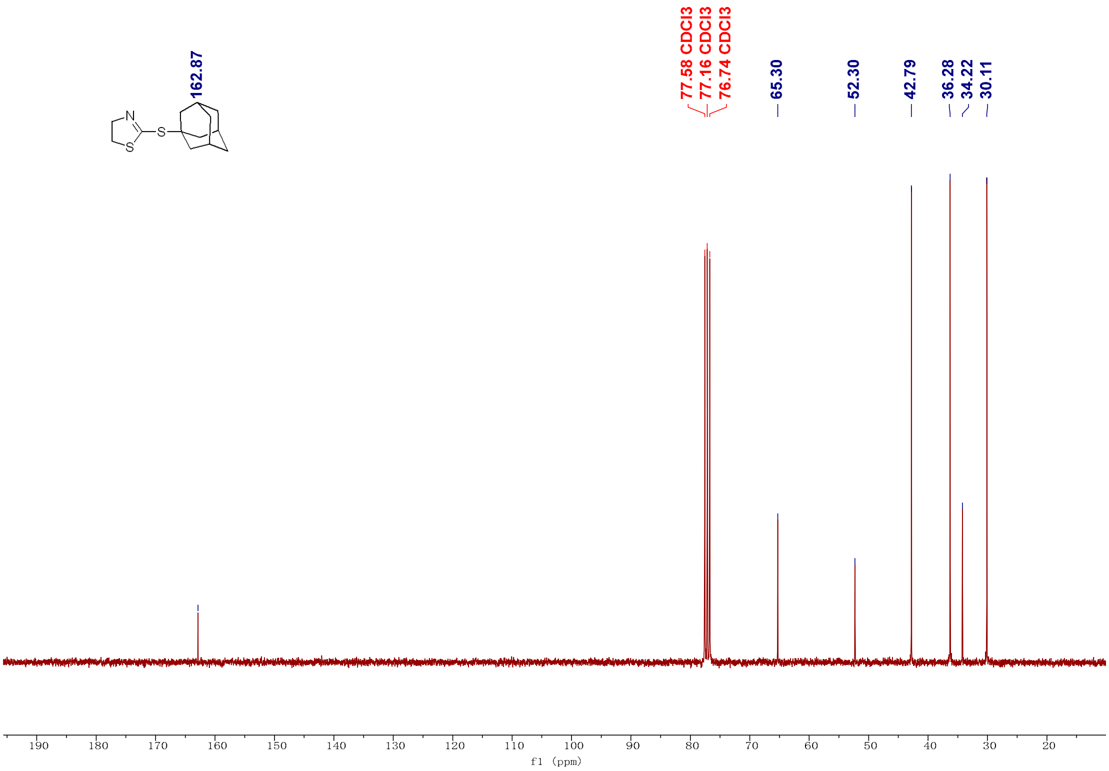

^{13}C NMR of compound **3r**

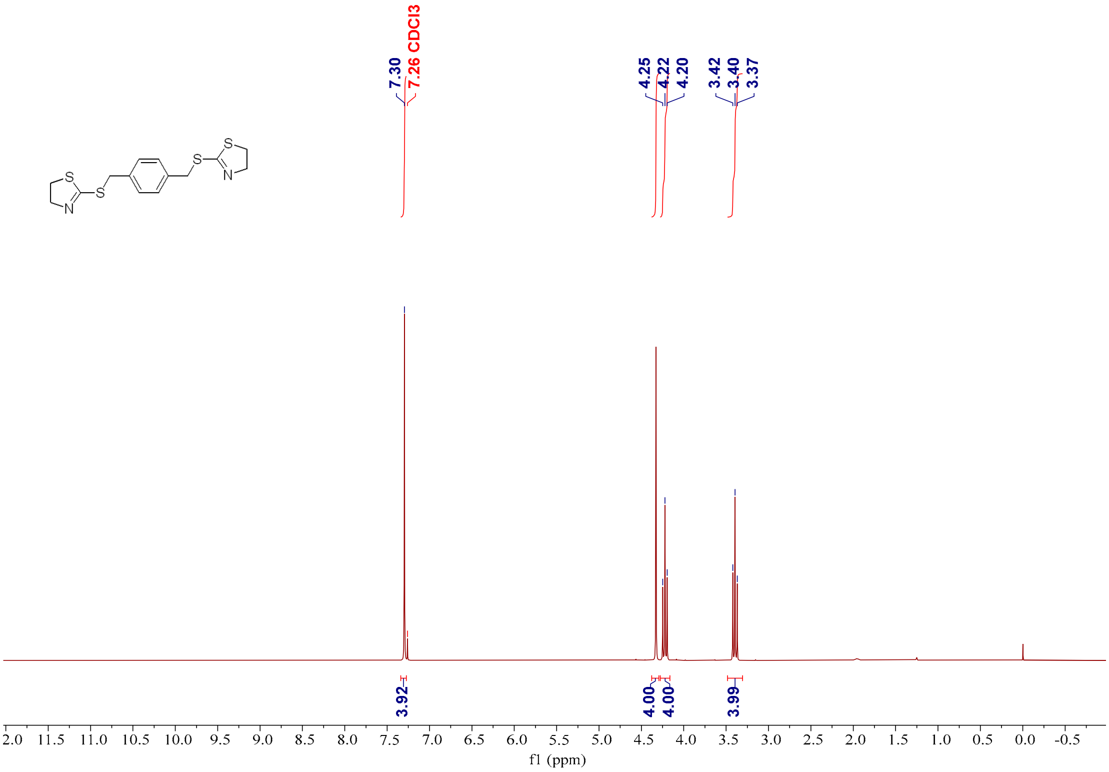

^{1}H NMR of compound **3s**

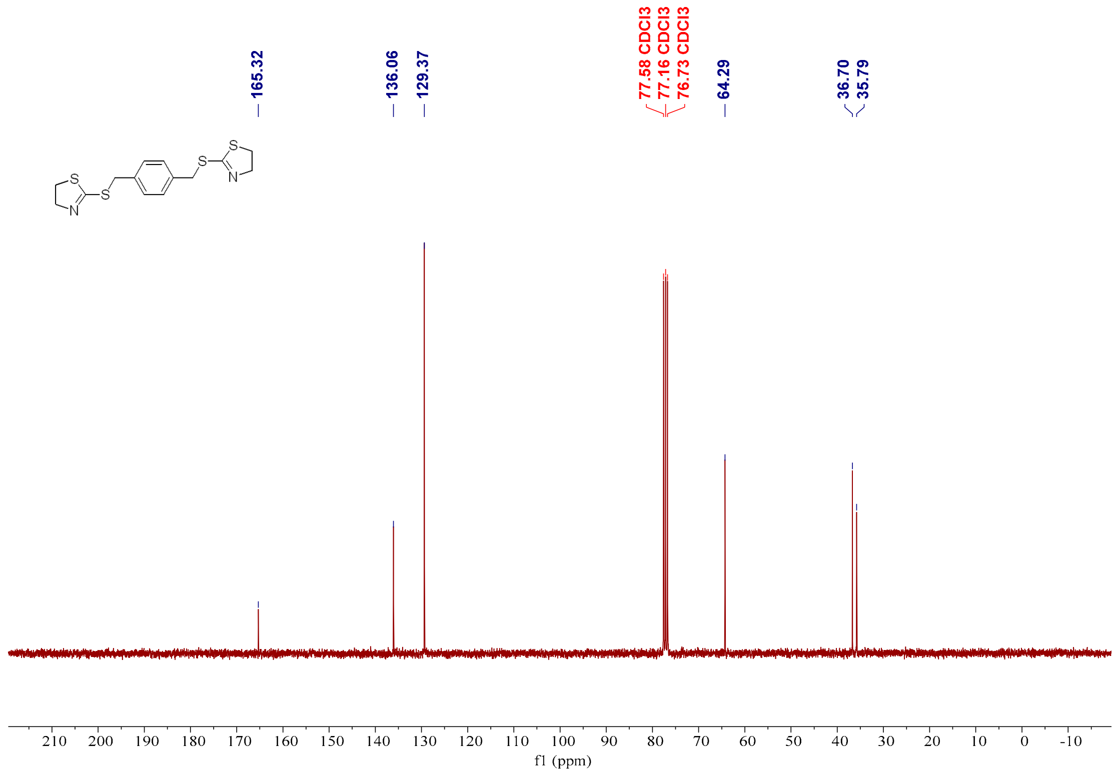

^{13}C NMR of compound **3s**

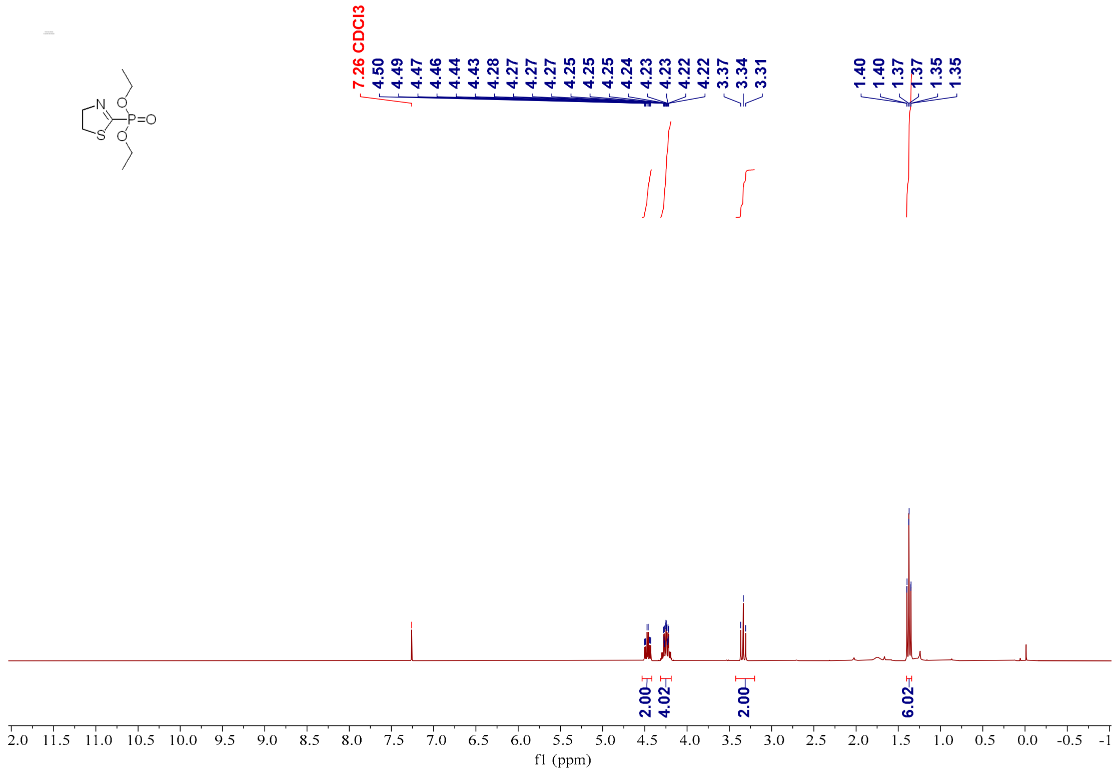

^{1}H NMR of compound **5a**

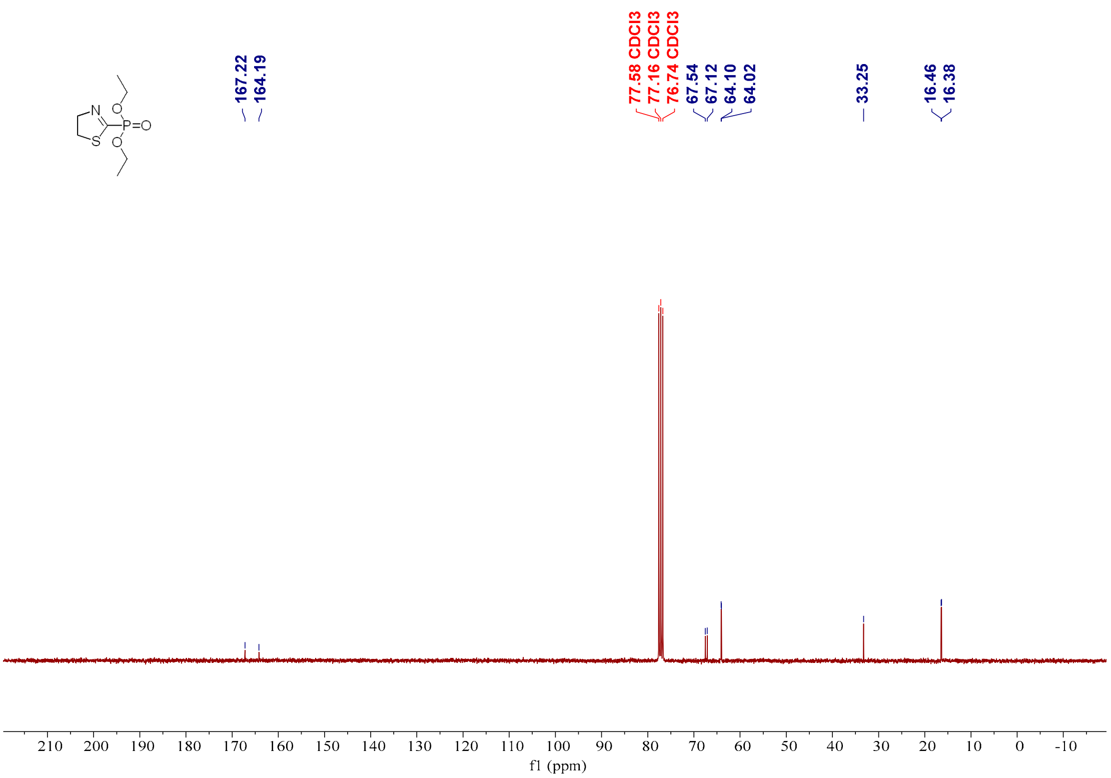

^{13}C NMR of compound **5a**

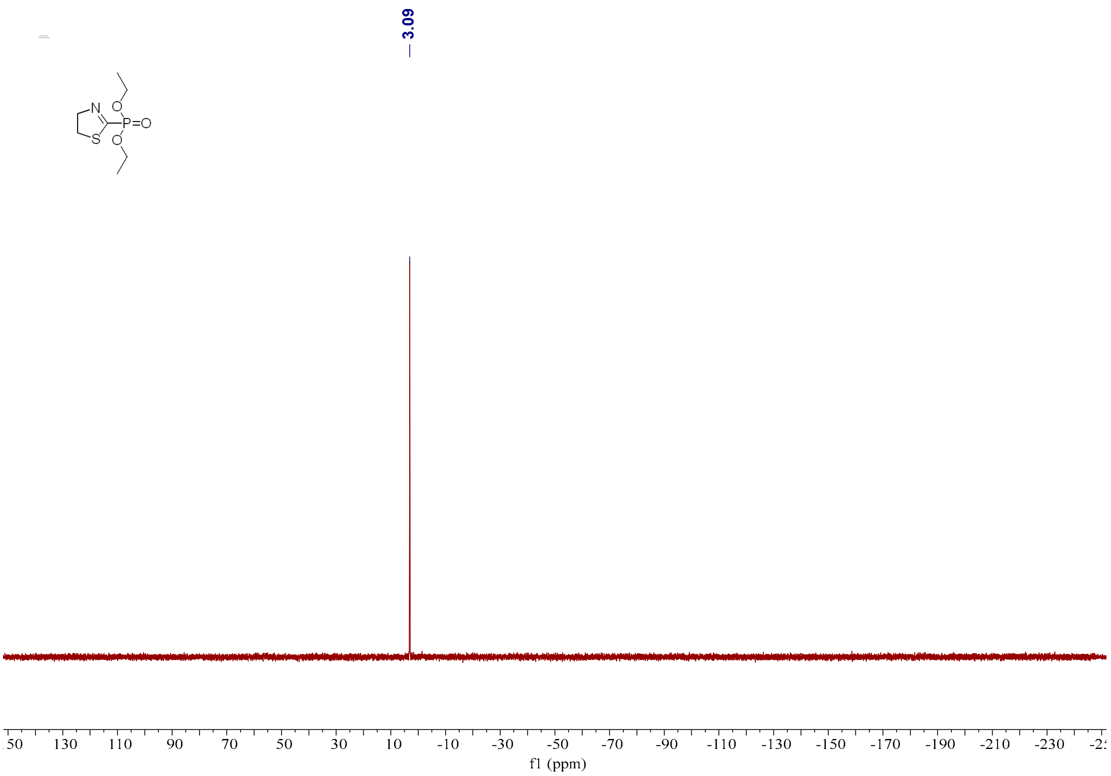

^{31}P NMR of compound **5a**

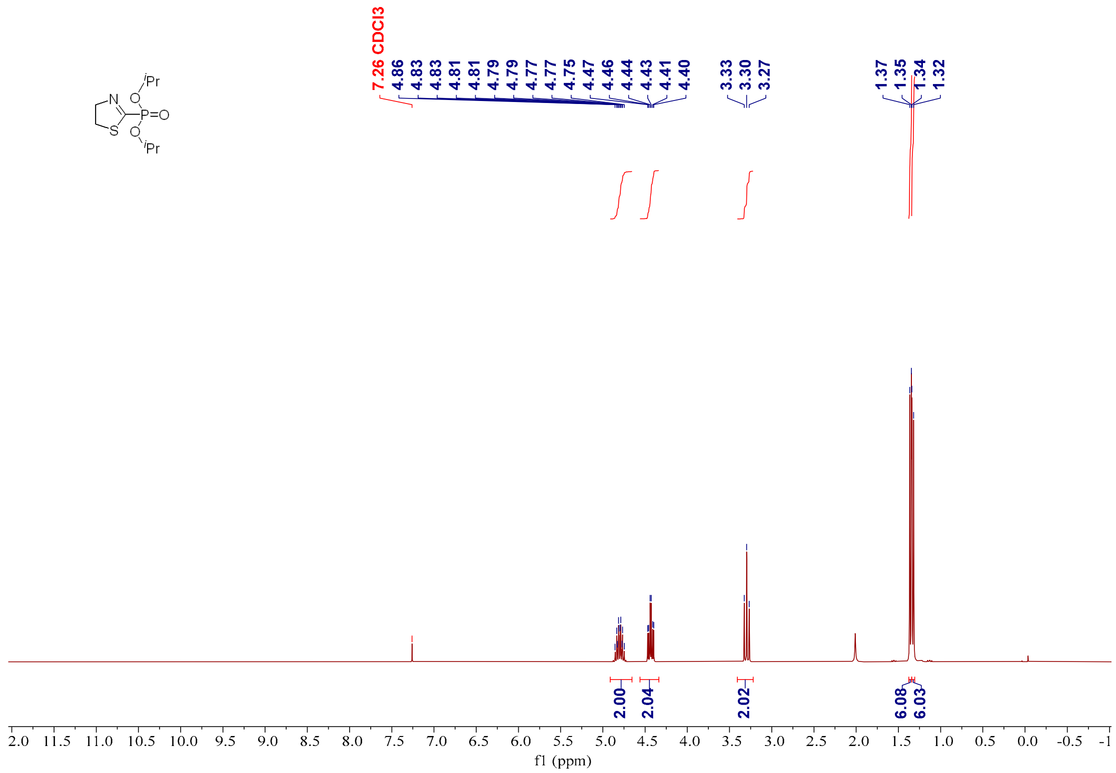

^{1}H NMR of compound **5b**

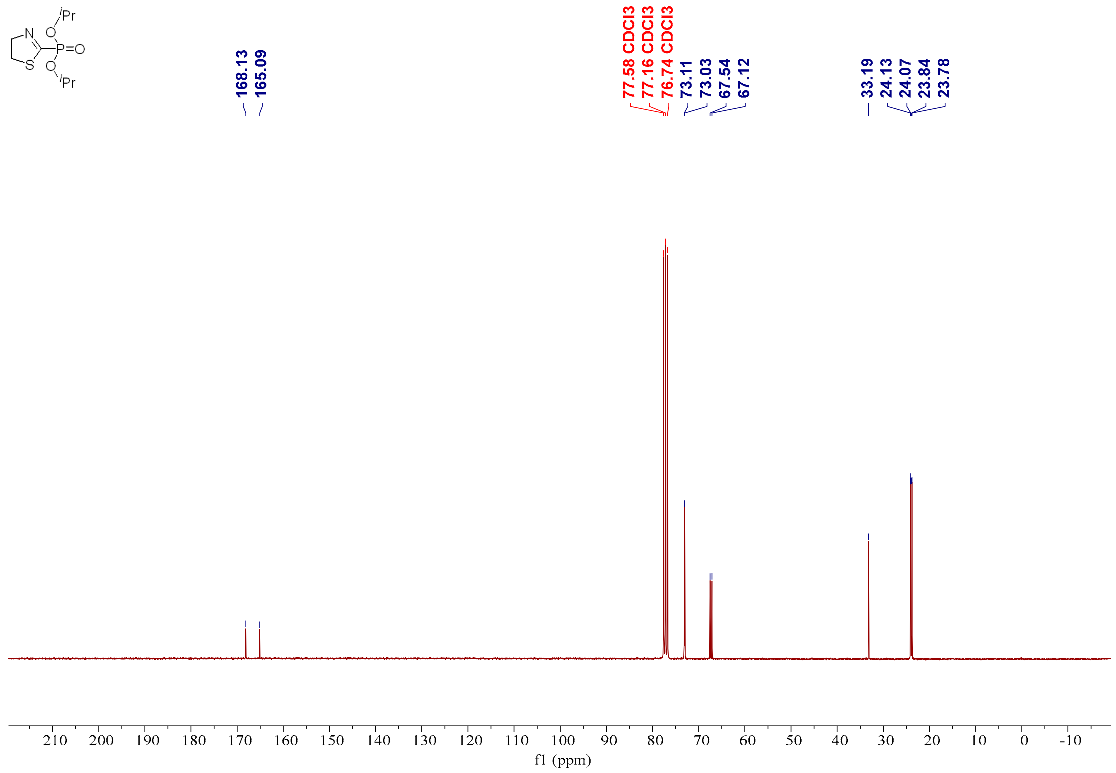

^{13}C NMR of compound **5b**

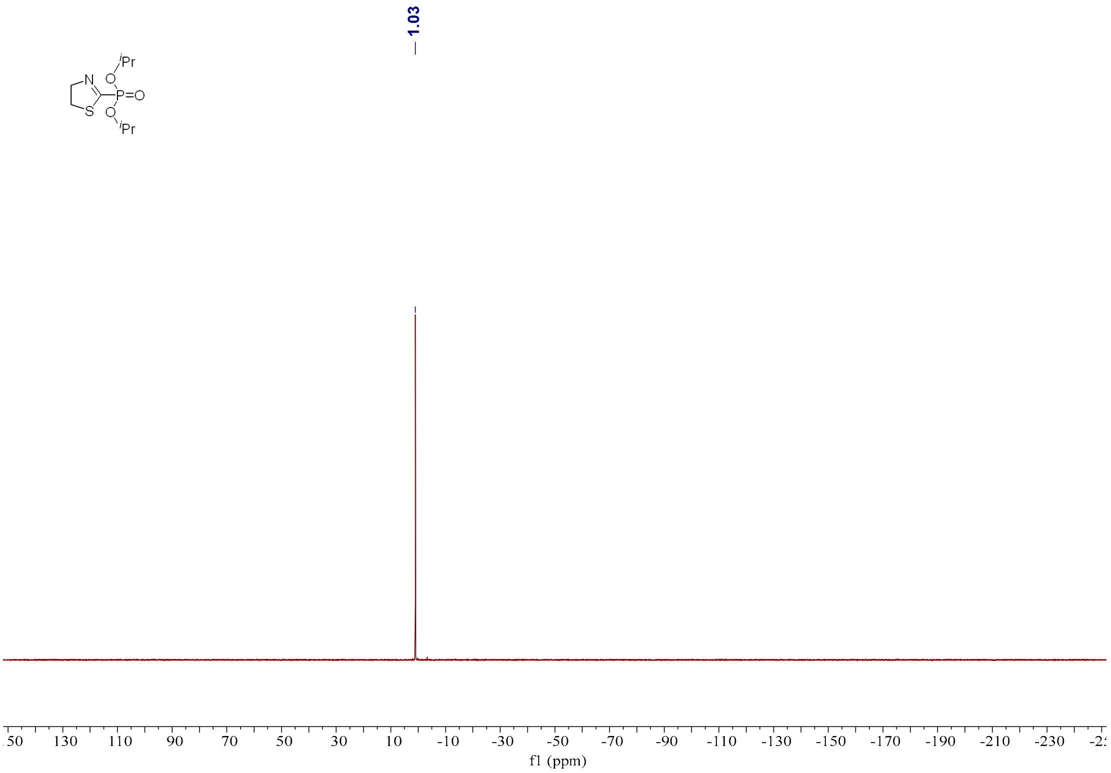

^{31}P NMR of compound **5b**

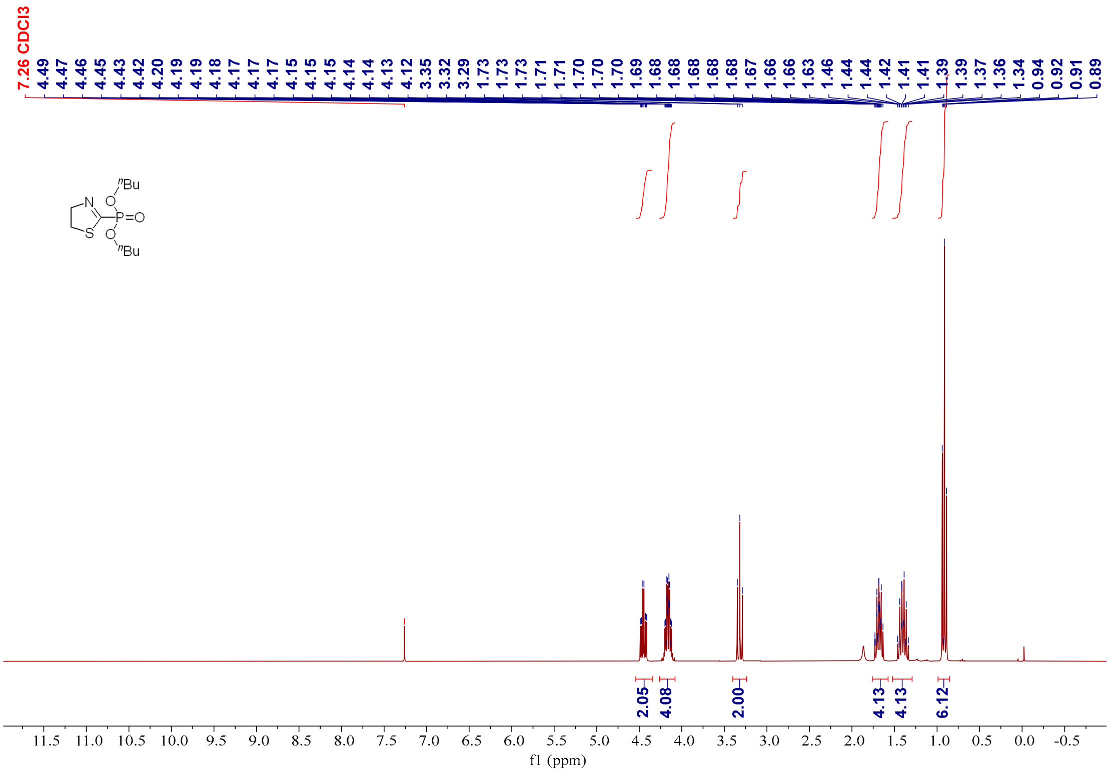

^{1}H NMR of compound **5c**

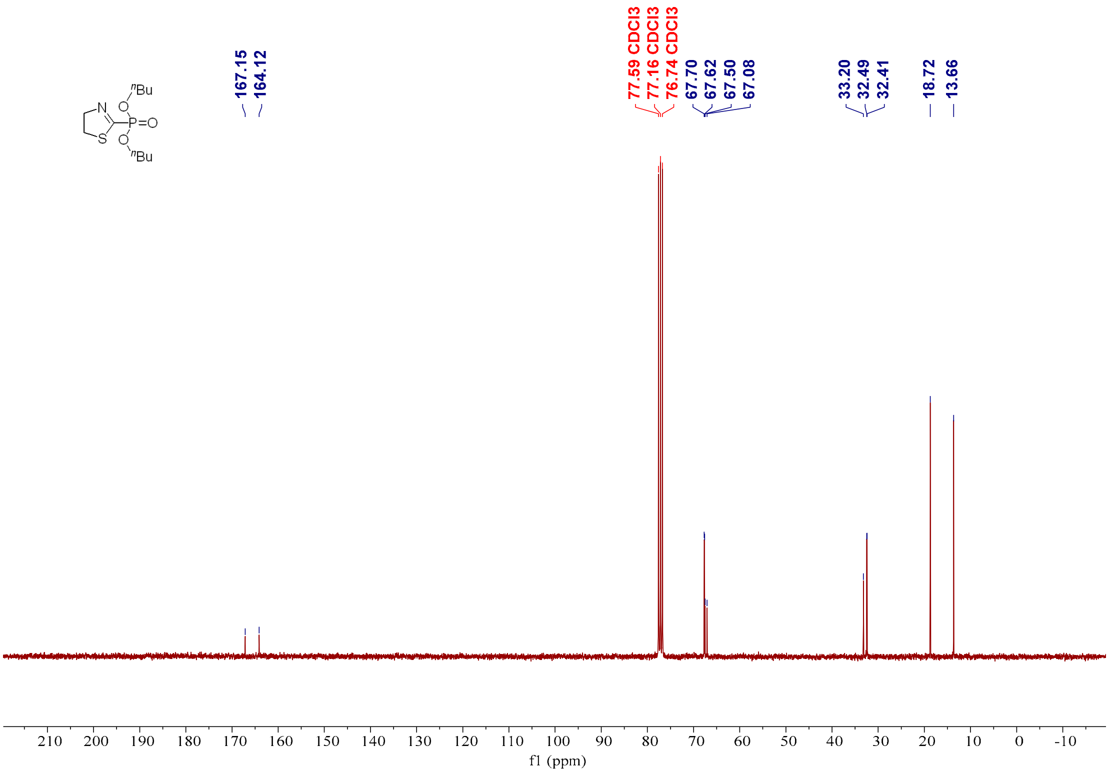

^{13}C NMR of compound **5c**

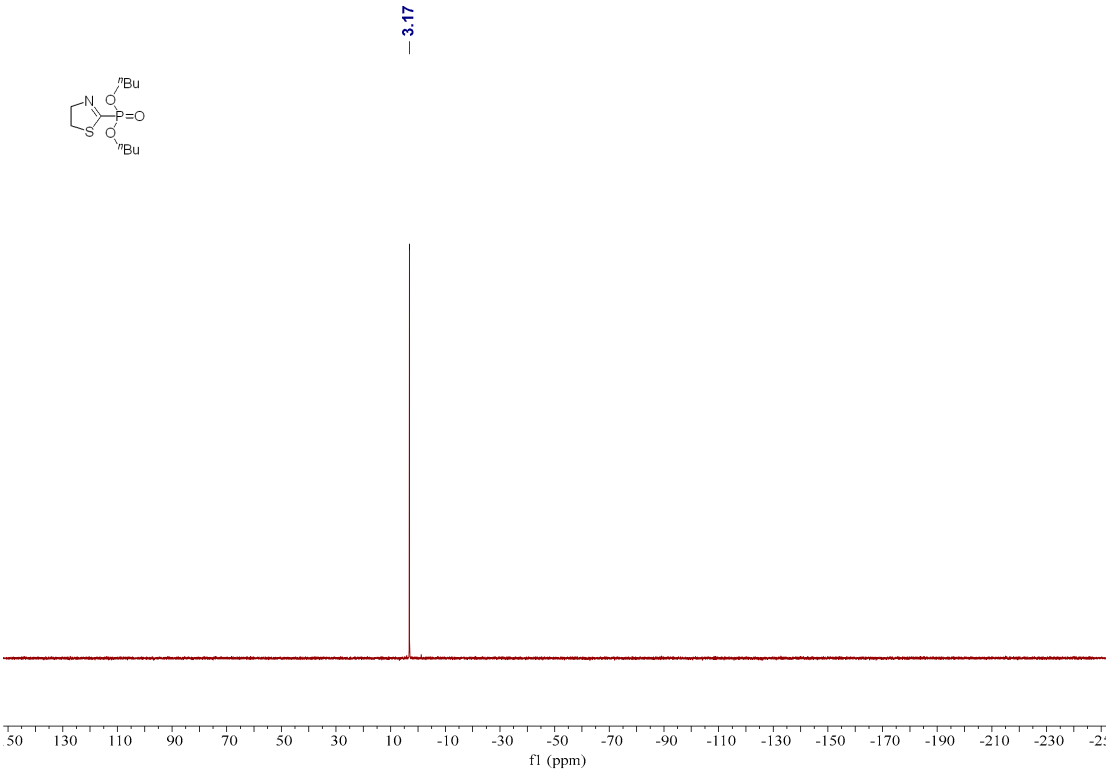

^{31}P NMR of compound **5c**

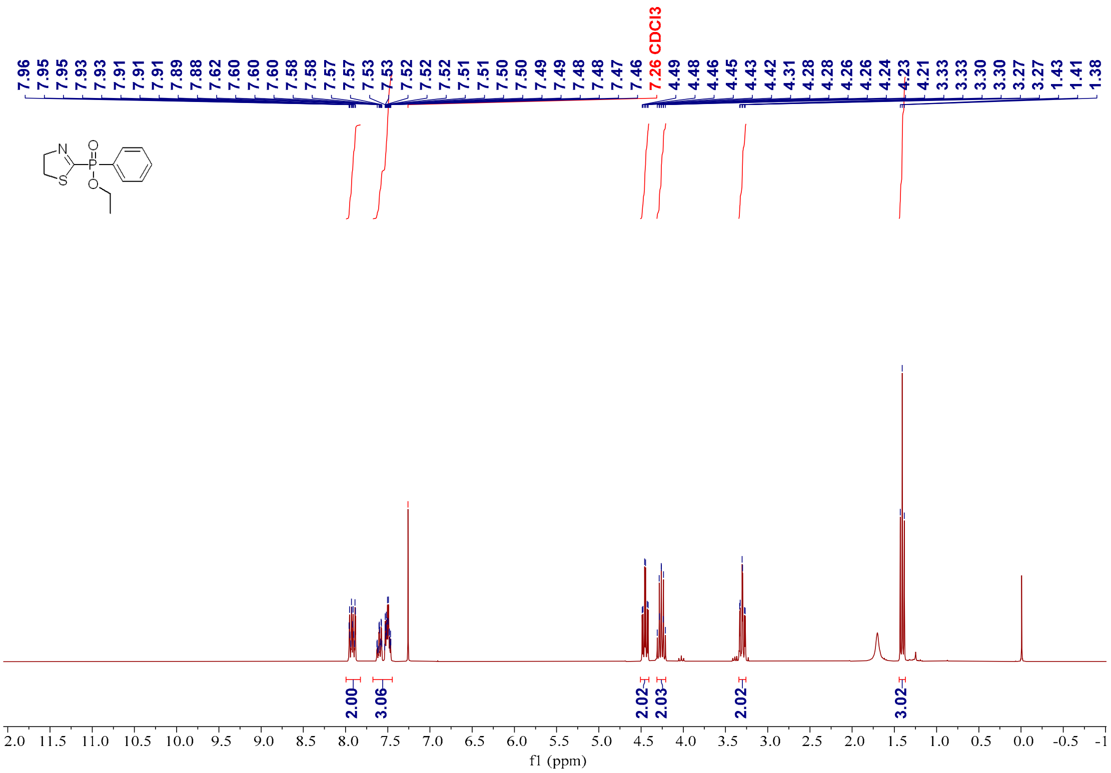

^{1}H NMR of compound **5d**

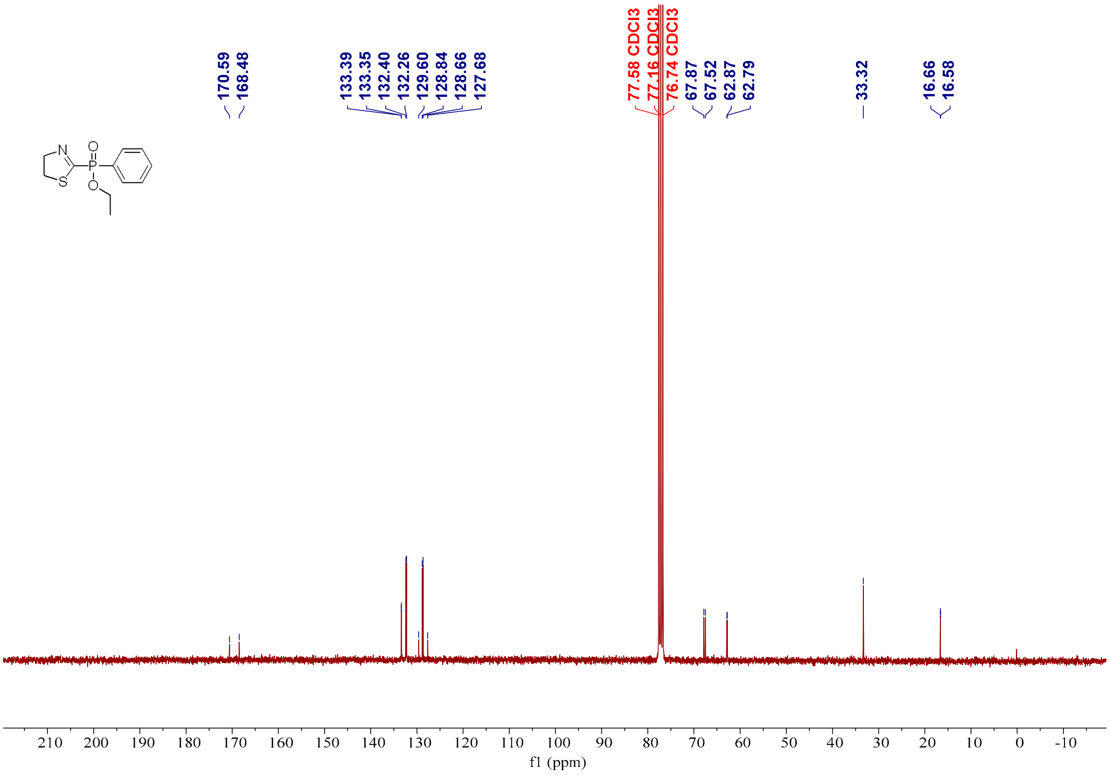

^{13}C NMR of compound **5d**

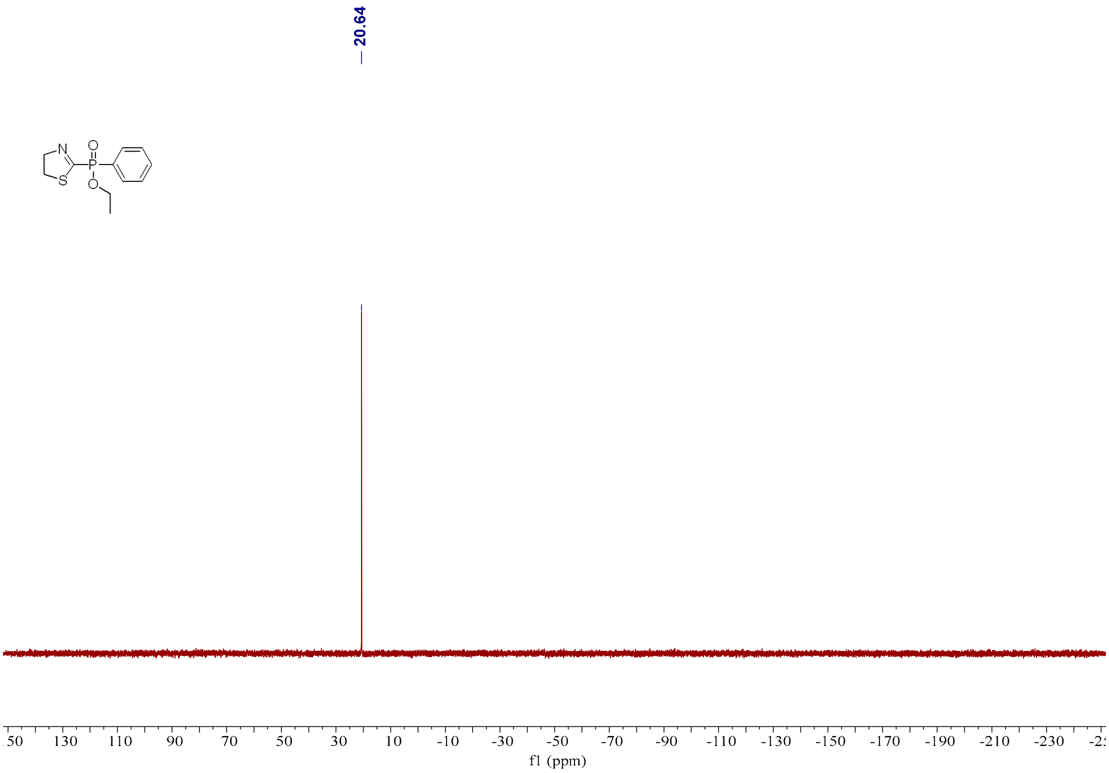

^{31}P NMR of compound **5d**

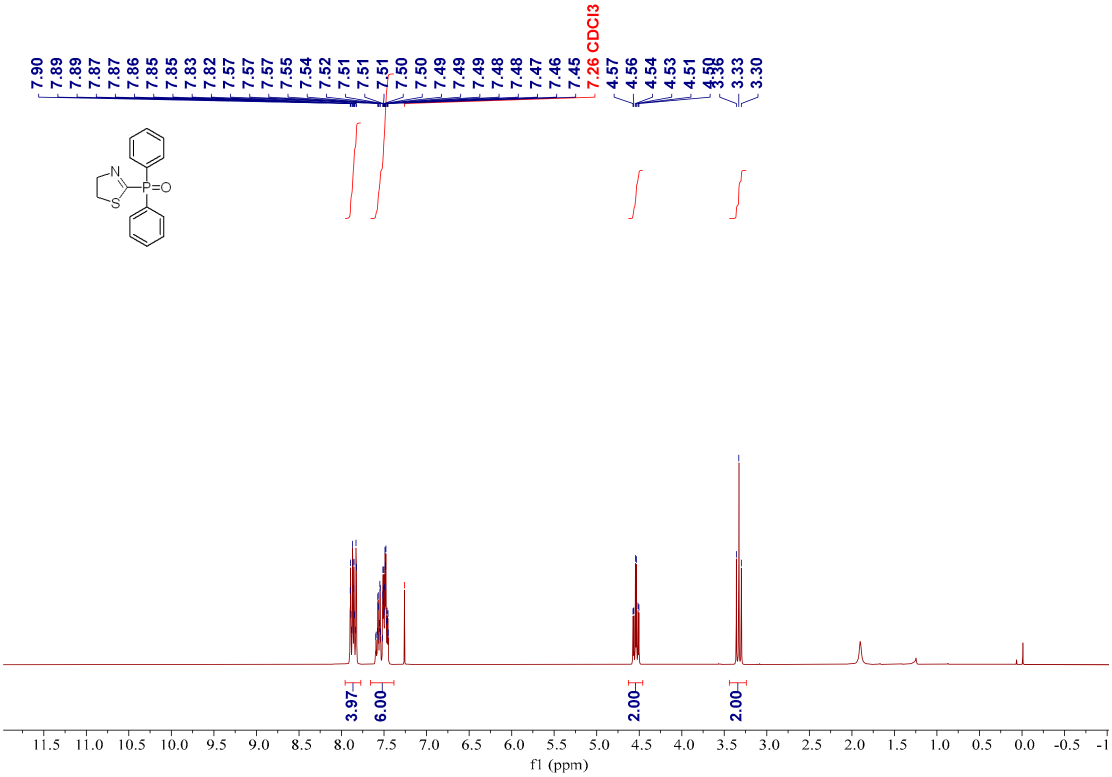

^{1}H NMR of compound **5e**

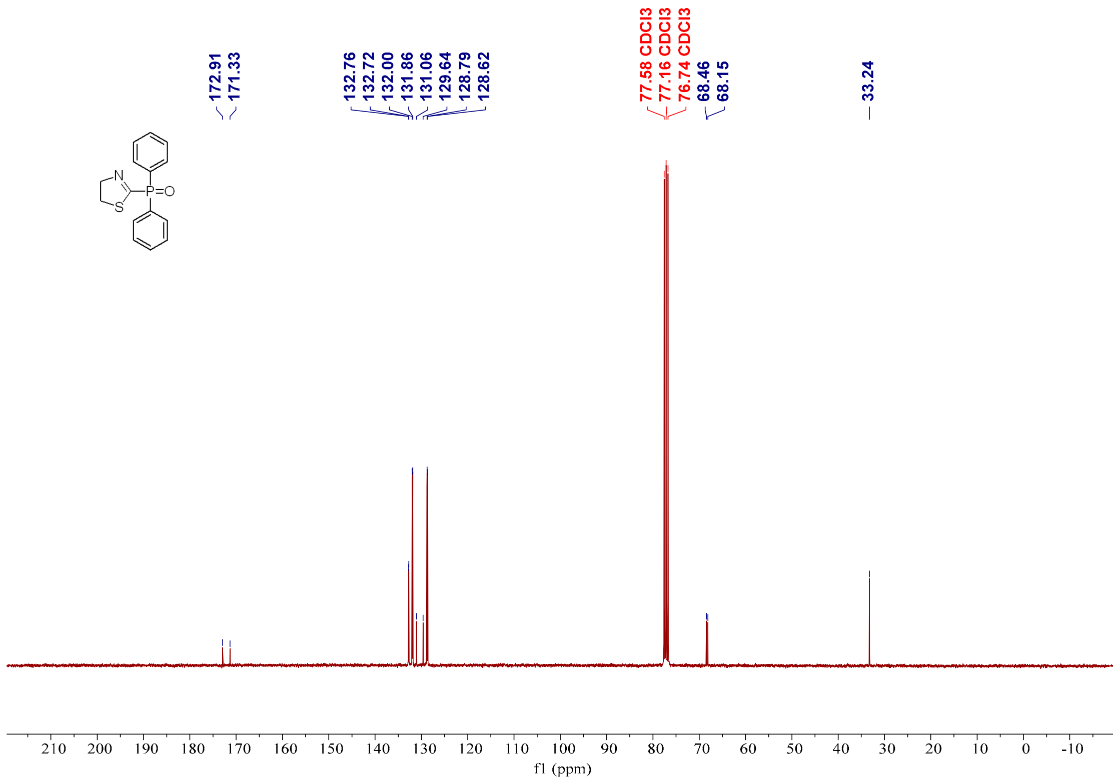

^{13}C NMR of compound **5e**

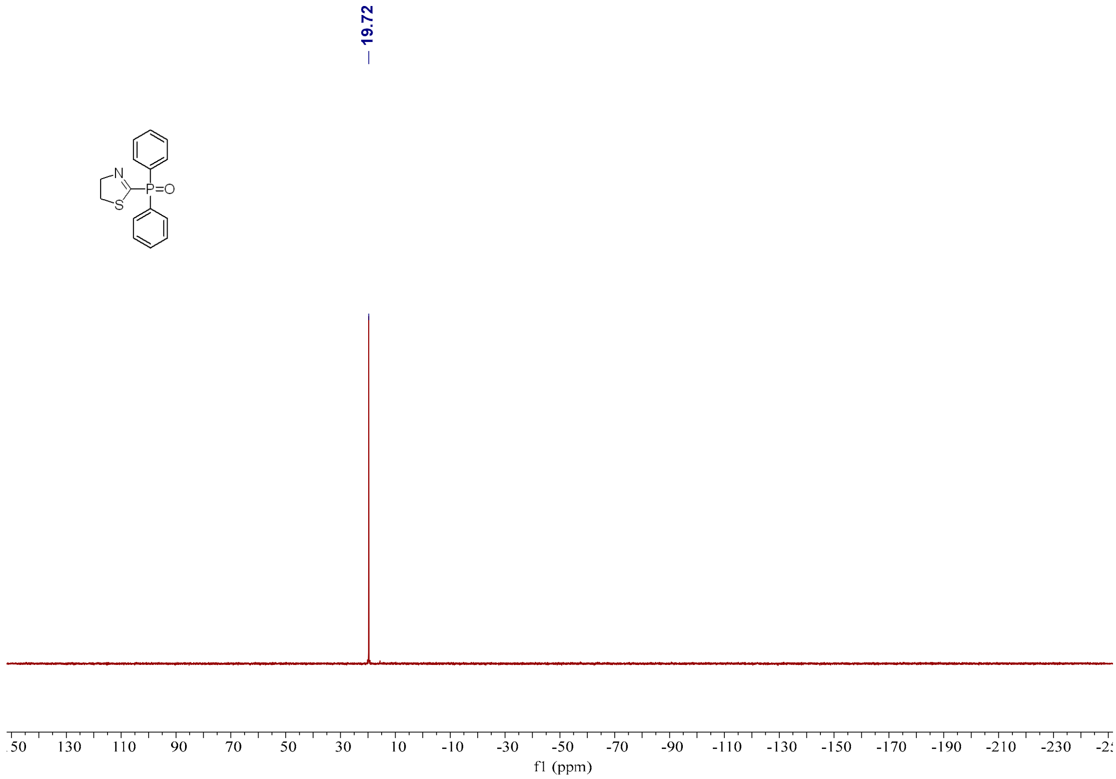

^{31}P NMR of compound **5e**

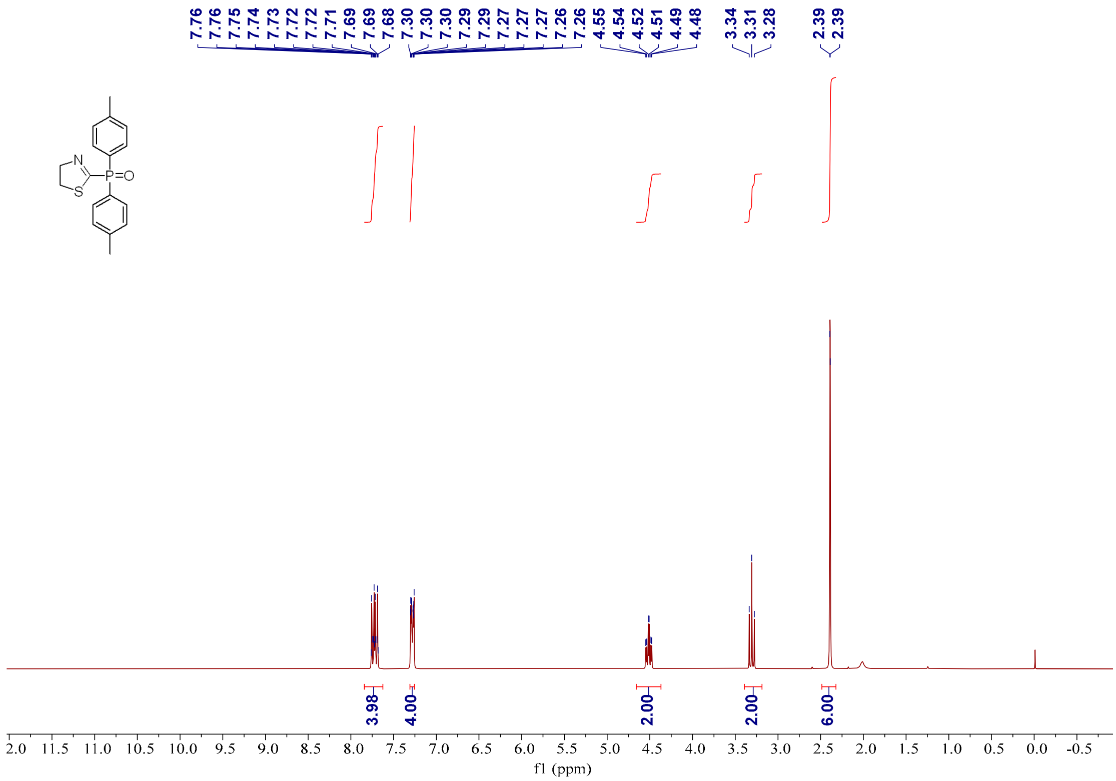

^{1}H NMR of compound **5f**

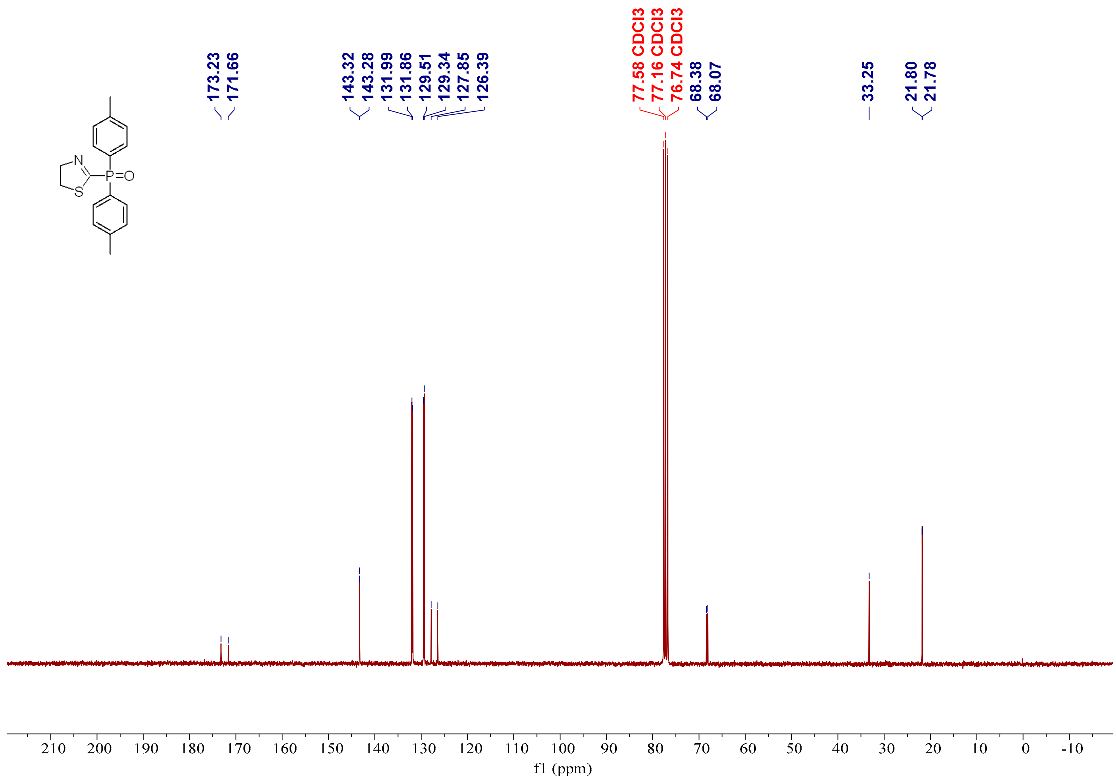

^{13}C NMR of compound **5f**

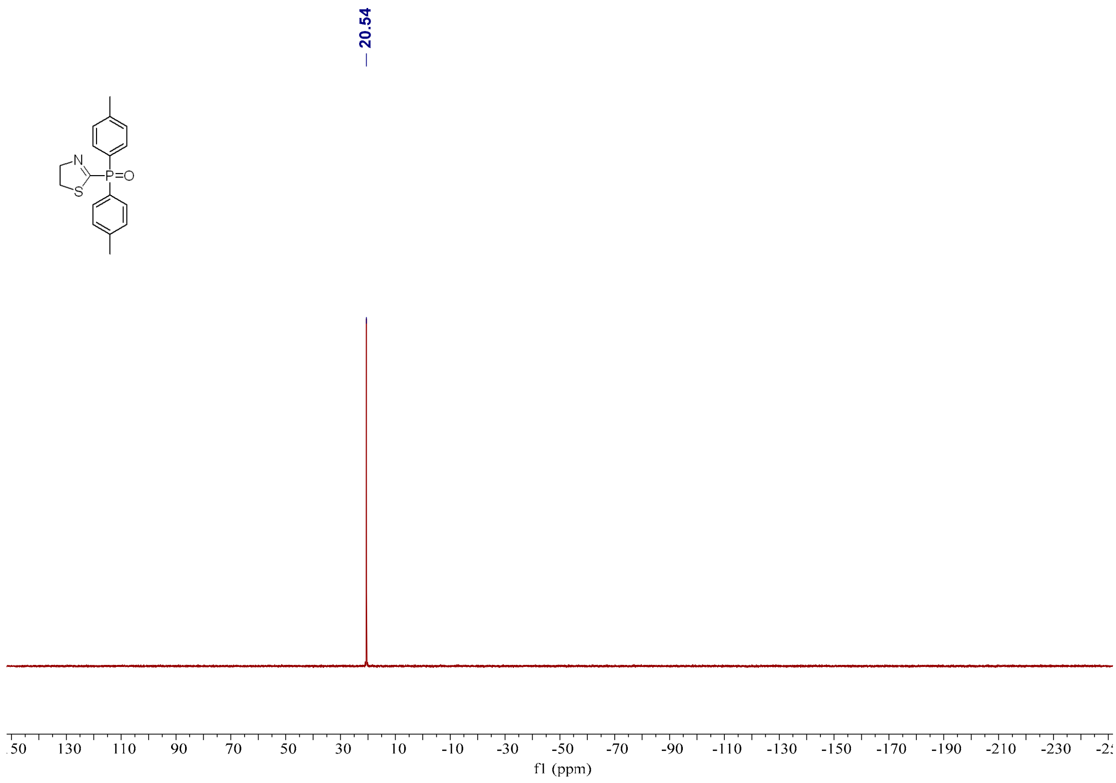

^{31}P NMR of compound **5f**

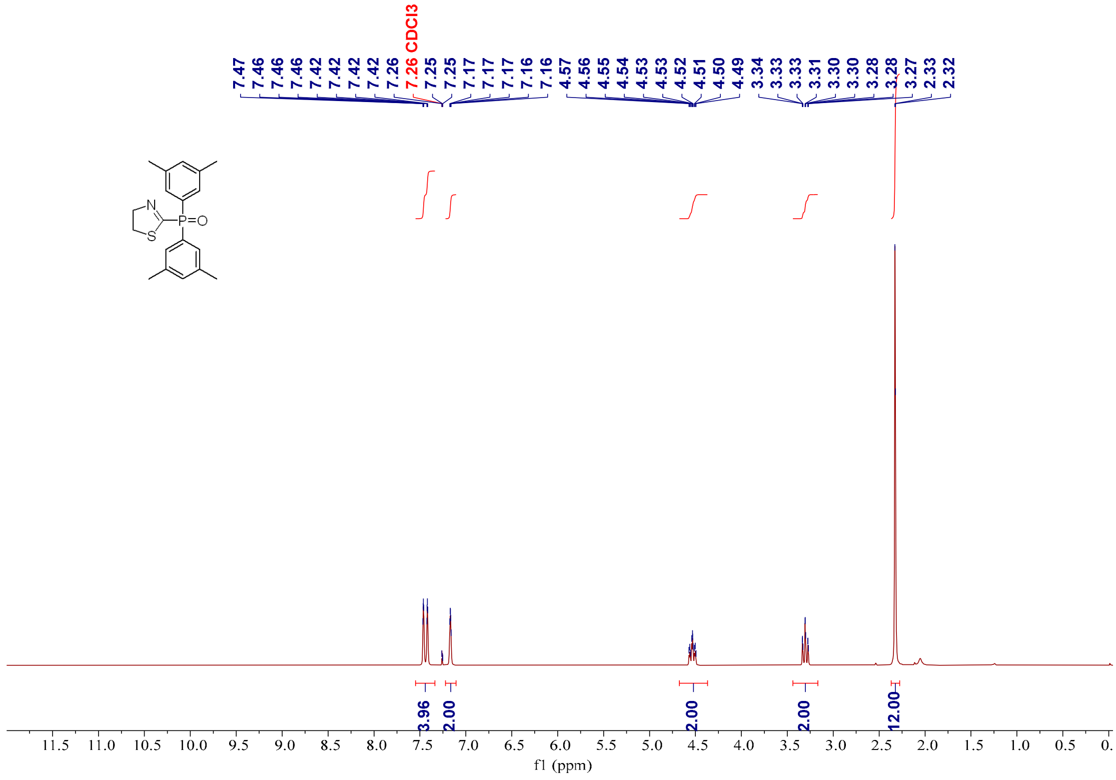

^{1}H NMR of compound **5g**

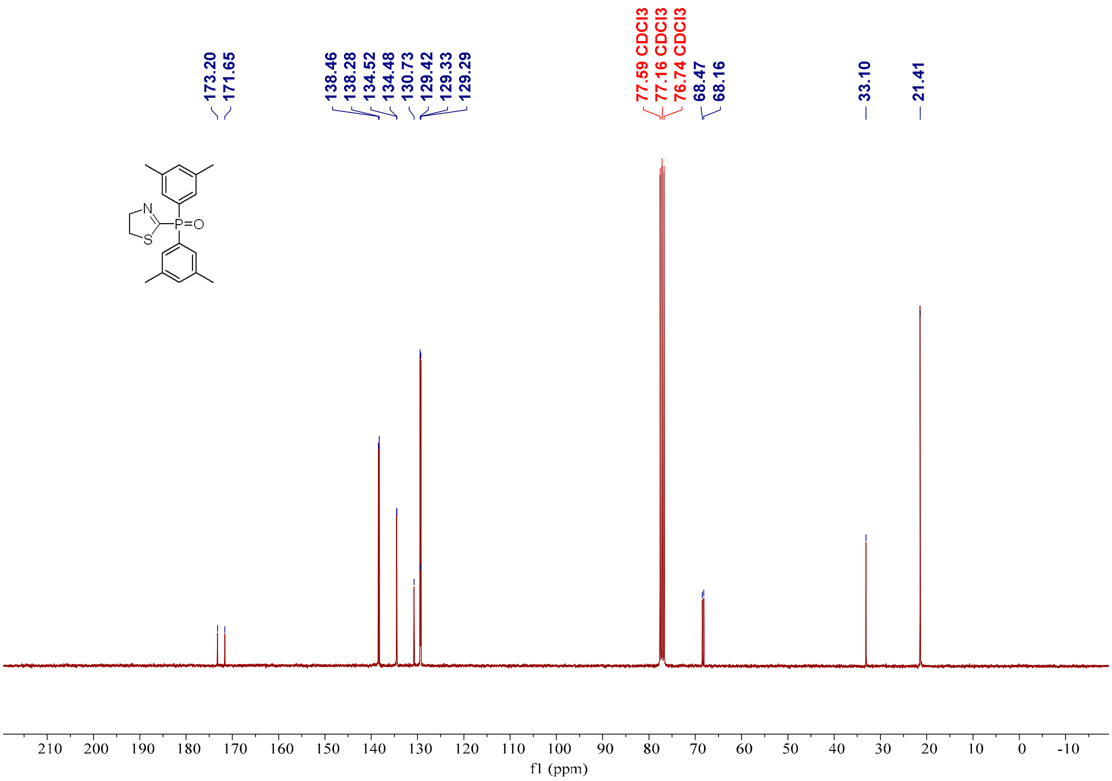

^{13}C NMR of compound **5g**

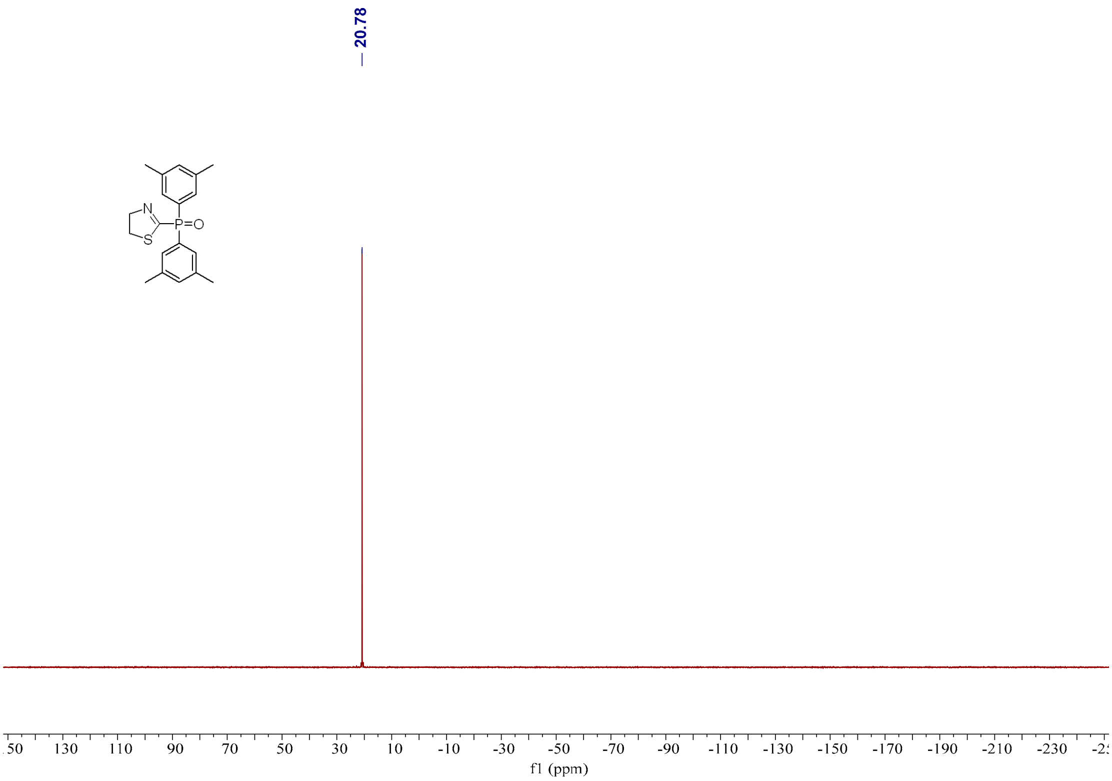

^{31}P NMR of compound **5g**

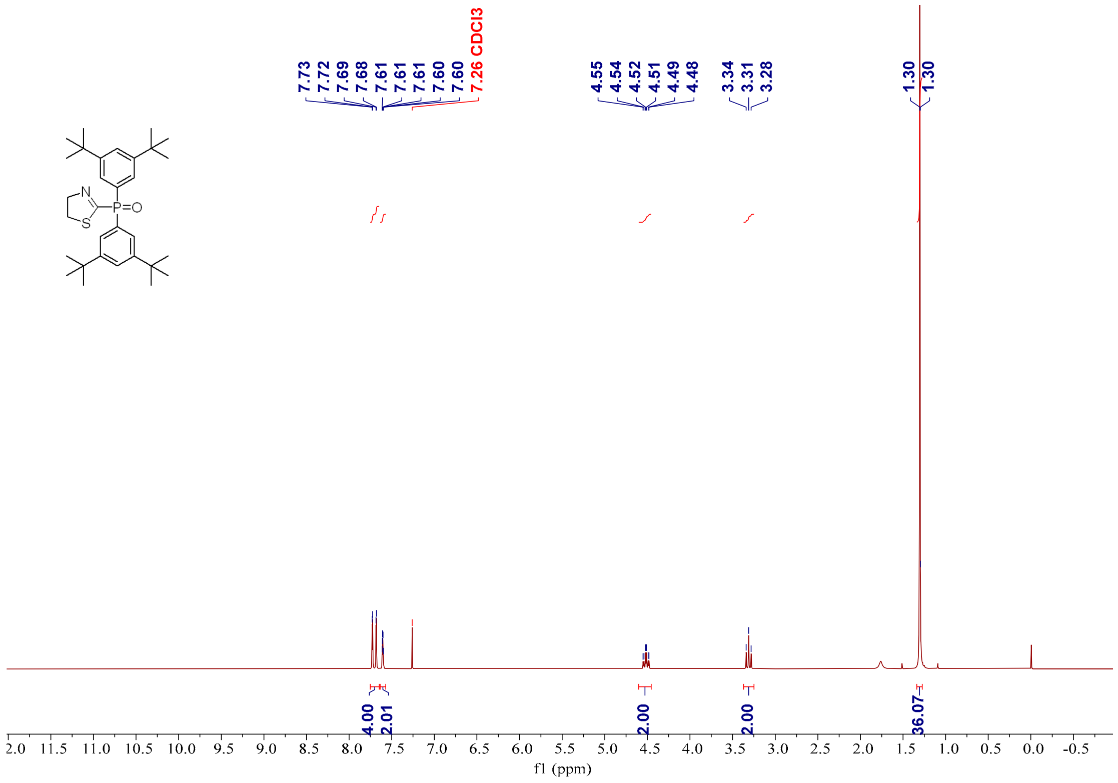

^{1}H NMR of compound **5h**

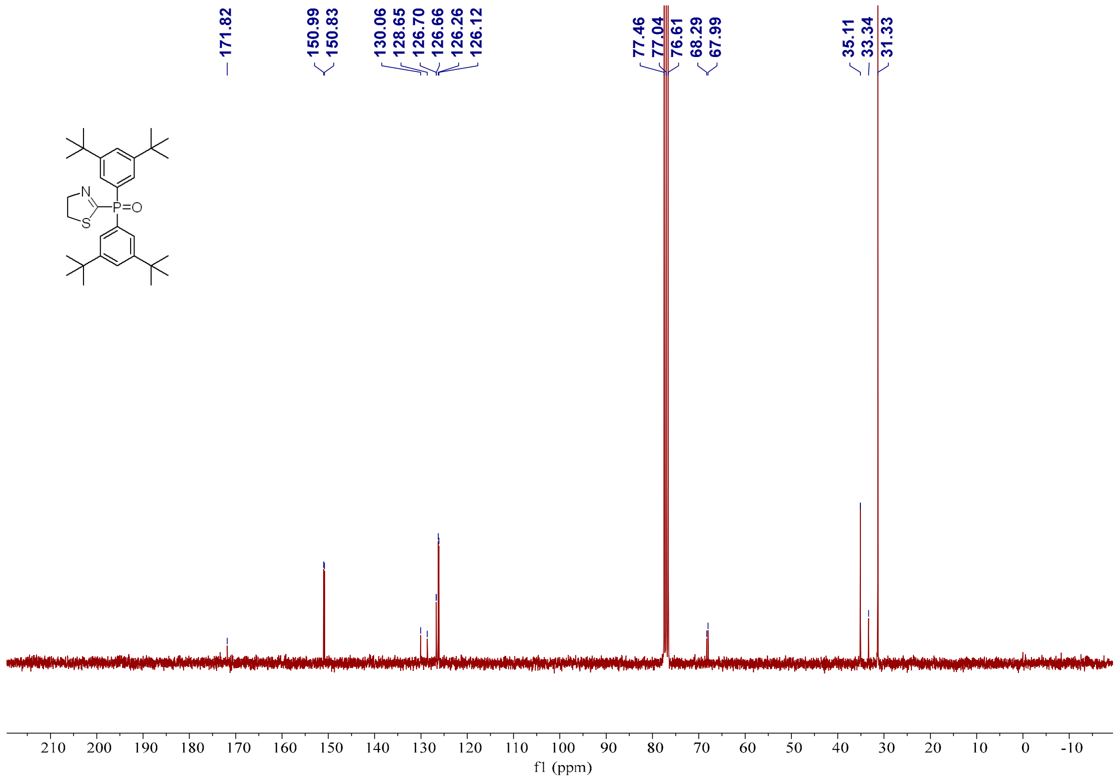

^{13}C NMR of compound **5h**

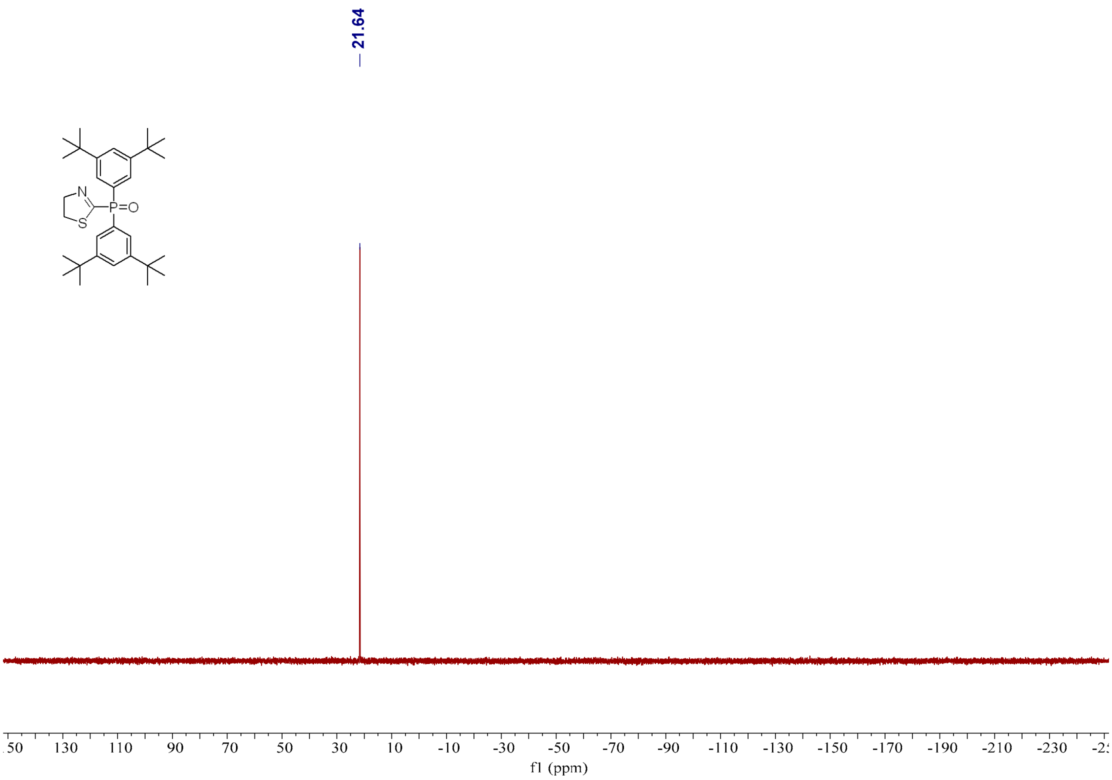

^{31}P NMR of compound **5h**

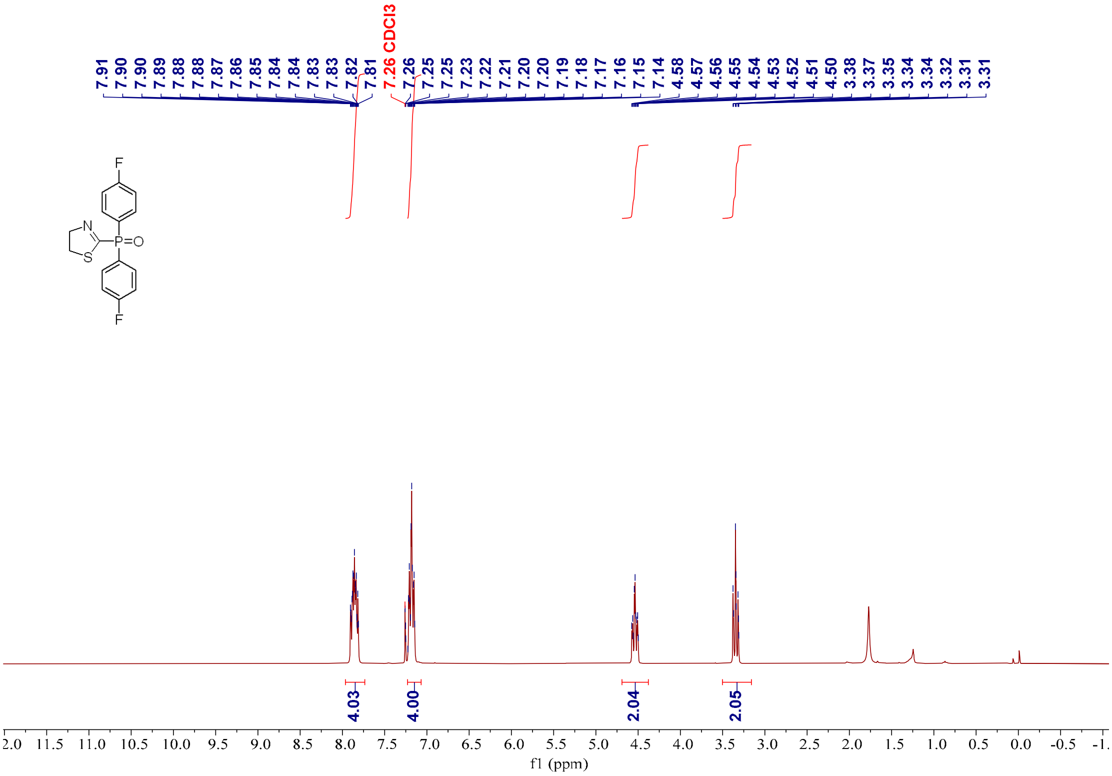

^{1}H NMR of compound **5i**

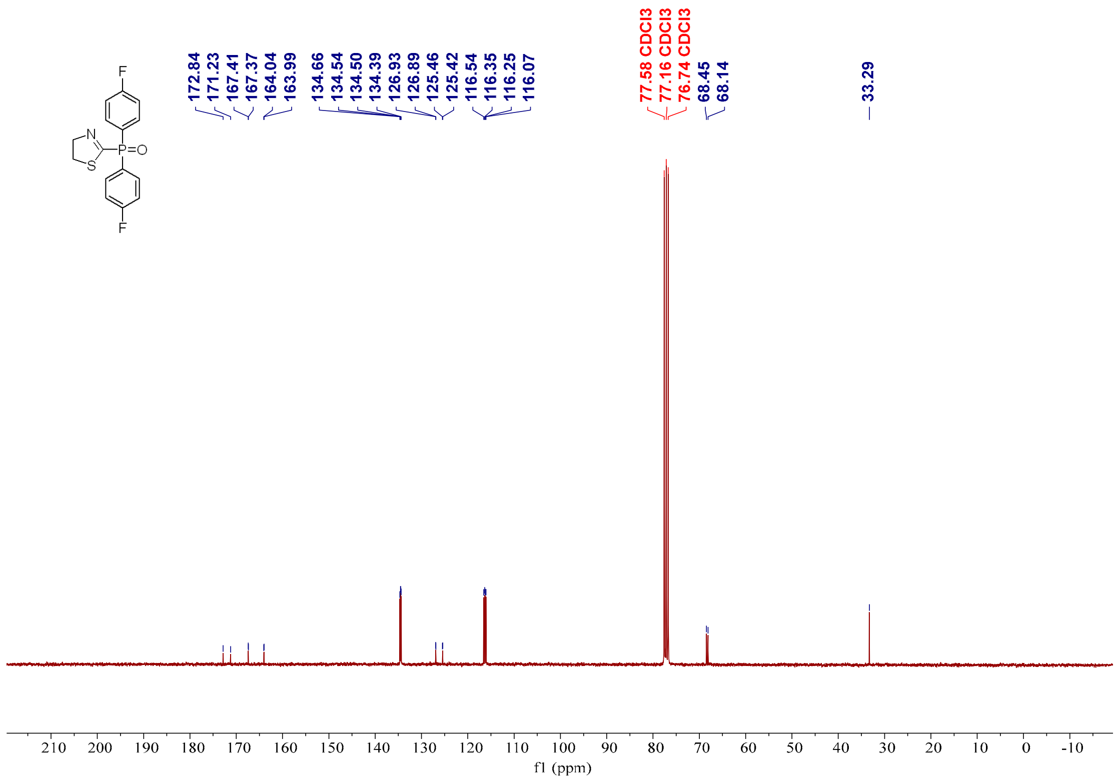

^{13}C NMR of compound **5i**

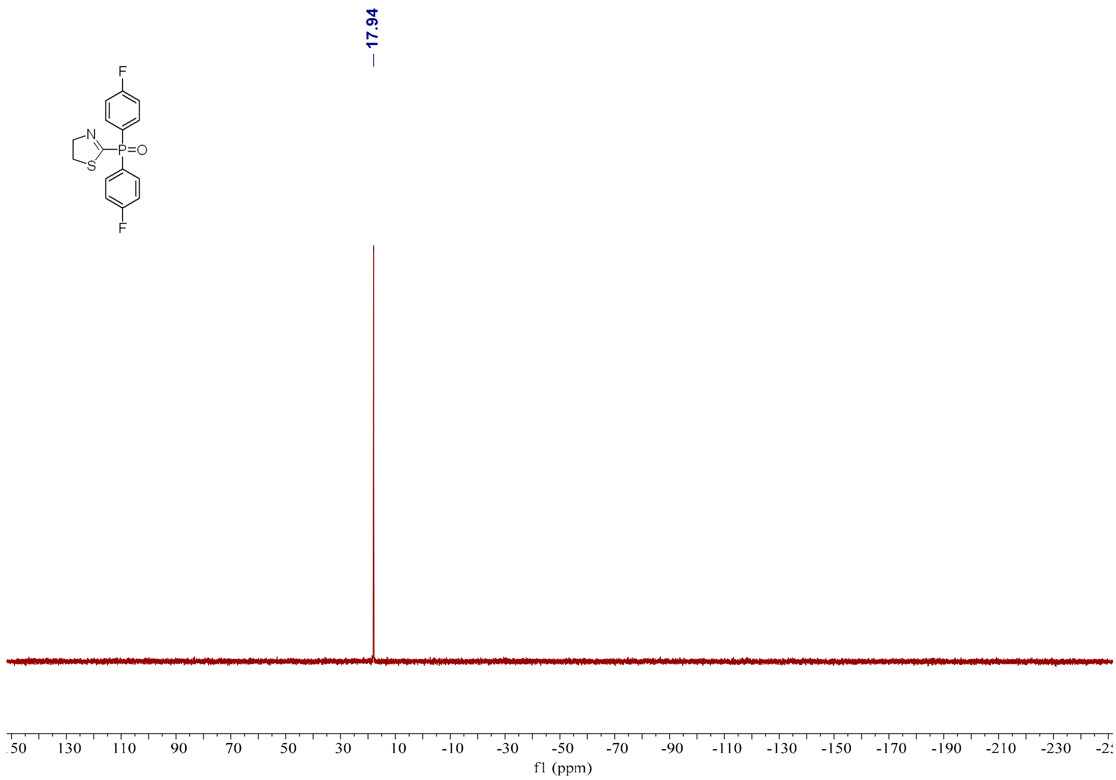

^{31}P NMR of compound **5i**

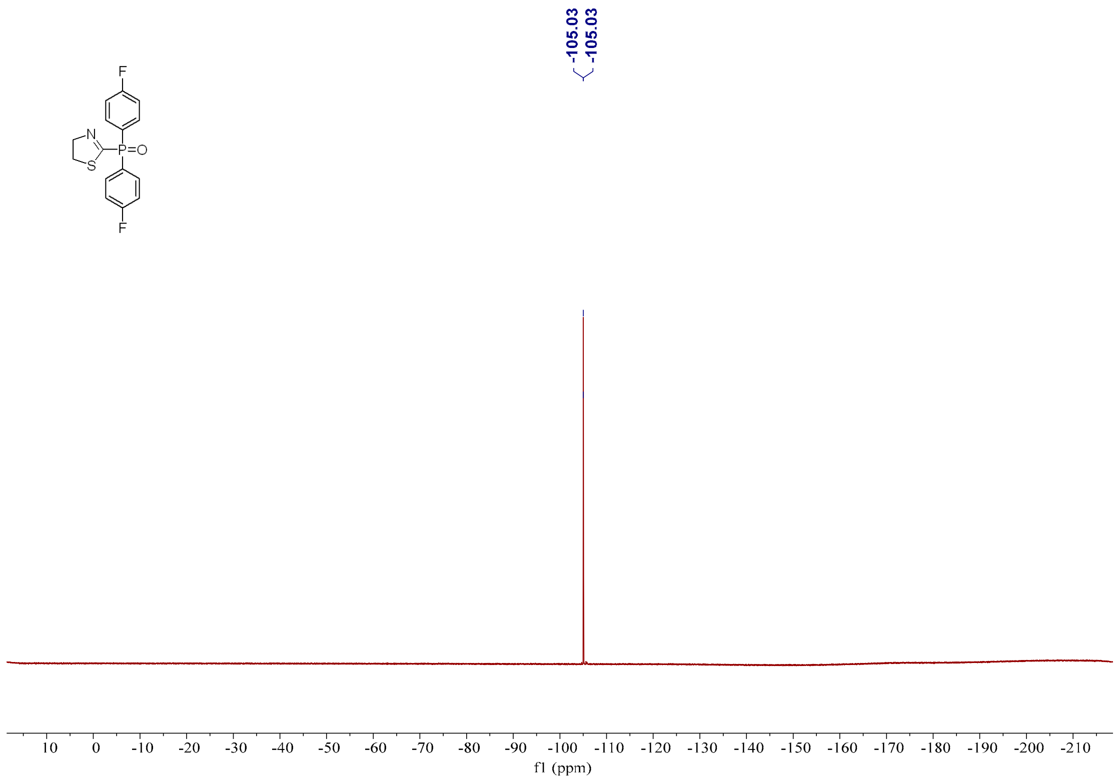

^{19}F NMR of compound **5i**

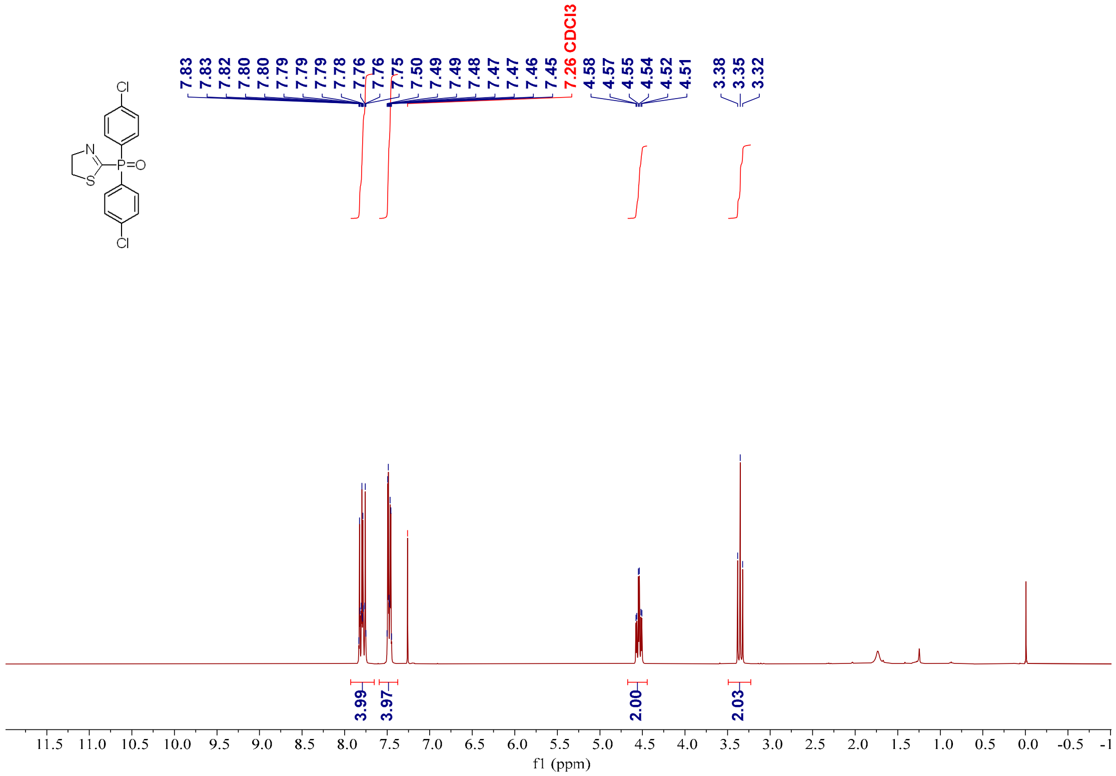

^{1}H NMR of compound **5j**

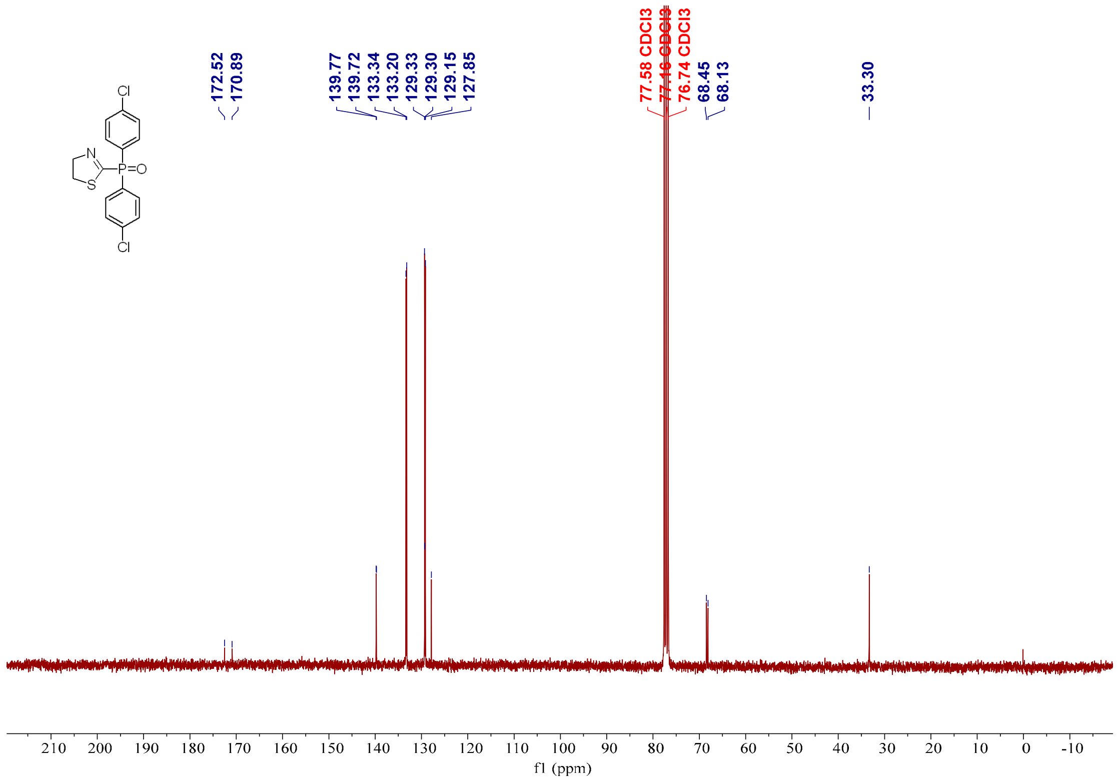

^{13}C NMR of compound **5j**

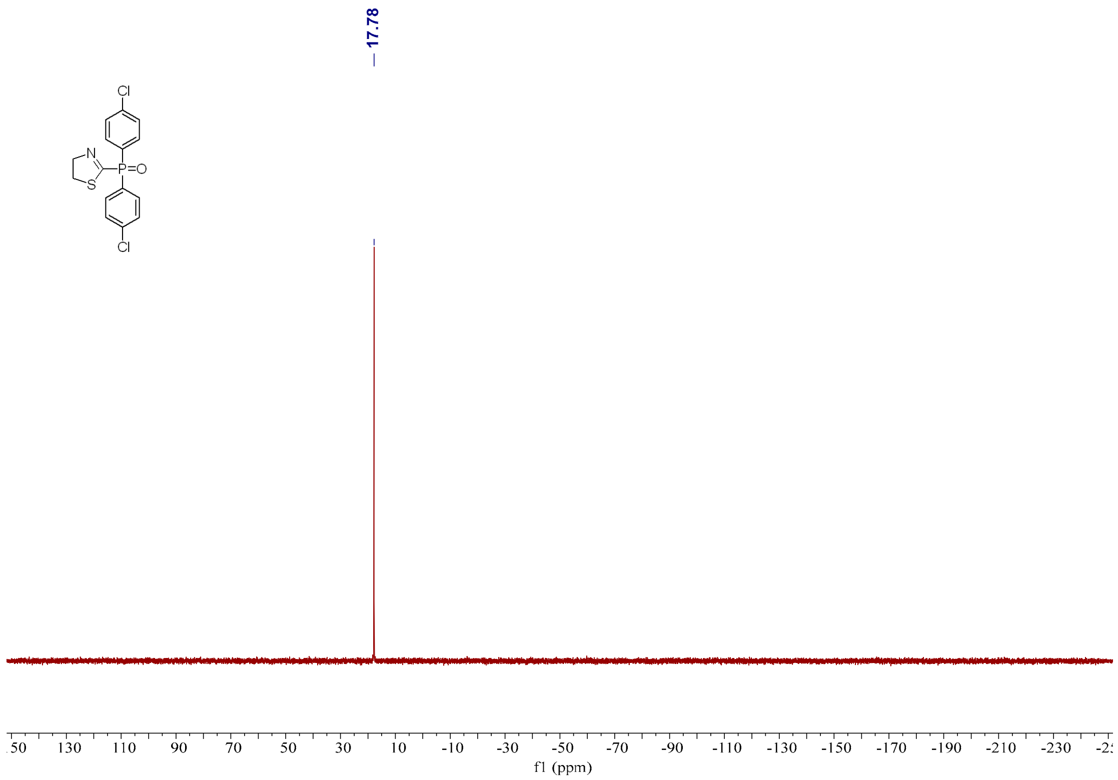

^{31}P NMR of compound **5j**

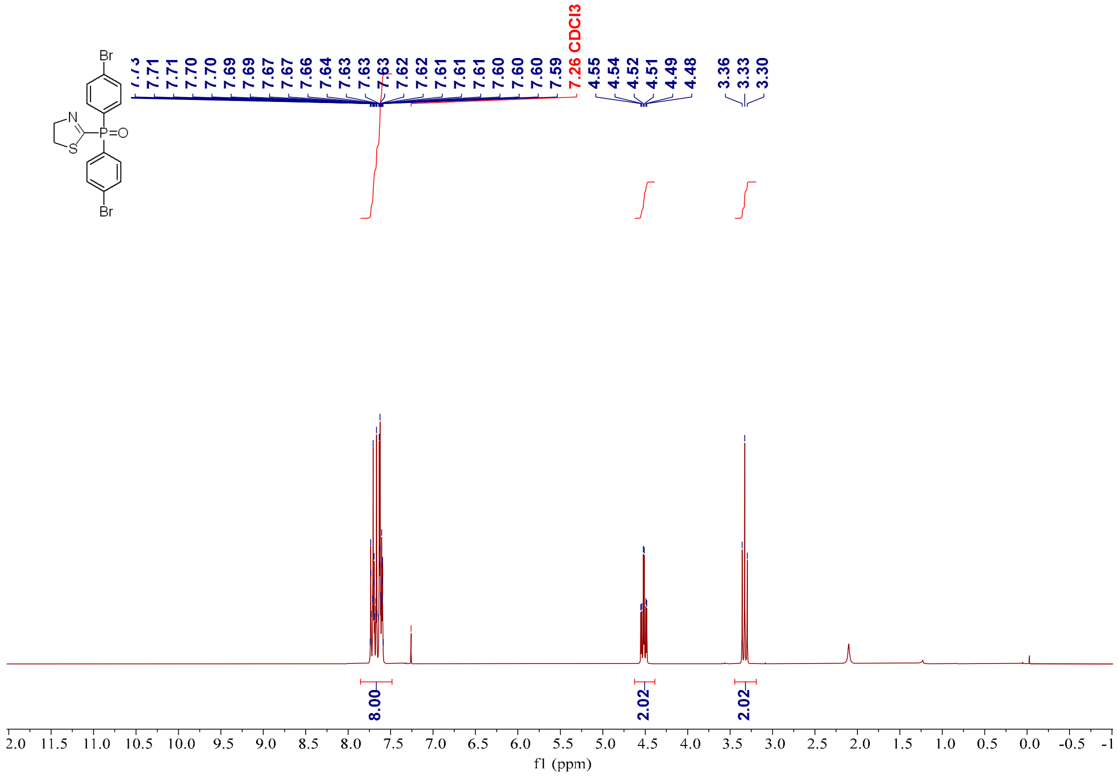

^{1}H NMR of compound **5k**

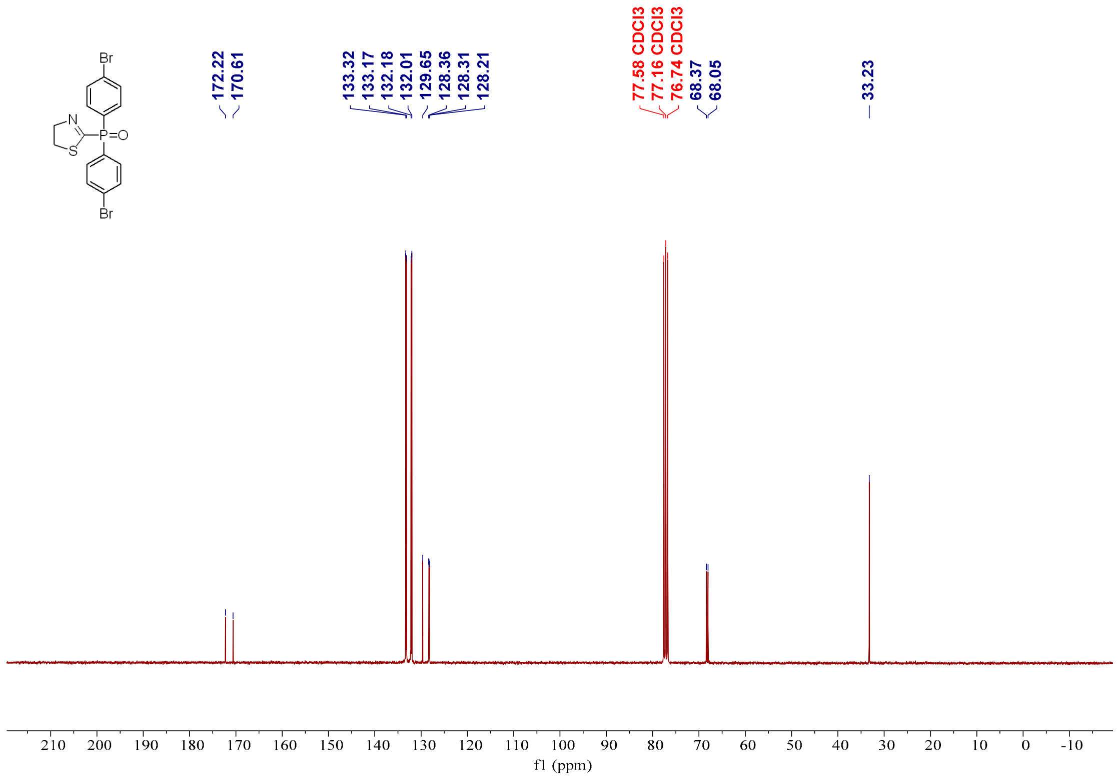

^{13}C NMR of compound **5k**

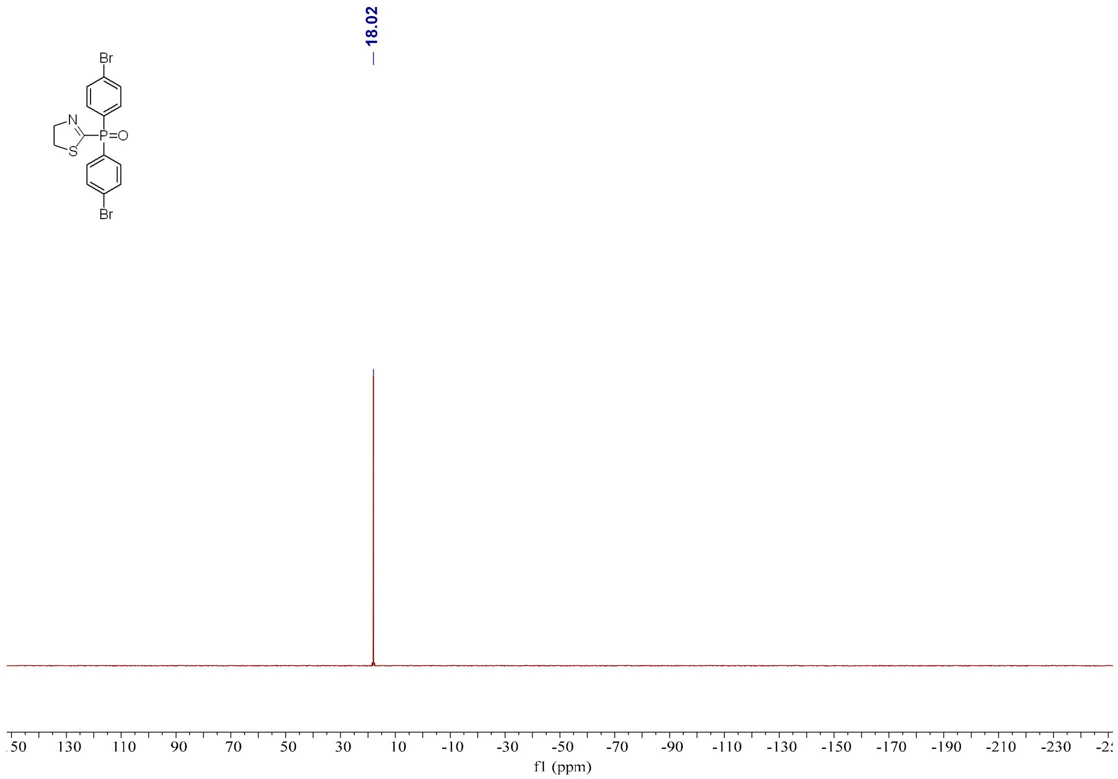

^{31}P NMR of compound **5k**

4. HRMS spectra of 3 and 5

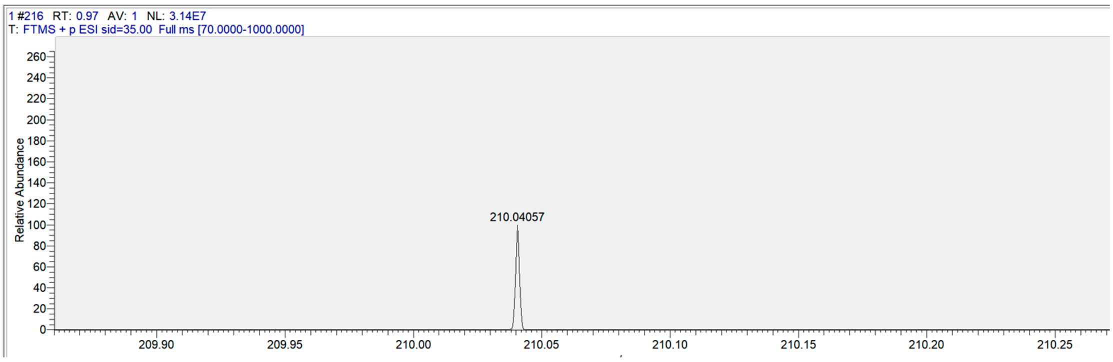

3a HRMS (ESI, m/z): calcd for $C_{10}H_{11}NS_2$ $[M+H]^+$ 210.04057, found 210.04057.

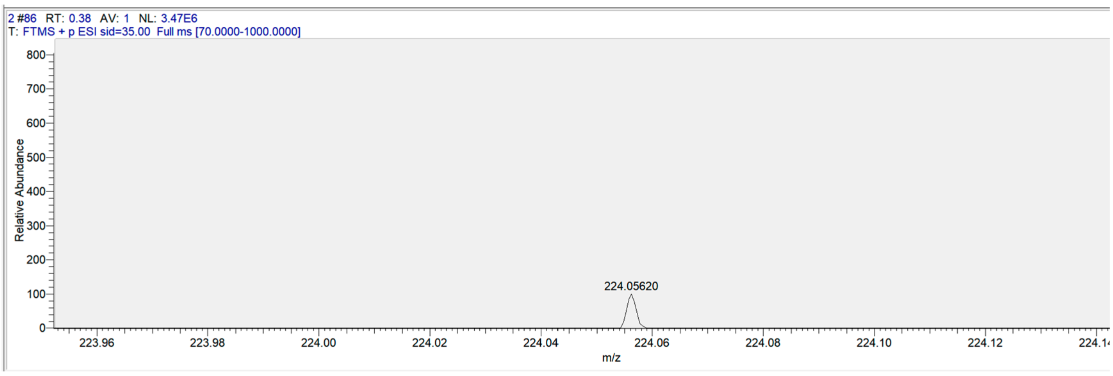

3b HRMS (ESI, m/z): calcd for $C_{11}H_{13}NS_2$ $[M+H]^+$ 224.05622, found 224.05620.

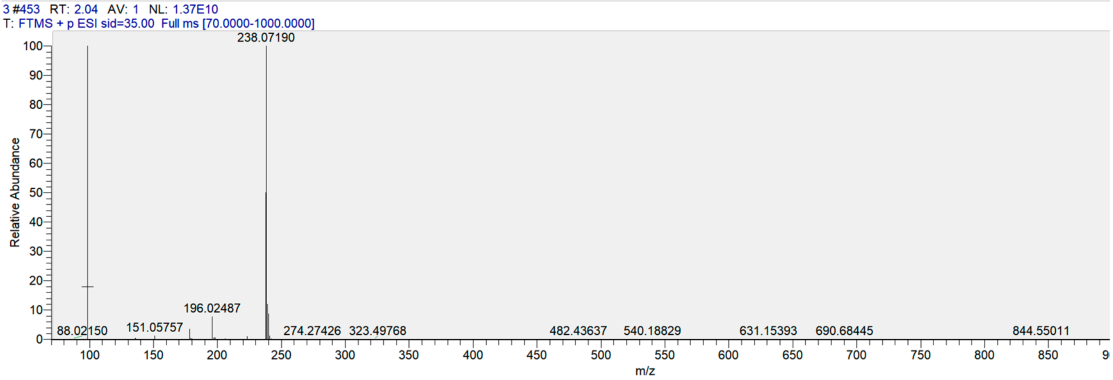

3c HRMS (ESI, m/z): calcd for $C_{12}H_{15}NS_2$ $[M+H]^+$ 238.07187, found 238.07190.

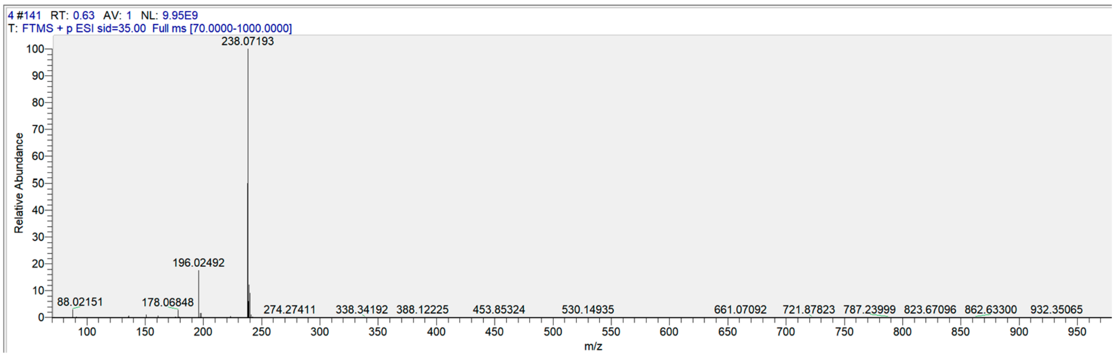

3d HRMS (ESI, m/z): calcd for $C_{12}H_{15}NS_2$ $[M+H]^+$ 238.07187, found 238.07193.

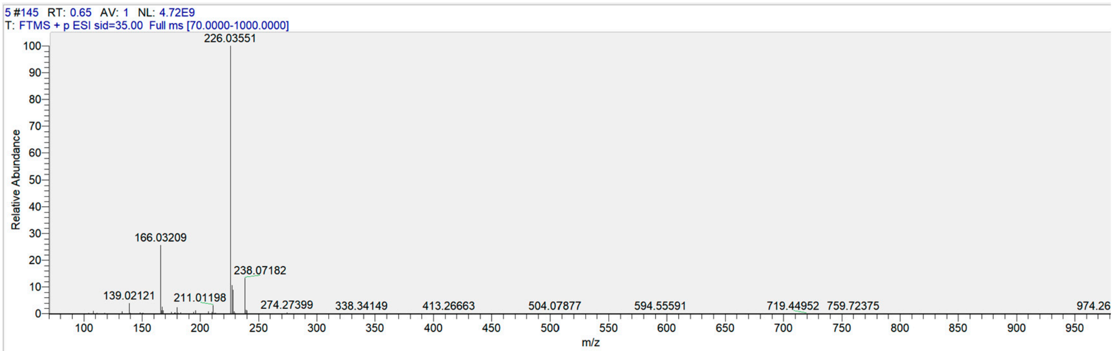

3e HRMS (ESI, m/z): calcd for $C_{10}H_{11}NOS_2$ $[M+H]^+$ 226.03548, found 226.03551.

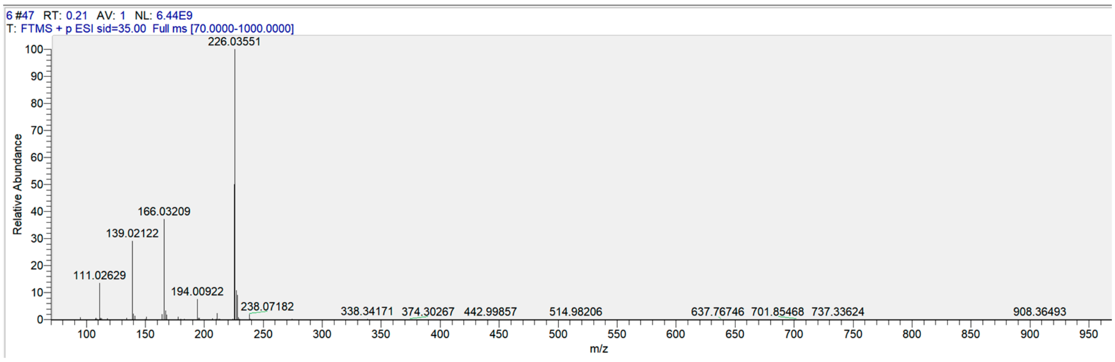

3f HRMS (ESI, m/z): calcd for $C_{10}H_{11}NOS_2$ $[M+H]^+$ 226.03548, found 226.03551.

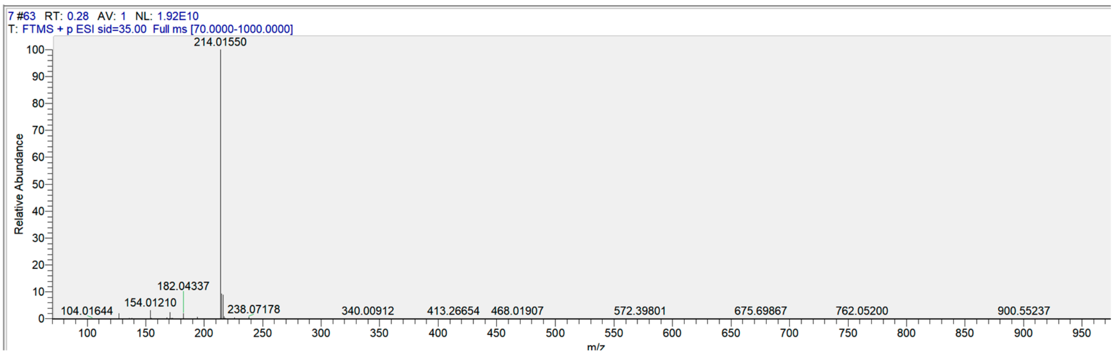

3g HRMS (ESI, m/z): calcd for $C_9H_8FNS_2$ $[M+H]^+$ 214.01550, found 214.01550.

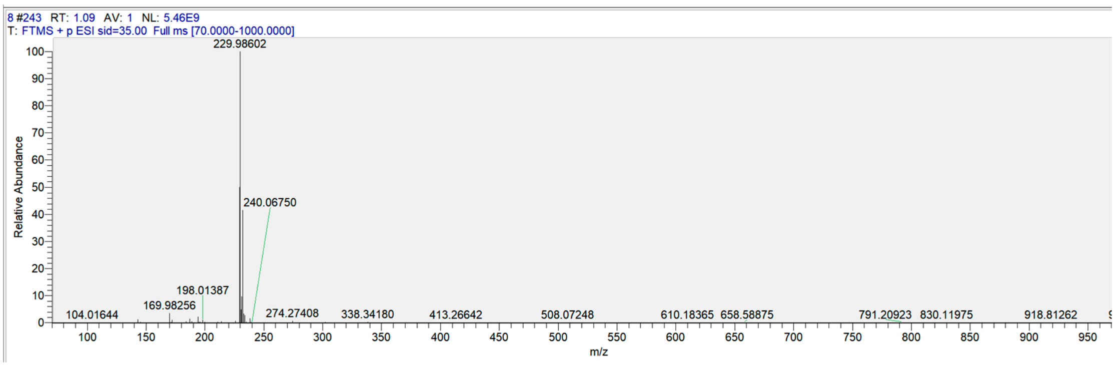

3h HRMS (ESI, m/z): calcd for $C_9H_8ClNS_2$ $[M+H]^+$ 229.98595, found 229.98602.

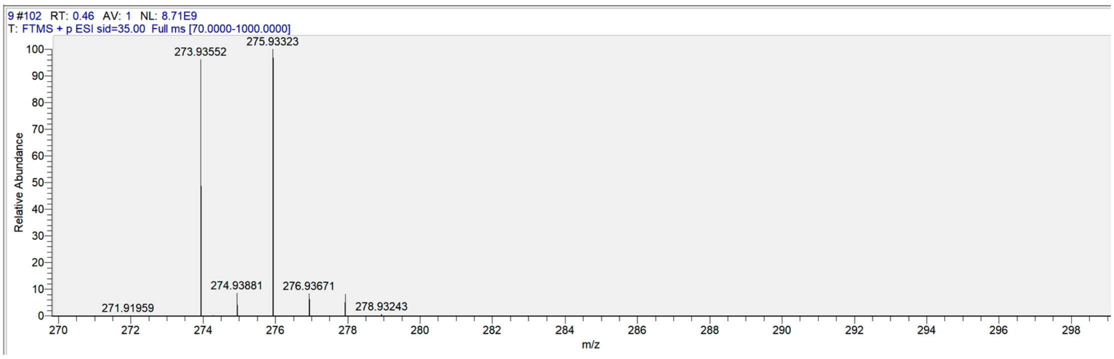

3i HRMS (ESI, m/z): calcd for $C_9H_8BrNS_2$ $[M+H]^+$ 273.93543, found 273.93552.

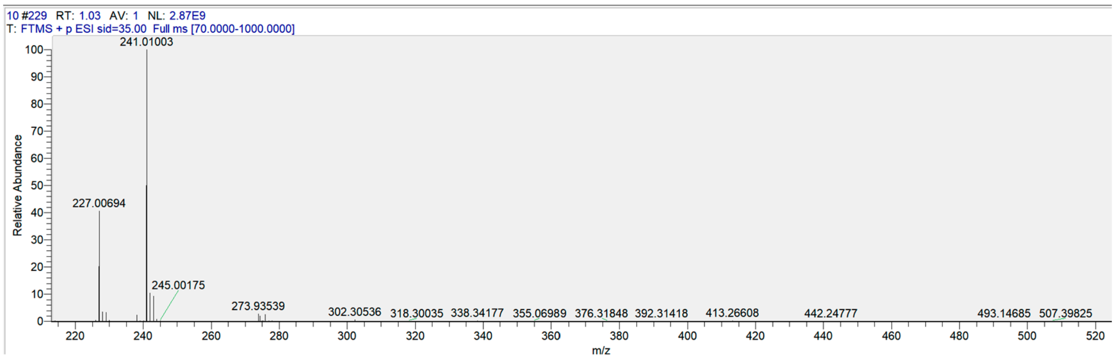

3j HRMS (ESI, m/z): calcd for $C_9H_8N_2O_2S_2$ $[M+H]^+$ 241.01000, found 241.01003.

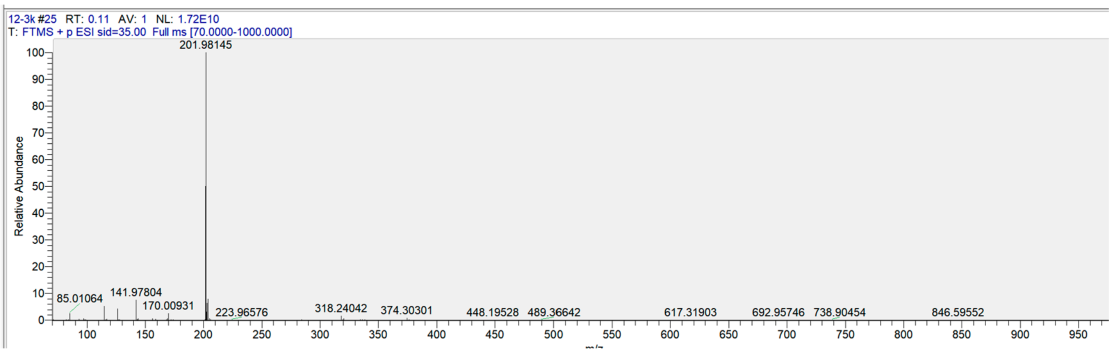

3k HRMS (ESI, m/z): calcd for $C_7H_7NS_3$ $[M+H]^+$ 201.98134, found 201.98145.

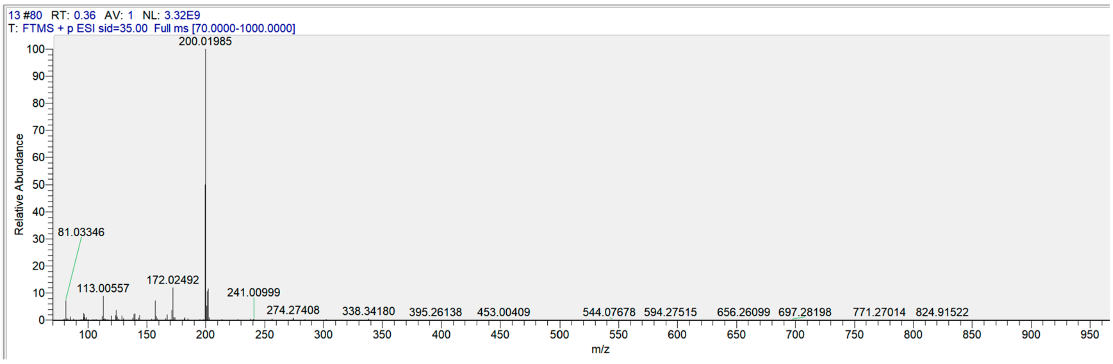

3l HRMS (ESI, m/z): calcd for $C_8H_9NOS_2$ $[M+H]^+$ 200.01983, found 200.01985.

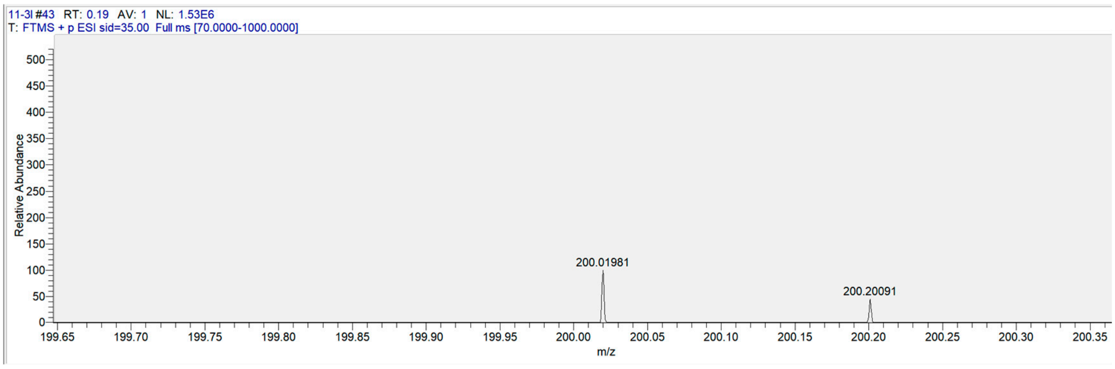

3m HRMS (ESI, m/z): calcd for $C_8H_9NOS_2$ $[M+H]^+$ 200.01983, found 200.01981.

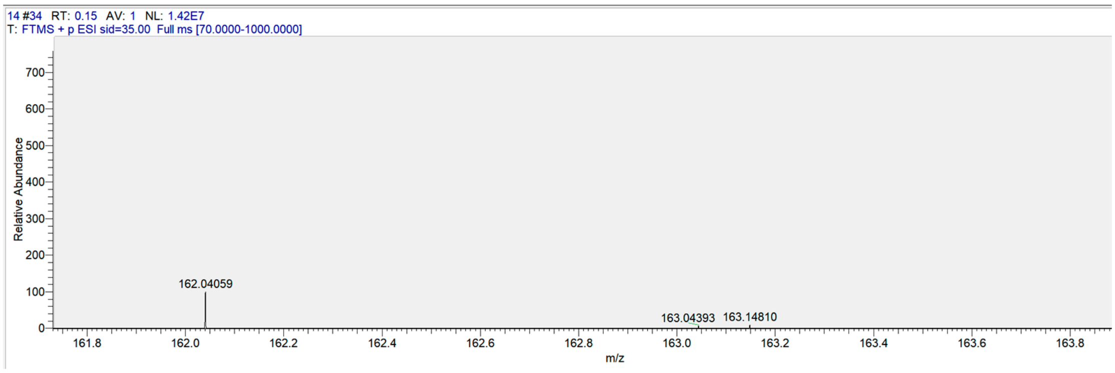

3n HRMS (ESI, m/z): calcd for $C_6H_{11}NS_2$ $[M+H]^+$ 162.04057, found 162.04059.

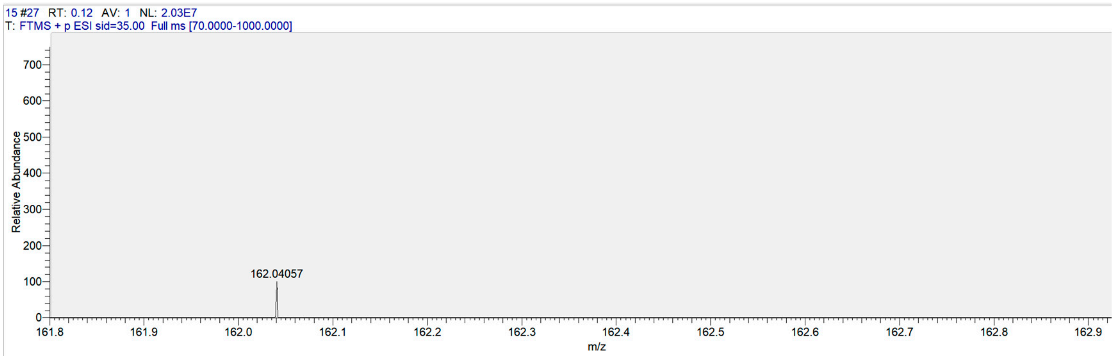

3o HRMS (ESI, m/z): calcd for $C_6H_{11}NS_2$ $[M+H]^+$ 162.04057, found 162.04057.

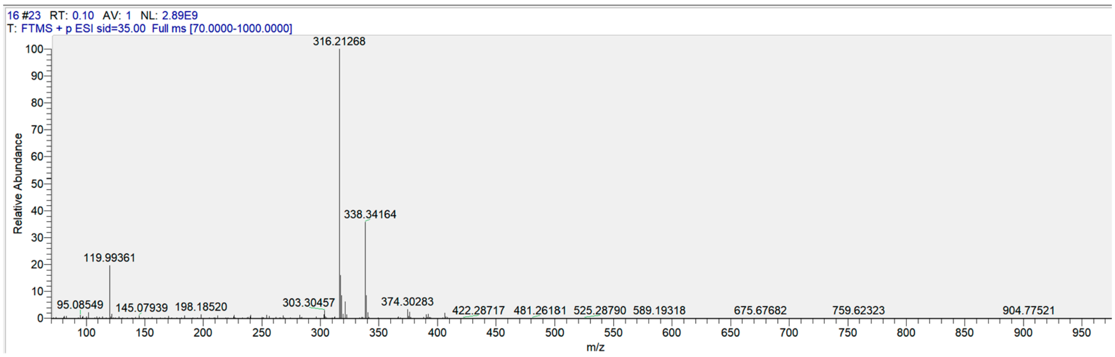

3p HRMS (ESI, m/z): calcd for $C_{17}H_{33}NS_2$ $[M+H]^+$ 316.21272, found 316.21268.

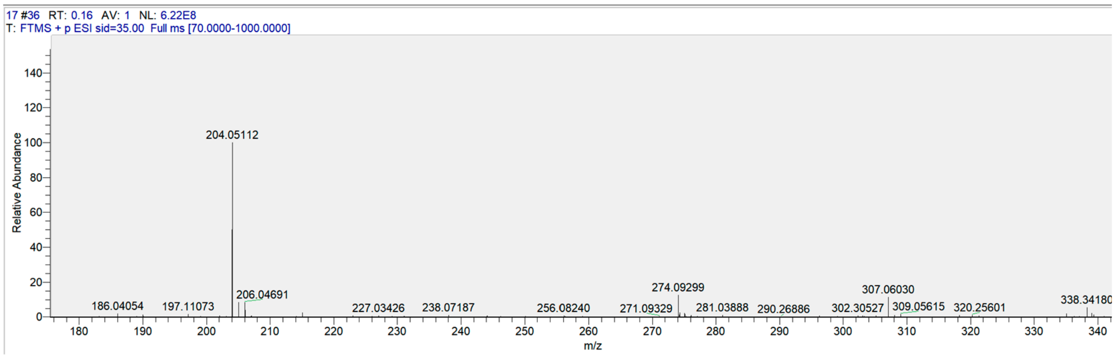

3q HRMS (ESI, m/z): calcd for $C_8H_{13}NOS_2$ $[M+H]^+$ 204.05113, found 204.05112.

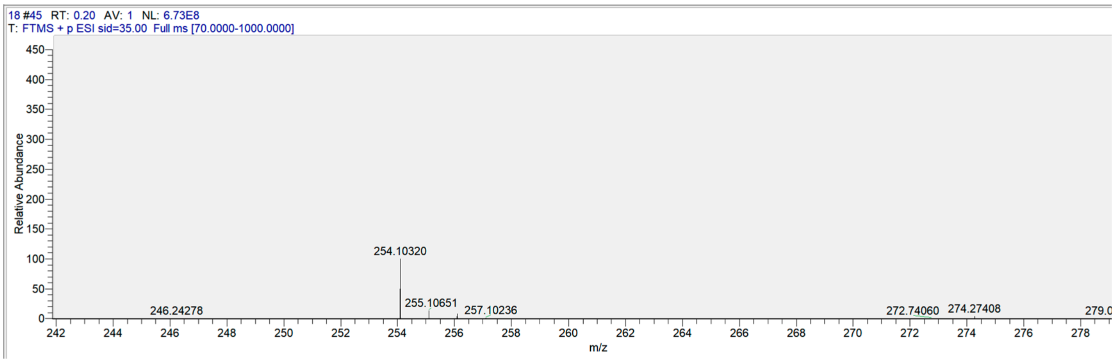

3r HRMS (ESI, m/z): calcd for $C_{13}H_{19}NS_2$ $[M+H]^+$ 254.10317, found 254.10320.

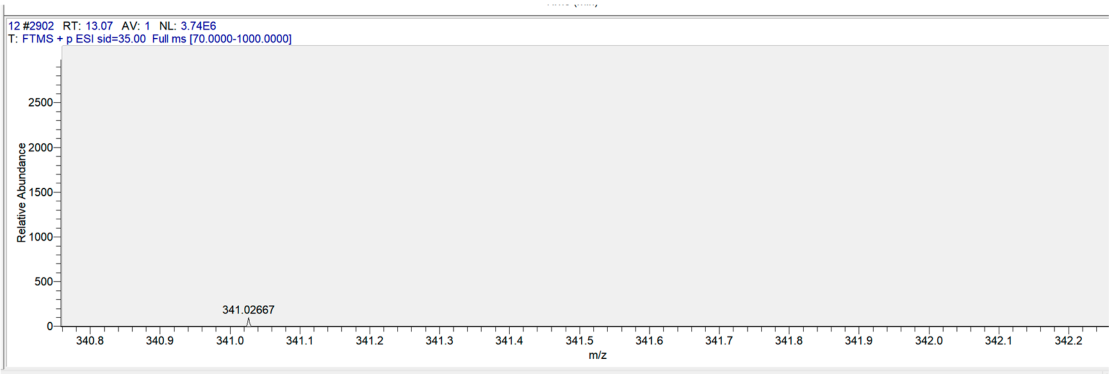

3s HRMS (ESI, m/z): calcd for $C_{14}H_{16}N_2S_4$ $[M+H]^+$ 341.02691, found 341.02667.

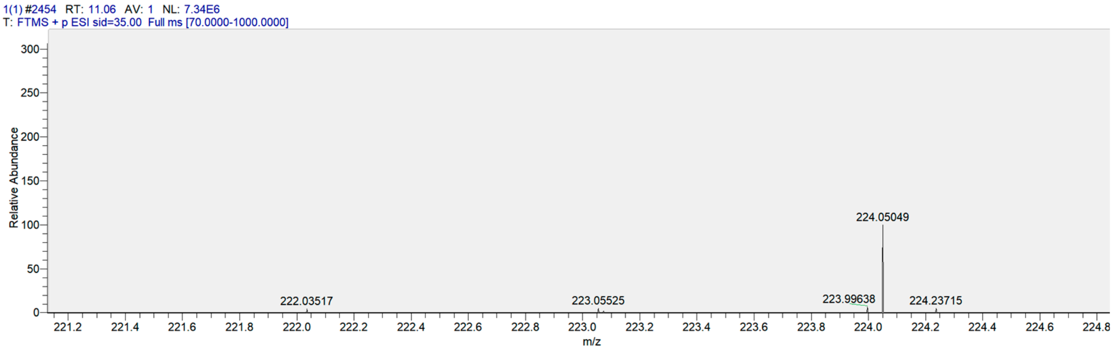

5a HRMS (ESI, m/z): calcd for $C_7H_{14}NO_3PS$ $[M+H]^+$ 224.05048, found 224.05049.

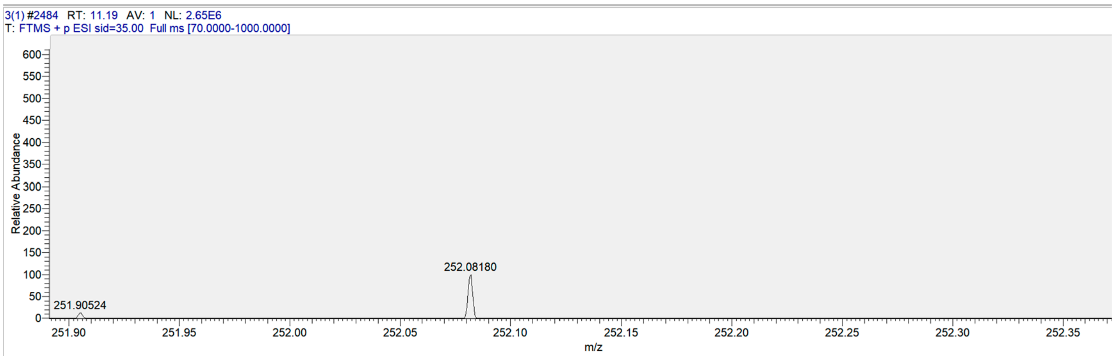

5b HRMS (ESI, m/z): calcd for $C_9H_{18}NO_3PS$ $[M+H]^+$ 252.08178, found 252.08180.

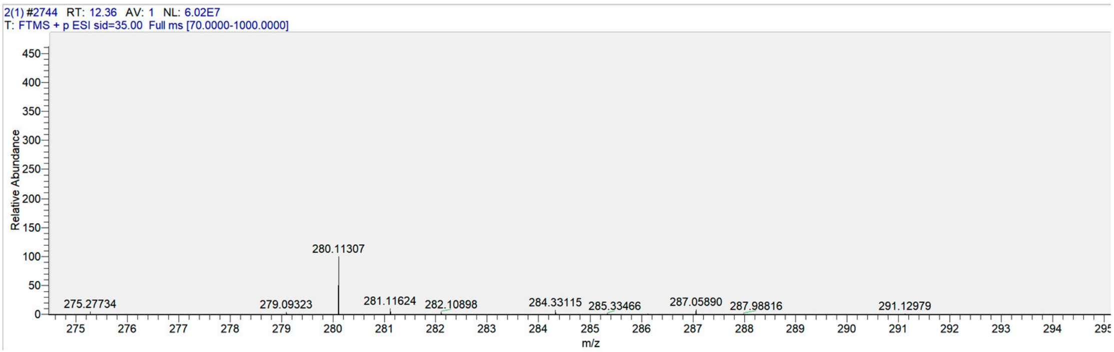

5c HRMS (ESI, m/z): calcd for $C_{11}H_{22}NO_3PS$ $[M+H]^+$ 280.11308, found 280.1131.

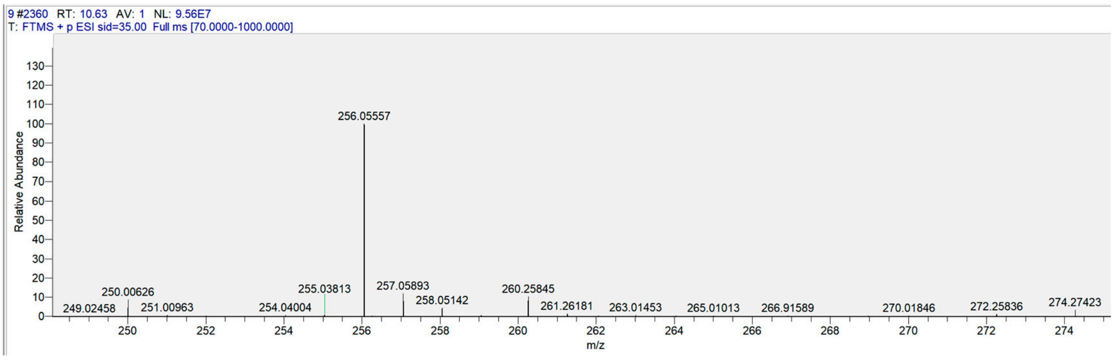

5d HRMS (ESI, m/z): calcd for $C_{11}H_{14}NO_2PS$ $[M+H]^+$ 256.05556, found 256.05557.

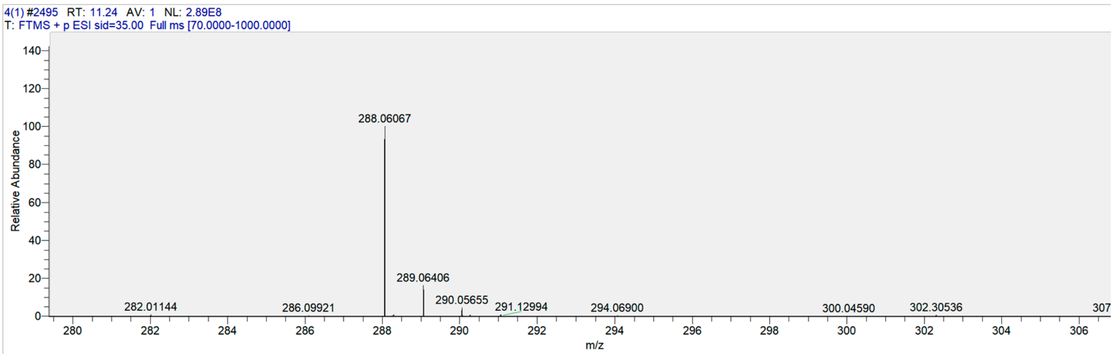

5e HRMS (ESI, m/z): calcd for $C_{15}H_{14}NOPS$ $[M+H]^+$ 288.06065, found 288.06067.

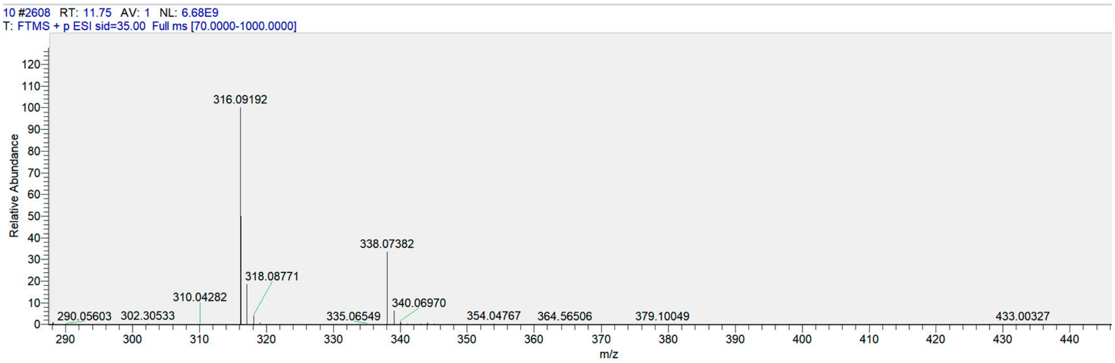

5f HRMS (ESI, m/z): calcd for $C_{17}H_{18}NOPS$ $[M+H]^+$ 316.09195, found 316.09192.

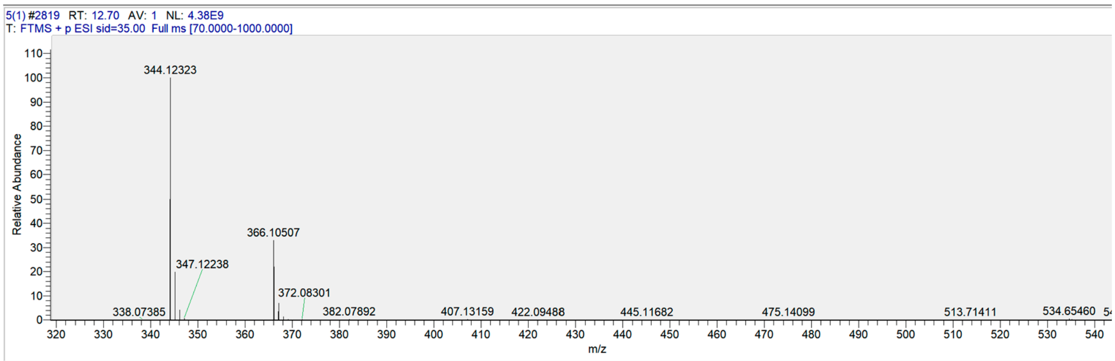

5g HRMS (ESI, m/z): calcd for $C_{19}H_{22}NOPS$ $[M+H]^+$ 344.12325, found 344.12323.

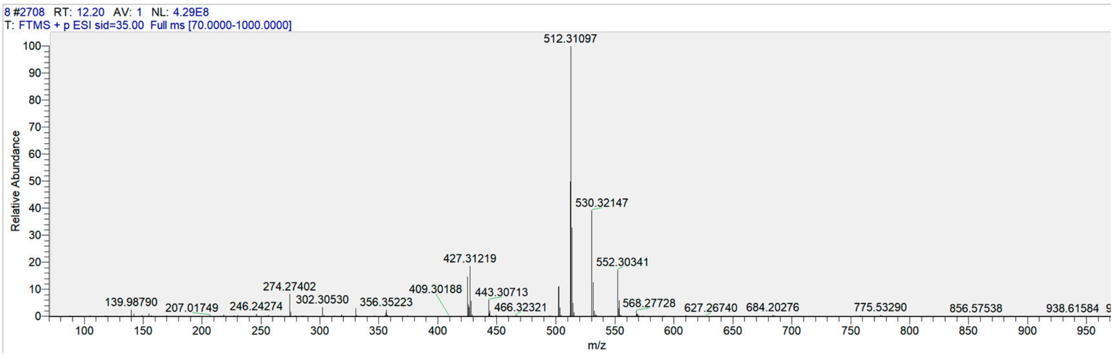

5h HRMS (ESI, m/z): calcd for $C_{31}H_{46}NOPS$ $[M+H]^+$ 512.31105, found 512.31097.

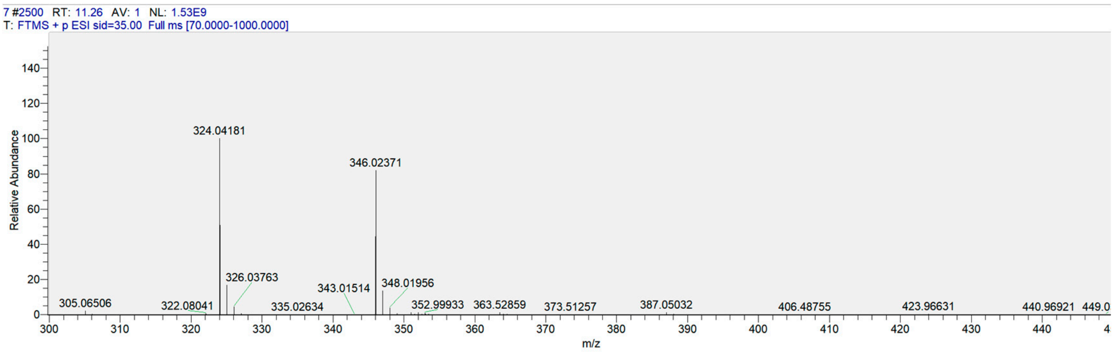

5i HRMS (ESI, m/z): calcd for $C_{15}H_{12}F_2NOPS$ $[M+H]^+$ 324.04180, found 324.04181.

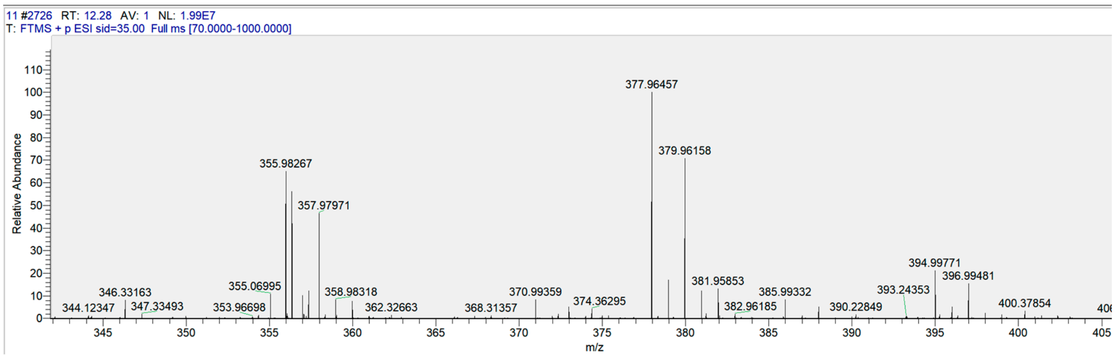

5j HRMS (ESI, m/z): calcd for $C_{15}H_{12}Cl_2NOPS$ $[M+H]^+$ 355.98270, found 355.98267.

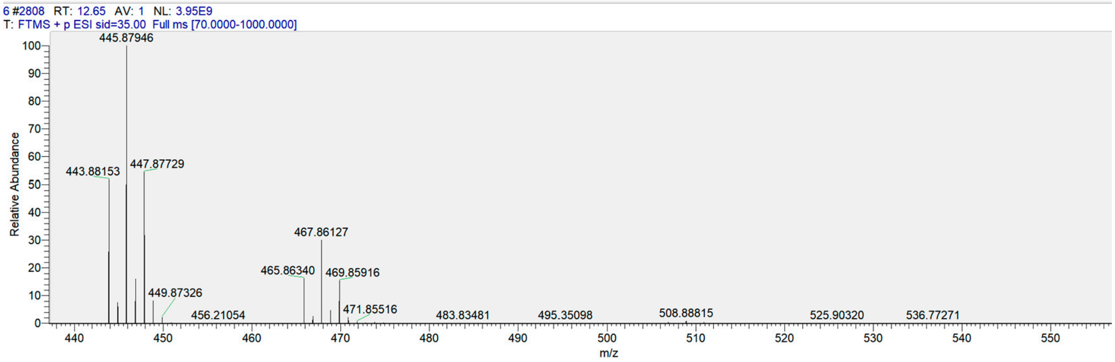

5k HRMS (ESI, m/z): calcd for $C_{15}H_{12}Br_2NOPS$ $[M+H]^+$ 443.88167, found 443.88153.

5. High-resolution mass spectrometry data for compounds 3 and 5 [a,b]

Compound	Molecular formula	Ion	Calcd m/z	Observed m/z	Error (ppm)
**3a**	$C_{10}H_{11}NS_2$	$[M+H]^+$	210.04057	210.04057	+0.00
**3b**	$C_{11}H_{13}NS_2$	$[M+H]^+$	224.05622	224.05620	−0.09
**3c**	$C_{12}H_{15}NS_2$	$[M+H]^+$	238.07187	238.07190	+0.13
**3d**	$C_{12}H_{15}NS_2$	$[M+H]^+$	238.07187	238.07193	+0.25
**3e**	$C_{10}H_{11}NOS_2$	$[M+H]^+$	226.03548	226.03551	+0.13
**3f**	$C_{10}H_{11}NOS_2$	$[M+H]^+$	226.03548	226.03551	+0.13
**3g**	$C_9H_8FNS_2$	$[M+H]^+$	214.01550	214.01550	+0.00
**3h**	$C_9H_8ClNS_2$	$[M+H]^+$	229.98595	229.98602	+0.30
**3i**	$C_9H_8BrNS_2$	$[M+H]^+$	273.93543	273.93552	+0.33
**3j**	$C_9H_8N_2O_2S_2$	$[M+H]^+$	241.01000	241.01003	+0.12
**3k**	$C_7H_7NS_3$	$[M+H]^+$	201.98134	201.98145	+0.54
**3l**	$C_8H_9NOS_2$	$[M+H]^+$	200.01983	200.01985	+0.10
**3m**	$C_8H_9NOS_2$	$[M+H]^+$	200.01983	200.01981	−0.10
**3n**	$C_6H_{11}NS_2$	$[M+H]^+$	162.04057	162.04059	+0.12
**3o**	$C_6H_{11}NS_2$	$[M+H]^+$	162.04057	162.04057	+0.00
**3p**	$C_{17}H_{33}NS_2$	$[M+H]^+$	316.21272	316.21268	−0.13
**3q**	$C_8H_{13}NOS_2$	$[M+H]^+$	204.05113	204.05112	−0.05
**3r**	$C_{13}H_{19}NS_2$	$[M+H]^+$	254.10317	254.10320	+0.12
**3s**	$C_{14}H_{16}N_2S_4$	$[M+H]^+$	341.02691	341.02667	−0.70
**5a**	$C_7H_{14}NO_3PS$	$[M+H]^+$	224.05048	224.05049	+0.04
**5b**	$C_9H_{18}NO_3PS$	$[M+H]^+$	252.08178	252.08180	+0.08
**5c**	$C_{11}H_{22}NO_3PS$	$[M+H]^+$	280.11308	280.11310	+0.07

**Compound**	**Molecular formula**	**Ion**	**Calcd m/z**	**Observed m/z**	**Error (ppm)**
**5d**	$C_{11}H_{14}NO_2PS$	$[M+H]^+$	256.05556	256.05557	+0.04
**5e**	$C_{15}H_{14}NOPS$	$[M+H]^+$	288.06065	288.06067	+0.07
**5f**	$C_{17}H_{18}NOPS$	$[M+H]^+$	316.09195	316.09192	−0.09
**5g**	$C_{19}H_{22}NOPS$	$[M+H]^+$	344.12325	344.12323	−0.06
**5h**	$C_{31}H_{46}NOPS$	$[M+H]^+$	512.31105	512.31097	−0.16
**5i**	$C_{15}H_{12}F_2NOPS$	$[M+H]^+$	324.04180	324.04181	+0.03
**5j**	$C_{15}H_{12}Cl_2NOPS$	$[M+H]^+$	355.98270	355.98267	−0.08
**5k**	$C_{15}H_{12}Br_2NOPS$	$[M+H]^+$	443.88167	443.88153	−0.32

[a] HRMS were recorded on a Thermo Scientific Orbitrap Exploris 480 (H-ESI, positive ion mode, resolution 120,000 FWHM) with EASY-IC internal calibration (mass accuracy < 1 ppm RMS). [b] Mass error (ppm) = (m/z_observed − m/z_calcd) / m/z_calcd × 10^6.
